# The Antiproliferative Activity and NO Inhibition of *Neo*-Clerodane Diterpenoids from *Salvia guevarae* in RAW 264.7 Macrophages

**DOI:** 10.3390/molecules30071628

**Published:** 2025-04-05

**Authors:** Juan Pablo Torres-Médicis, Celia Bustos-Brito, Leovigildo Quijano, Brenda Y. Bedolla-García, Sergio Zamudio, Teresa Ramírez-Apan, Diego Martínez-Otero, Baldomero Esquivel

**Affiliations:** 1 Instituto de Química, Universidad Nacional Autónoma de México, Circuito Exterior, Ciudad Universitaria, Ciudad de México 04510, Mexicoquijano@unam.mx (L.Q.);diegomtz@unam.mx (D.M.-O.); 2 Instituto de Ecología, A.C., Centro Regional del Bajío, Apartado Postal 386, Pátzcuaro 61600, Mexico; brenda.bedolla@inecol.mx; 3 Independent Researcher, Apartado Postal 392, Pátzcuaro 61600, Mexico

**Keywords:** *Salvia guevarae*, *neo*-clerodanes diterpenoids, RAW 264.7 macrophages

## Abstract

In this study, nine *neo*-clerodane-type diterpenoids (**1**–**9**) were isolated from the dichloromethane extract of *Salvia guevarae* Bedolla & Zamudio leaves. Compounds **1**–**6** were new natural products, and **7**–**9** were acetone artifacts. In addition, four *neo*-clerodanes diterpenoids (**10**–**13**) previously described from different sources and six triterpenoids—identified as 3β,20,25-trihydroxylupane, oleanolic acid, 3β-*O*-acetyl-oleanolic acid, ursolic acid, 3β-*O*-acetyl-betulinic acid, and 3β,28-*O*-diacetyl-betulin—were isolated. Additionally, five flavonoids were also isolated from the methanol extract: quercetin-3-*O*-β-xylopyranosyl-(1 → 2)-β-galactopyranoside, taxifolin-7-*O*-β-glucopyranoside, naringenin-7-*O*-β-glucopyranoside, a mixture of 2*R* and 2*S* eriodictyol-7-*O*-β-glucopyranoside, caffeic acid, the methyl ester of rosmarinic acid, and rosmarinic acid. The structure of the isolated compounds was established by spectroscopic means, mainly ^1^H and ^13^C NMR, including 1D and 2D homo- and heteronuclear experiments. The absolute configuration of **1** and **10** was ascertained via an X-ray analysis, and that of the other compounds via ECD. The antiproliferative activity of some diterpenoids was determined using the sulforhodamine B method, where guevarain B (**2**) and 6α-hydroxy-patagonol acetonide (**7**) showed moderate activity against the K562 line, with IC_50_ (μM) = 33.1 ± 1.3 and 39.8 ± 1.5, respectively. The NO inhibition in RAW 264.7 macrophage activity was also determined for some compounds, where 2-oxo-patagonal (**6**), 6α-hydroxy-patagonol acetonide (**7**), and 7α-acetoxy-*ent*-clerodan-3,13-dien-18,19:16,15-diolide (**10**) were proven to be active, with IC_50_ (μM) of 26.4 ± 0.4, 17.3 ± 0.5, and 13.7 ± 2.0, respectively. The chemotaxonomy of *Salvia guevarae* is also discussed.

## 1. Introduction

*Salvia* L. (Lamiaceae) is one of the most species-rich genera of angiosperm plants, with about 1000 species with a sub-cosmopolitan distribution [1]. Recent taxonomic and phylogenetic analyses have led to the organization of the genus into 11 subgenera [2], with the subgenera *Audibertia* and *Calosphace* being endemic to the American continents. With a biogeographic distribution ranging from North America to Central America, the Caribbean, and South America, the subgenus *Calosphace*, with an estimated 550 species, is the most diverse, comprising 55% of the whole genus. The *Salvia* species growing in Mexico mainly belong to this subgenus. With an estimated 312 species, with 57% of species from the subgenus *Calosphace* and 31% from the subgenus Mundial, *Salvia* is the most species-rich genus in Mexico, which is considered, due to this diversity and an estimated endemism of 82%, the center of the origin and diversification of this subgenus [3]. Several *Salvia* species are widely cultured in gardens around the world and in Mexico and profusely used as medicinal plants for different ailments including gastrointestinal, nervous system, gynecological, and folk illnesses [4].

From systematic phytochemical studies on Mexican *Salvia* species, starting in 1984, diterpenoids have been found to be the most diversified secondary metabolites. Diterpenoids with *neo*-clerodane-type (sometimes also referred to as *ent*-clerodanes) [5] and abietane-type skeletons are the most frequently isolated, although pimarane-, totarane-, and recently labdane-type diterpenoids [6] have also been described from some members of the genus. Several rearranged scaffolds derived mainly from the *neo*-clerodane type and, to a lesser extent, the abietane- and pimarane-type skeletons, have also frequently been described, highlighting the chemical features of Mexican *Salvia* species. The distribution of these types of compounds, in some cases, parallels that of the botanical classification proposed by Epling, as in the case of sections *Erythrostachys*, *Fulgentes*, *Angulatae*, *Tomentellae*, and *Scorodonia*. Another interesting result is that the distribution of diterpenoids in species of the subgenus *Calosphace* differs markedly from that found in species from Europe and Asia, from which mainly abietane and abietane-derived diterpenoids have been isolated. The presence of diterpenoids in the genus *Salvia* is, therefore, of chemo-systematic relevance [7].

As a result of intensive botanical field work during the past three decades all over the country, the number of *Salvia spp.* has constantly grown in Mexico, with several new species being described, all belonging to the subgenus *Calosphace*. Since 2007, 62 new species have been described, some of which have been classified up to the taxonomic section rank [5]. *Salvia guevarae* Bedolla & Zamudio was recently described and determined to be a new botanical species [8]. The morphology of this species, with its red markedly ventricose corollas with short lips, allowed it to be classified in section *Holwaya* (Ramamoorthy) [9] and suggests its close relation to *S. involucrata* and *S. puberula*. Recently, we described the diterpenoid content of the newly described *S. carranzae* Bedolla & Zamudio, which led us to propose that this species is closely related to those classified in section *Erythrostachys* (Epling) [7]. In this study, we report on the phytochemical analysis of *S. guevarae*, supporting its inclusion in section *Holwaya*, and evaluate the biological activity of some isolated secondary metabolites. The dichloromethane and methanol extracts of this species were thoroughly analyzed using chromatographic techniques to afford nine not previously described *neo*-clerodane-type diterpenoids, with three of them being acetone artifacts, four *neo*-clerodane diterpenoids, six triterpenoids, and eight polyphenolic compounds, already known. Their antiproliferative activities against human cancer cell cultures and capacity for the inhibition of nitric oxide (NO) production in RAW 264.7 macrophages were also evaluated.

## 2. Results and Discussion

### 2.1. Characterization

**Figure 1:**
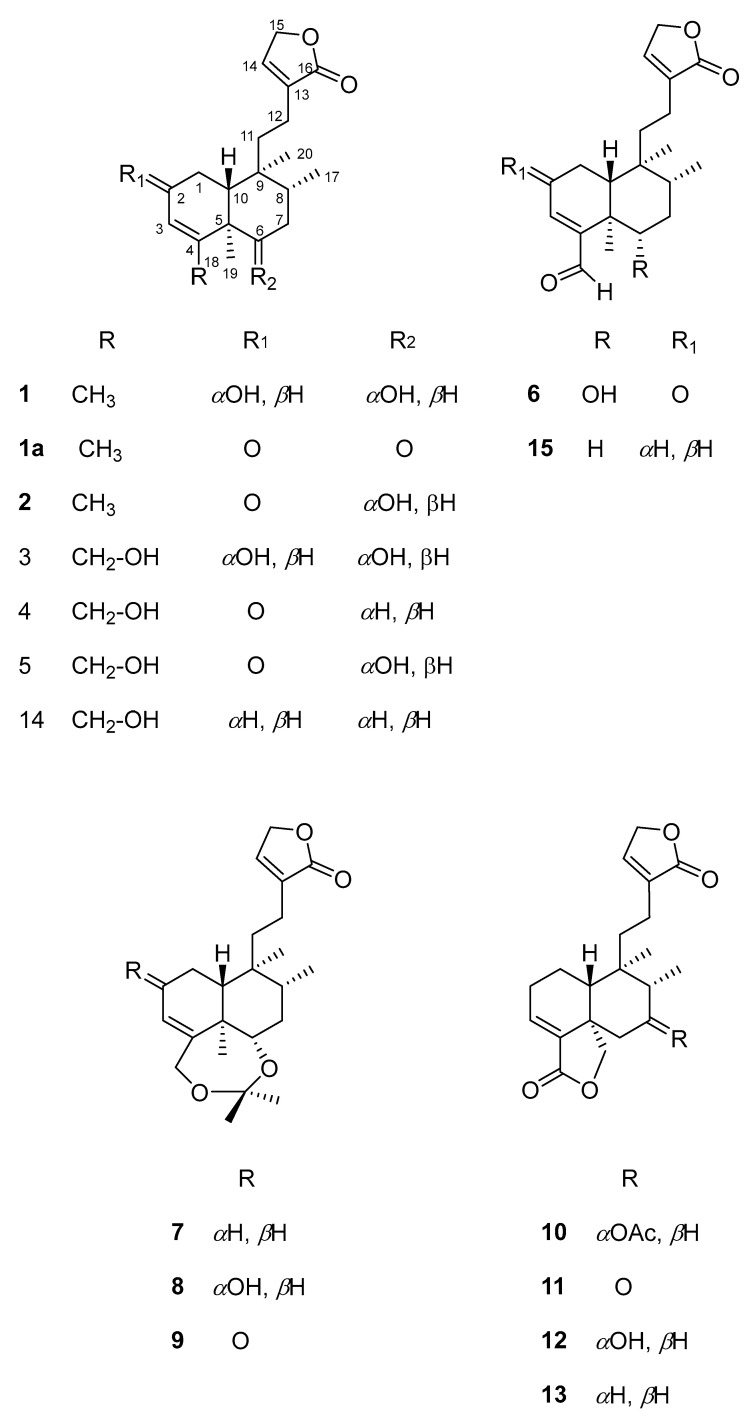
Compounds isolated from *S. guevarae*.

The leaves and flowers of *S. guevarae* afforded, after dichloromethane extraction and thorough chromatographic purification, 13 *neo*-clerodane derivatives (**1**–**13**) (Figure 1). Compounds **1**–**6** are undescribed *neo*-clerodane diterpenoids with structures related to those of patagonol (**14**) and patagonal (**15**), two *neo*-clerodane diterpenoids previously isolated from a liana collected from the Surinam rainforest, tentatively identified as a *Casimirella* species [10]. Diterpenoids **7**–**9** are artifacts obtained, most likely, via a reaction with acetone, used in the purification process, and products **10**–**13** are previously known compounds isolated from several sources. The structural elucidation of all the isolated compounds was carried out via spectroscopic and chemical methods and a comparison with literature data.

Compound **1** was the major component and was isolated as a crystalline solid at mp 136–138 °C. The HR-DART-MS showed a *pseudo*-molecular ion [M + H − H_2_O]^+^ at *m*/*z* 317.2103 (calculated for C_20_H_30_O_4_ + H − H_2_O, 317.2117) consistent with the molecular formula C_20_H_30_O_4_ minus the loss of an H_2_O molecule from the molecular ion. Its IR spectrum exhibited characteristic absorptions for α,β-unsaturated-γ-lactone function (1755 cm^−1^), hydroxyl groups (3606 cm^−1^), and double bounds (1654, 1602 cm^−1^). The UV spectrum [MeOH, λ_max_ (log ε) 207 (6.32) nm] was consistent with these assignments.

**Figure 2:**
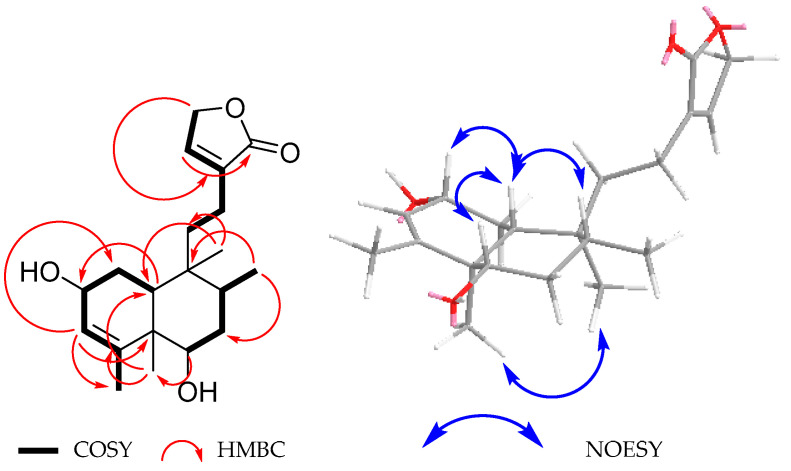
Selected COSY, HMBC, and NOESY correlations for **1**.

In the ^1^H NMR spectrum of **1** (Table 1), signals of an α-substituted butenolide were observed at C-12. A quintet at *δ*_H_ 7.41 (*J* = 1.7 Hz) was assigned to the vinylic H-14 coupled with two methylene groups with signals at *δ*_H_ 4.79 and 2.14/2.05 due to CH_2_-15 and CH_2_-12, respectively, and with similar ^3^*J* and ^4^*J* coupling constant values to those of H-14 and H-12, according to their COSY spectra (Table 1, Figure 2). The ^13^C NMR spectrum of **1** (Table 1) supports the above assumption, since the corresponding signals of C-13, C-14, C-15, and C-16 were observed at *δ*_C_ 134.3, 146.2, 71.0, and 174.7, respectively, which is in accordance with the results obtained from the HSQC and HMBC experiments (Figure 2). The presence of an α-substituted butenolide group is a frequent feature in *neo*-clerodane-type diterpenoids isolated from *Salvia* species [9] and is present in all diterpenoids (**1**–**13**) isolated from *S. guevarae.*

The other relevant signals observed in the ^1^H NMR spectrum of **1** (Table 1) included a broad singlet at *δ*_H_ 5.19, which was ascribed to the vinylic H-3, and multiple signals at *δ*_H_ 4.13, assigned to the allylic proton H-2, geminal to the hydroxyl group at the C-2 position. In the COSY spectrum of **1** (Figure 2), cross peaks of correlation were observed between the H-2 signal (*δ*_H_ 4.13) with H-3 (*δ*_H_ 5.19); a three-proton signal at *δ*_H_ 1.84; and the hydrogens of a methylene group at *δ*_H_ 1.94 (ddd, *J* = 12.6, 7.0, 1.4 Hz) and 1.49 (td, *J* = 12.6, 10.1 Hz), which were ascribed to the vinylic CH_3_-18 and to H-1 *equatorial* and H-1 *axial*, respectively. All other relevant COSY interactions are depicted in Figure 2.

The ^1^H NMR spectrum of **1** (Table 1) also exhibited the signal for a second hydrogen atom geminal to a hydroxyl group, which was observed at *δ*_H_ 3.48 (dt, *J* = 9.5, 5.6 Hz). The multiplicity and the coupling constant values led us to propose that the hydroxyl group is located at the C-6 position with an α *equatorial* orientation. Signals due to characteristic C-17, C-19, and C-20 methyl groups of a *neo*-clerodane-type diterpenoid were also observed at *δ*_H_ 0.84 (d, *J* = 6.8 Hz), 1.06 (s), and 0.75 (s), respectively. Finally, a doublet of doublets at *δ*_H_ 1.35 (*J* = 12.6, 1.4 Hz), ascribed to the β-*axially* oriented H-10, coupled with the methylene signals of -CH_2_-1 at δ_H_ 1.94 (ddd, *J* = 12.6, 7.0, 1.4 Hz)/1.49, td (*J* = 12.6, 10.1 Hz), was also observed in the ^1^H NMR spectrum of **1** (Table 1). These facts, in combination with the chemical shifts observed for C-17 and C-20 in the ^13^C NMR spectrum of **1** (Table 1), indicated a *trans* fusion of A/B rings, which is the most frequently observed for the *neo*-clerodane diterpenoids hitherto isolated from *Salvia* spp. [5]. All other signals in the ^13^C NMR spectrum of **1** (Table 1) agreed with the proposed structure, and all connectivities were confirmed with the aid of the HMBC spectrum (Figure 2).

The NOESY spectrum supports relative stereochemistry since it showed NOE correlations between the β-*axially* oriented H-10 and H-2 (Figure 2), indicating that the latter must also be β-*axially* oriented; therefore, the C-2 hydroxyl group must be α-*equatorially* oriented. On the other hand, the cross peaks of correlation between H-10 and H-8 established that the C-17 methyl group was also α-*equatorially* oriented. The NOE effect observed between the C-19 and C-20 methyl groups indicated that both were cofacial [10], as shown in Figure 1. Therefore, compound **1** corresponds to a new *neo*-clerodane compound that we named guevarain A.

**Figure 3:**
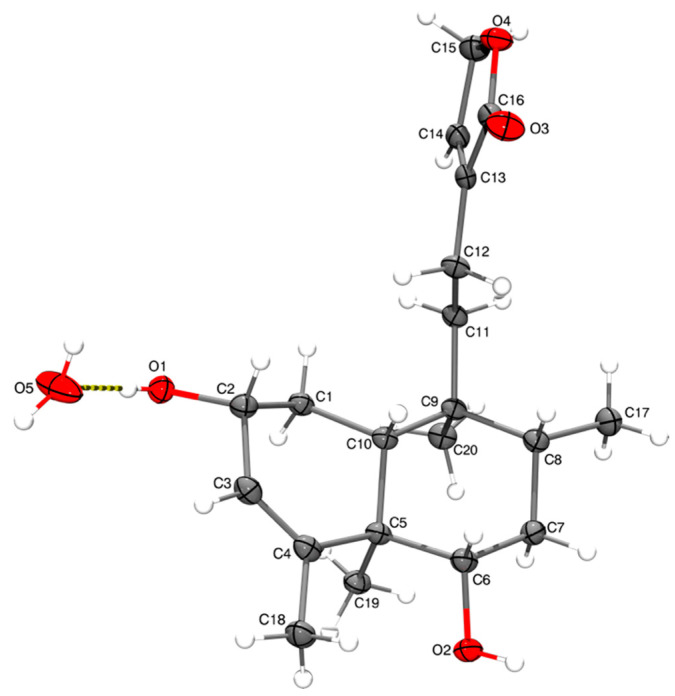
The molecular structure of compound **1**. The thermal ellipsoids are set at 50% of the probability level.

**Figure 4:**
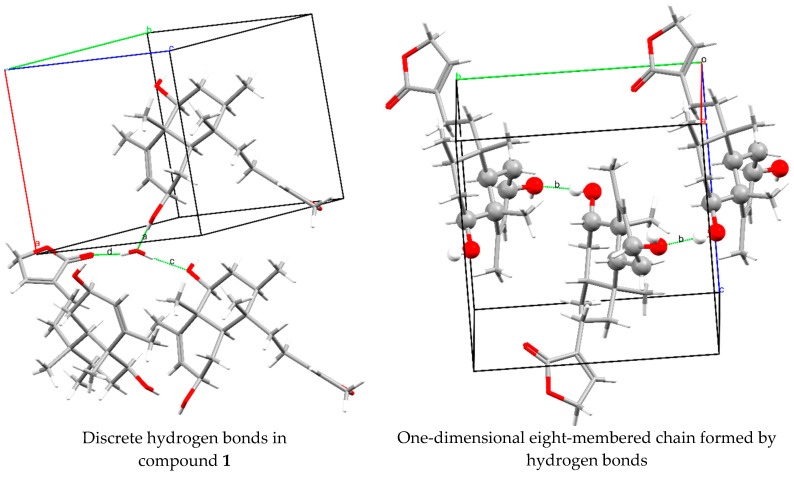
Hydrogen bonds in the packing structure found in compound **1**.

The absolute configuration of guevarain A was established via an X-ray diffraction analysis. Guevarain A was crystallized as a monohydrate in the monoclinic P2(1) space group, and the absolute configuration was determined to have the following parameters: Flack(x), −0.02(3); Hooft(y), −0.02(2); and Parsons(z), −0.019(16). This configuration confirms the assignment of the crystal model as well as the configuration of the chiral carbon atoms as being 2*R*, 5*R*, 6*S*, 8*R*, 9*S,* and *10R*. The molecular structure was shown in Figure 3, and the crystallographic details are presented in the Supplementary Materials (Table S1). Figure 4 shows the crystalline packing, where three discrete hydrogen bonds described as $D_{1}^{1}$(2) were found, labeled as a, c, and d, where one water molecule participate as donor (c, d) and one (a) as an acceptor. An eight-membered one-dimensional chain, described as $C_{1}^{1}$(8) in terms of graph set descriptors, labeled as b, formed through the O-H fragments of compound **1**. The hydrogen bond distances and angles corresponding to guevarain A (**1**) are shown in the Supplementary Materials (Table S2).

**Figure 5:**
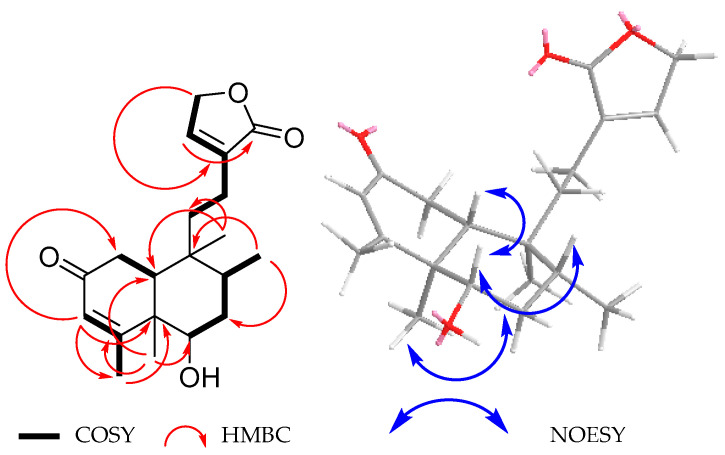
Selected COSY, HMBC, and NOESY correlations for **2**.

Diterpenoid **2**, C_20_H_28_O_4_, (HR-DART-MS), showed a similar IR spectrum to that of guevarain A (**1**), except for the presence of an intense band at 1644 cm^−1^ due to an α,β-unsaturated carbonyl group, also supported by the UV spectrum, since a λ_max_ at 239.8 nm (log ε = 4.07) was observed. The ^1^H and ^13^C NMR spectra of **2** (Table 1) confirmed the presence of a C2-C3-C4 α,β-unsaturated ketone, given that a broad singlet at *δ*_H_ 5.72, ascribed to the vinylic H-3, was found to be coupled to a three-proton doublet at *δ*_H_ 2.14 (*J* = 1.3 Hz), assigned to C-18 vinylic methyl, in the ^1^H NMR spectrum. Signals for C-2 carbonyl, C-3:C-4 double bond, and C-18 methyl were observed at *δ*_C_ 199.7, 126.5, 172.8, and 23.0, respectively, in the ^13^C NMR spectrum. All these assignments were supported by COSY and HMBC spectra (Figure 5).

The signal for the hydrogen geminal to the hydroxyl group at C-6 in compound **2** was observed at δ_H_ 3.70 (*J* = 11.1, 4.8 Hz), similarly to that of **1**. The main difference in the ^1^H NMR spectrum was in the signals associated with the presence of a carbonyl group at C-2. Thus, in the COSY spectrum of compound **2** (Figure 5), the CH_2_-1 signals at δ_H_ 2.48 (*J* = 17.8, 13.9 Hz) and 2.32 (*J* = 17.8, 3.5 Hz) showed couplings only with the doublet of doublets at δ_H_ 1.88 (*J* = 13.9, 3.6 Hz) ascribed to the β-*axially* oriented H-10. The characteristic signals of the methyl groups of a *neo*-clerodane skeleton were observed at δ_H_ 0.89 (d, *J* = 6.8 Hz), 1.12 (s), and 0.84 (s) and ascribed to CH_3_-17, CH_3_-19, and CH_3_-20, respectively. The ^13^C NMR data (Table 1) agree with the proposed structure for compound **2**. All NMR assignments for diterpenoid **2** were confirmed with the aid of HMBC (Figure 5) and HSQC spectra.

The relative configuration depicted in **2** was established with the aid of a NOESY spectrum (Figure 5), since cross peaks of correlation were observed between the β-*axially* oriented H-10 and H-6, indicating cofacial orientations for these protons. On the other hand, the signal for H-6 showed NOE with multiple signals at *δ*_H_ 1.73, ascribed to H-8, also β-*axially* oriented.

**Figure 6:**
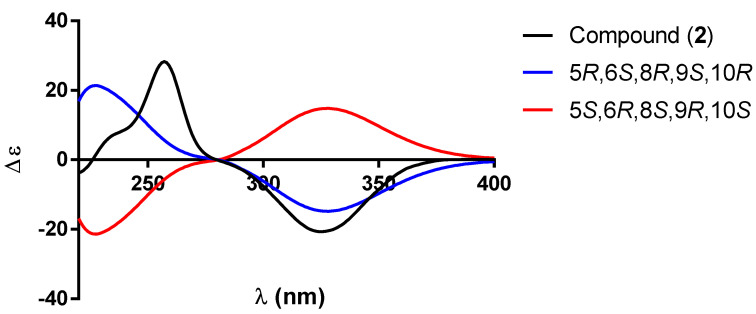
Comparison between the experimental (black line) and calculated ECD spectra for enantiomers (5*R*,6*S*,8*R*,9*S*,10*R*)-**2** (blue line) and (5*S*,6*R*,8*S*,9*R*,10*S*)-**2** (red line).

Based on the previous discussion, the structure of compound **2** was established as the oxidation product of the secondary OH group at C-2 of guevarain A (**1**) and named guevarain B (**2**). The absolute configuration of guevarain B was established via a comparison of the experimental ECD spectrum and the enantiomeric calculated spectra [11]. Figure 6 indicates that the absolute configuration of this diterpenoid corresponds to the 5*R*, 6*S*, 8*R*, 9*S*, 10*R* enantiomer.

The treatment of guevarain A (**1**) with pyridinium chlorochromate (PCC) in CH_2_Cl_2_ afforded a mixture of two reaction products. The main product (44.7% yield) was identical in all aspects to guevarain B (**2**), thus confirming the proposed structure for compound **2**. Structure **1a** was assigned to the minor product (21.8% yield) based on its spectroscopic properties. The ^1^H NMR spectrum of **1a** (Table 2) lacked the signals for the geminal protons of the hydroxyl groups at C-2 and C-6 observed in guevarain A (**1**), while the ^13^C NMR spectrum (Table 2) displayed two carbonyls due to the C-2 and C-6 ketone groups at *δ*_C_ 197.8 and 210.7, respectively, thus confirming oxidation at the C-2 and C-6 positions.

The structure of compound **3** was deduced mainly from the ^1^H NMR spectrum and MS. The molecular formula C_20_H_30_O_5_ was established via HR-DART-MS, indicating an index of hydrogen deficiency Ω = 6. The three degrees of unsaturation can be readily explained by the presence of the butenolide moiety at C-12.

**Figure 7:**
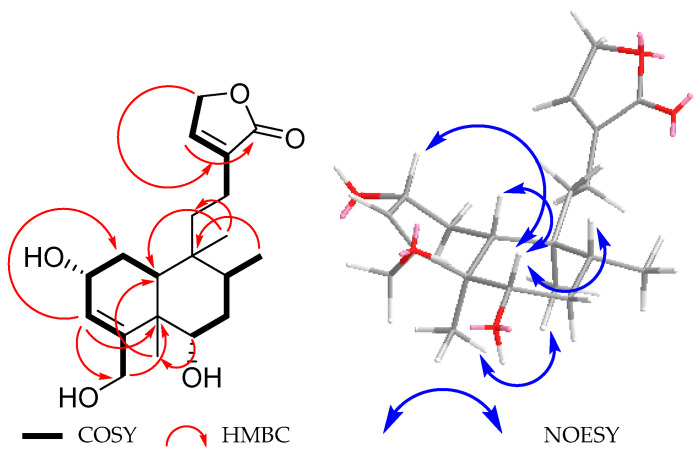
Selected COSY, HMBC, and NOESY correlations for **3**.

The ^1^H NMR of compound **3** (Table 2) was like that of compound **1**, with the main differences being the lack of a vinylic methyl signal and the presence of two AB doublets at δ_H_ 4.08 and 4.23 (*J* = 13.5 Hz), coupled with the vinylic hydrogen broad singlet at δ_H_ 5.56 (H-3), which in turn was found to be coupled with overlapping multiple signals at δ_H_ 4.23 (H-2), in accordance with the COSY spectrum (Figure 7). These facts, in addition to their chemical shifts, led us to ascribe these signals to the C-18 methylene protons and the geminal hydrogen to the hydroxyl group at C-2. The H-2 signal, in turn, showed cross peaks of correlation with two methylene protons at δ_H_ 2.02 (ddt, *J* = 12.5, 6.8, 1.3 Hz) and 1.57 (td, *J* = 12.5, 10.3 Hz) due to H-1 *equatorial* and H-1 *axial*, respectively. A β-*axially* oriented H-10 appeared at δ_H_ 1.40 (brd, *J* = 10.3 Hz) and the hydrogen atom geminal to the hydroxyl group at C-6 appeared at δ_H_ 3.58 as multiple signals similarly to that in guevarain A (**1**).

**Figure 8:**
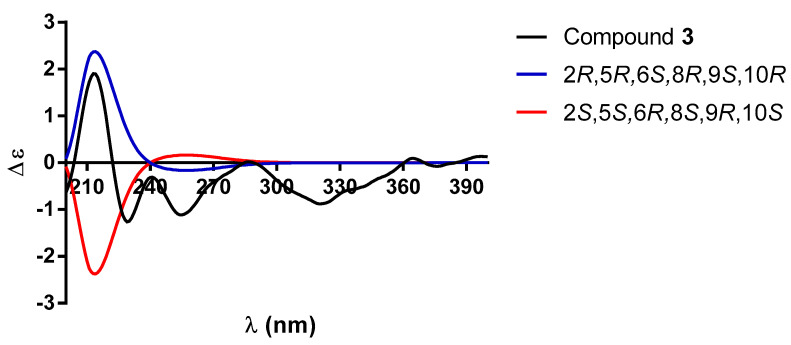
Comparison between the experimental (black line) and calculated ECD spectra for enantiomers (2*R*, 5*R*, 6*S*, 8*R*, 9*S*, 10*R*)-**3** (blue line) and (2*S*, 5*S*, 6*R*, 8*S*, 9*R*, 10*S*)-**3** (red line).

The relative configuration of **3** was established based on NOE interactions (Figure 7). In the NOESY spectrum, the β-*axially* oriented H-10 signal showed cross peaks of correlation with H-2 and H-6, while H-6 showed correlation with H-8, indicating that H-10, H-2, H-6, and H-8 were cofacial. On the other hand, the C-19 methyl hydrogens exhibited NOE with the C-20 methyl group, thus indicating that they were on the same face of the molecule. Based on all the above data, the structure of compound **3** was established as the 18-hydroxy derivative of compound **1**, a structure closely related to that of patagonol (**14**); thus, it was named 2α,6α-dihydroxy-patagonol. The absolute configuration of **3** was established as 2*R*, 5*R*, 6*S*, 8*R*, 9*S*, and 10*R* based on the comparison between the theoretical (blue and red lines) and experimental (black line, Figure 8) ECD curves.

**Figure 9:**
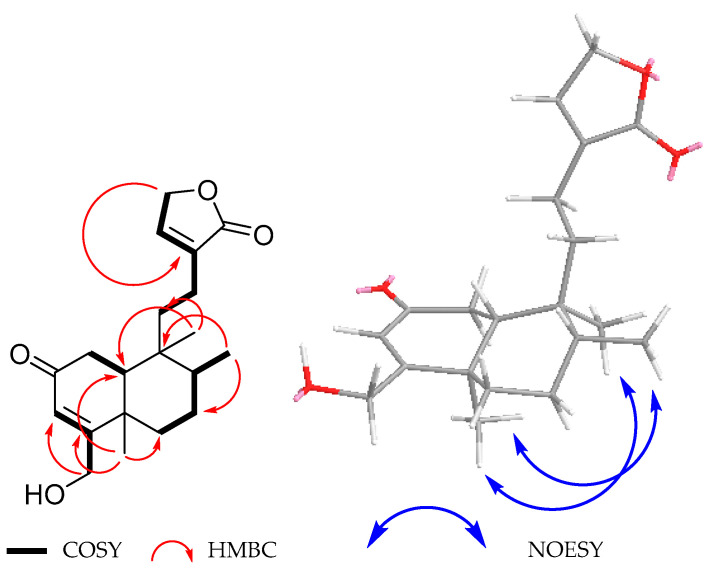
Selected COSY, HMBC, and NOESY correlations for **4**.

The structure of diterpenoid **4** was closely related to that of compound **3**, mainly deduced from its NMR data. The ^1^H NMR spectrum (Table 3) displayed a broad singlet at δ_H_ 6.10 due to the vinylic H-3, which showed cross peaks of correlation in the COSY spectrum (Figure 9) with the C-18 allylic methylene signals at δ_H_ 4.40 (dd, *J* = 17.6, 1.8 Hz) and 4.35 (dd, *J* = 17.6, 1.8 Hz). An ABC system was observed at δ_H_ 2.44 (dd, *J* = 17.8, 13.5 Hz), 2.37 (dd, *J* = 17.8, 4.3 Hz), and 1.98 (dd, *J* = 13.5, 4.3 Hz), while the AB part of this system corresponds to the hydrogen atoms of the CH_2_-1, and the C part is ascribed to the β-*axial* H-10. The COSY spectrum of **4** (Figure 9) supports these assignments since the expected cross peaks of correlation were observed. In the ^1^H NMR spectrum of **4**, despite being similar to that of compound **3**, the lack of an additional hydrogen atom geminal to a hydroxyl function indicated that **4** was devoid of the secondary hydroxyl group at C-6 present in **3**.

**Figure 10:**
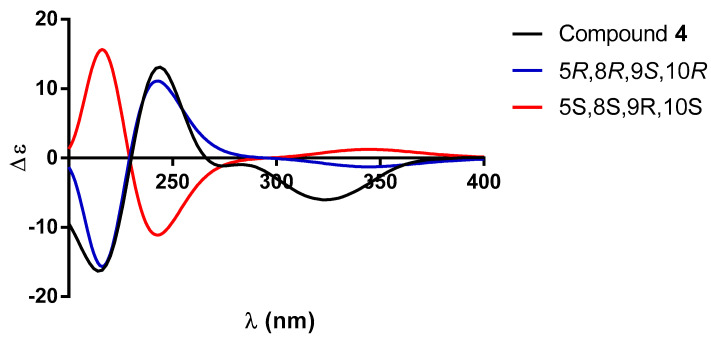
Comparison between the experimental (black line) and calculated ECD spectra for enantiomers (5*R*, 8*R*, 9*S*, 10*R*)-**4** (blue line) and (5*S*, 8*S*, 9*R*, 10*S*)-**4** (red line).

On the other hand, the ^13^C NMR spectrum (Table 3) indicated the presence of three methyl, seven methylene (including two bonded to oxygen), and four methine (two of them of olefinic nature) groups and six fully substituted carbons (including two carbonyls, two *sp*^2^ olefins, and two fully substituted *sp*^3^ carbons). The UV spectrum exhibited two absorption maxima at λ_max_ 218 nm (log ε = 3.65) and 235.4 nm (log ε = 3.52), indicating that in addition to the α,β-unsaturated-γ-lactone, an α,β-unsaturated ketone must be present in compound **4**, as in the case of **2**. The carbon signals at δ_C_ 200.0, 121.4, and 173.6 were assigned to C-2, C-3, and C-4 of this chromophore. These facts led us to establish the structure of compound **4** as 2-oxo-patagonol, as depicted in Figure 1. The absolute configuration of **4** was established as 5*R*, 8*R*, 9*S,* and 10*R* via a comparison between the theoretical and experimental ECD data (Figure 10).

**Figure 11:**
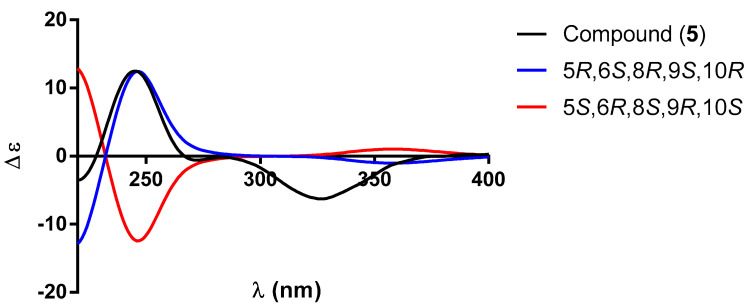
Comparison between the experimental (black line) and calculated ECD spectra for enantiomers (5*R*, 6*S*, 8*R*, 9*S*, 10*R*)-**5** (blue line) and (5*S*, 6*R*, 8*S*, 9*R*, 10*S*)-**5** (red line).

Diterpenoid **5**, C_20_H_28_O_5_ (HR-DART-MS), had similar IR and NMR spectral data to those of **4**, except for the presence of multiple signals at *δ*_H_ 3.83 in the ^1^H NMR spectrum of **5** (Table 3), ascribed to the hydrogen atom geminal to the hydroxyl group attached to C-6. The ^13^C NMR spectrum of **5** supports the presence of this oxygenated function since a signal was observed at *δ*_C_ 73.8, ascribed to C-6. In agreement with the presence of a ketone carbonyl at C-2, the signals of an ABC system were observed in the ^1^H NMR spectrum at *δ*_H_ 2.54 (dd, *J* = 17.8, 13.9 Hz), 2.37 (dd, *J* = 17.8, 3.4 Hz), and 1.91 (dd, *J* = 13.9, 3.6 Hz) (Table 3). The AB part was ascribed to the hydrogen atoms of the C-1 methylene and the C part to the β-*axial* H-10. The rest of the spectrum was similar to that obtained for 2-oxo-patagonol (**4**). As in the case of the previously described diterpenoids **2**–**4**, the absolute configuration of **5** was established via ECD spectroscopy. Figure 11 shows a comparison between the experimental and theoretical ECD spectra that led us to establish that the absolute configuration of **5** was 5*R*, 6*S*, 8*R*, 9*S*, and 10*R*, as depicted. Based on all the above data, compound **5** was identified as a patagonol (**14**) derivative named 2-oxo-6α-hydroxy-patagonol.

**Figure 12:**
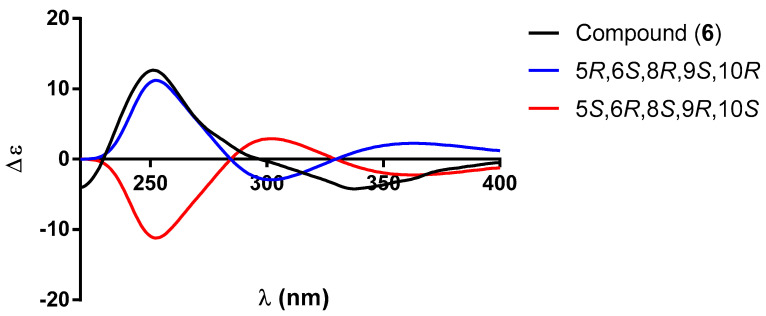
Comparison between the experimental (black line) and calculated ECD spectra for enantiomers (5*R*, 6*S*, 8*R*, 9*S*, 10*R*)−**6** (blue line) and (5*S*, 6*R*, 8*S*, 9*R*, 10*S*)−**6** (red line).

Compound **6** was isolated as a white powder at mp 48−50 °C. The UV spectrum showed two absorption maxima at λ_max_ 217 (log ε = 3.93) and 233 nm (log ε 3.86), thus indicating the presence of chromophores similar to those present in compounds **4** and **5**. The IR spectrum of compound **6** was similar to those of 2-oxo-patagonol (**4**) and 2-oxo-6α-hydroxy-patagonol (**5**), although the intensity of the band at 1676 cm^−1^ ascribed to the α,β-unsaturated ketone was higher, indicating the presence of an additional conjugated carbonyl group. This carbonyl group was part of an aldehyde moiety, since in the ^13^C NMR spectrum of **6** (Table 4), a signal at *δ*_C_ 197.5 was observed, which correlated in the HSQC spectrum with a signal at *δ*_H_ 9.75 (Table 4). These facts indicated that the diterpenoid **6** was closely related to patagonal (**15**), a diterpenoid isolated together with patagonol (**14**) from the *Casimirella* species [10]. Another relevant signal observed in the ^1^H NMR spectrum of **6** was a doublet of doublets at *δ*_H_ 3.75 (*J* = 11.1, 4.7 Hz), which was ascribed to the hydrogen atom geminal to a secondary hydroxyl group located at C-6. The coupling constants, *J* = 11.1, 4.7 Hz, indicated that the hydroxyl was *equatorially* oriented. Based on the previous discussion, the diterpenoid **6** was identified as 6α-hydroxy-patagonal. The absolute configuration of 6α-hydroxy-patagonal (**6**) was also established via ECD. Figure 12 shows a comparison between the experimental and calculated ECD spectra that led us to the conclusion that compound **6** possessed the same absolute configuration as diterpenoids **1**–**5**.

The treatment of 2α,6α-dihydroxy-patagonol (**3**) with PCC afforded, after 24 h of stirring at room temperature, a mixture of compounds identical in all aspects to **5** (10.9% yield) and **6** (31.4% yield), thus confirming the proposed structure for these diterpenoids.

Compounds **7**–**9** exhibited similar ^1^H and ^13^C NMR spectra to those of **3** and **5** (see the experimental section). In addition to the signals that indicated their *neo*-clerodane nature, the ^13^C NMR spectra of **7**–**9** displayed signals of quaternary carbons at *δ*_C_ 101.1, 101.3, and 101.6, respectively. These signals, in addition to the signals of two additional methyl groups, in the range of 23.9–24.9, indicated the presence of an acetonide group in compounds **7**–**9**. Their ^1^H NMR spectra support the presence of the acetonide group since, besides the characteristic methyl groups of a *neo*-clerodane-type diterpenoid, the signals of two additional methyl groups at *δ*_H_ 1.34–1.37 were observed. The HMBC spectra of **7**–**9** showed correlation cross peaks between the C-18 methylene hydrogens (*δ*_H_ 3.65–4.59) and H-6 (*δ*_H_ 3.58–3.75), with the quaternary carbons (*δ*_C_ 100.1–100.6) indicating that the acetonide was formed between the OH groups at the C-18 and C-6 positions. Acetonides **7**–**9** must have formed during the chromatographic purification since acetone was used as an eluent. An inspection of the Dreiding models of compounds **7**–**9** and the precursor diols indicated that the formation of the acetonide is highly favored. That the plausible precursor diols for acetonides **8** and **9** are 2α,6α-dihydroxy-patagonol (**3**) and 2-oxo-6α-hydroxy-patagonol (**5**) was proven via the treatment of 2α,6α-dihydroxy-patagonol (**3**) with anhydrous acetone in the presence of anhydrous CuSO_4_, yielding **8**, and further oxidation of **8** with PCC in dichloromethane, affording compound **9**. While the diols **3** and **5** were obtained from the DCM extract of *S. guevarae*, the precursor of **7** (6α-hydroxy-patagonol) was not isolated; thus, we considered that it was present in the crude extract in small quantities. Based on the previous discussion, acetonides **7**–**9** should be considered acetone artifacts, instead of natural products.

**Figure 13:**
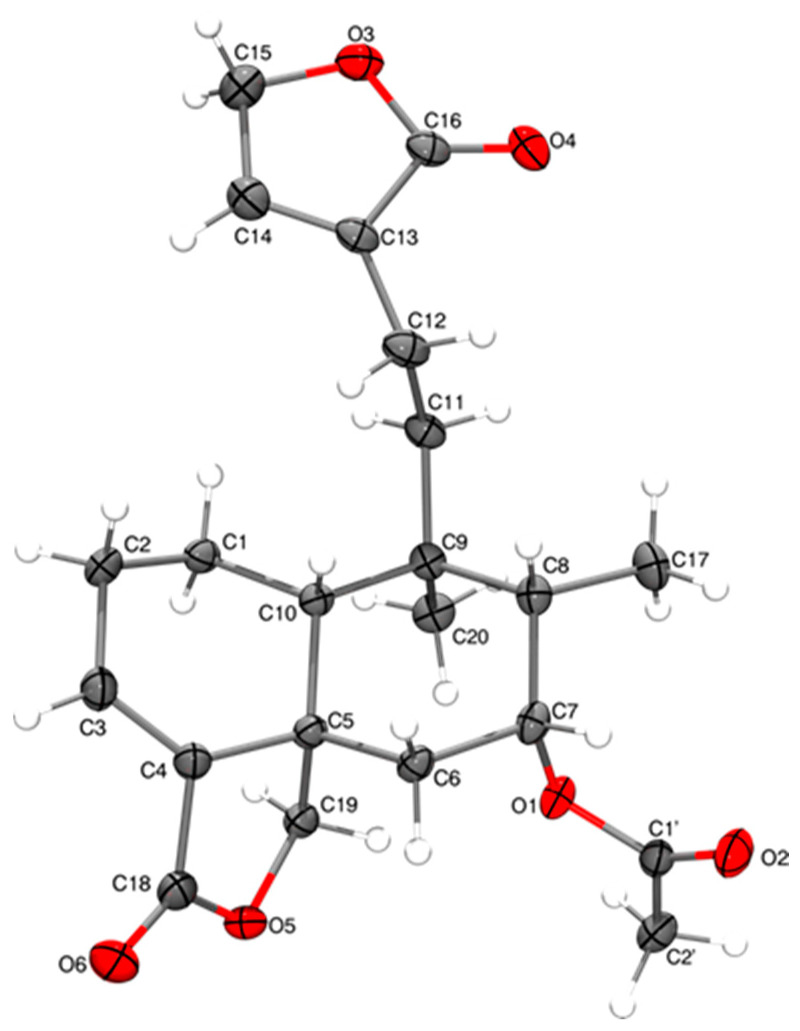
The molecular structure of compound **10**. The thermal ellipsoids are set at 50% of the probability level.

Compound **10** was isolated as colorless crystals at mp 182-184 °C and identified as 7α-acetoxy-*ent*-clerodan-3,13-dien-18,19:16,15-diolide based mainly on its NMR data. Diterpenoid **10** was previously isolated from a population of *Salvia melissodora* Lag. [12] collected in the State of San Luis Potosí, Mexico. The undescribed high-resolution NMR and DART-MS data are recorded in the experimental section. On the other hand, in this study, we report the X-ray analysis of **10** since the absolute configuration of this diterpenoid was not established in its original description. The molecular structure of **10** is shown in Figure 13, and the crystallographic details are presented in the Supplementary Materials (Table S1). Compound **10** was crystallized in the orthorhombic P2(1)2(1)2(1) space group, and its absolute structure parameters—Flack(x), 0.01(5); Hooft(y), 0.00(5); and Parsons(z), 0.00(3)—confirm the assignment of the crystal model as well as the configuration of the chiral carbon atoms as being 5*S*, 7*R*, 8*S*, 9*R*, and 10*R*.

While products **11** and **12** have previously been obtained from *S. melissodora* and identified as 7-keto-*ent*-clerodan-3,13-dien-18,19:16,15-diolide and 7α-hydroxy-*ent*-clerodan-3,13-dien-18,19:16,15-diolide, respectively [12], compound **13** was identical in all aspect to mkapwanin, previously isolated from *Dodonea angustifolia* (Sapindaceae) [13]. The absolute configuration of these diterpenoids, as depicted in **11**–**13**, was established via a comparison of their experimental ECD spectra with theoretically calculated curves (see Supplemental Figure S61 and Figure S62, and S63). The undescribed high-resolution NMR and DART-MS data are recorded in the experimental section.

In addition to the previously described *neo*-clerodane diterpenoids, six already known triterpenoids were isolated from the DCM extract of *S. guevarae*, identified as 3β,20,25-trihydroxylupane [14], oleanolic acid [15], 3β-*O*-acetyl-oleanolic acid [16], ursolic acid [17], 3β-*O*-acetyl-betulinic acid [18], and 3β,28-*O*-diacetyl-betulin [19].

Chromatographic purification of the methanol extract of *S. guevarae* afforded several previously described phenolic compounds identified via spectroscopic and spectrometric methods and a comparison with literature data: quercetin-3-*O*-β-xylopyranosil-(1 → 2)-β-galactopyranoside, taxifolin-7-*O*-β-glucopyranoside, naringenin 7-*O*-β-glucopyranoside, a mixture of 2*R* and 2*S* eriodictyol-7-*O*-β-glucopyranoside, caffeic acid, the methyl ester of rosmarinic acid, and rosmarinic acid. Quercetin-3-*O*-β-xylopyranosil-(1 → 2)-β-galactopyranoside was originally isolated from the horseradish *Amoracia rusticana* (Brassicaceae) [20]; white clover *Trifolium repens* (Fabaceae) [21]; *Asclepias syriaca* (Asclepidaceae) [22]; *Prunus padus* (Rosaceae) [23]; and recently, *Elaeagnus umbellata* (Elaeagnaceae) [24]. Taxifolin-7*-O*-β-glucopyranoside was isolated for the first time from *Podocarpus nivalis* (Podocarpaceae) [25] and from species belonging to different plant families including Pinaceae [26,27,28], Rosaceae, and Lamiaceae [29]. While the herbal drugs *Crataegus pentagyna* (Rosaceae) (*Crataegi folium cum flore*) [30] and *Lysimachia patungensis* (Primulaceae) are natural sources of naringenin 7-*O*-β-glucopyranoside, a mixture of diastereomers (2*R*) and (2*S*)-eriodictyol 7-*O*-β-D-glucopyranoside was isolated from the parasitic plant *Balanophora involucrata* (Balanophoraceae) [31]. Caffeic acid is a widely distributed compound and has been reported in several *Salvia spp.* [32,33,34]. Rosmarinic acid is an ester of caffeic acid and 3,4-dihydroxyphenyllactic acid, commonly found in members of the Lamiaceae family, and exhibits a wide range of biological activities, such as antioxidant, antibacterial, antiviral, and anti-inflammatory activities [35]. The methyl ester of rosmarinic acid is also widely distributed in genera of the Lamiaceae family such as *Salvia* and *Melissa* [36].

### 2.2. Biological Activity

#### 2.2.1. Antiproliferative Activity

The Lamiaceae and Euphorbiaceae families are a rich source of diterpenoids with promising anticancer activity. In the Lamiaceae family, although several abietane- and icetexane-type diterpenoids exhibited interesting antiproliferative activity in different cancer cell lines, some *trans*-clerodane diterpenoids also have demonstrated interesting cytotoxic activity [37]. Therefore, we evaluated the antiproliferative activity of some of the diterpenoids isolated from *S. guevarae* against a panel of six human cancer cell lines: U251 (glioblastoma), PC-3 (prostate cancer), K562 (chronic myelogenous leukemia), HCT-15 (colon cancer), MCF-7 (breast cancer), and SKLU-1 (lung adenocarcinoma). The cell line COS-7 (monkey kidney) was used for an evaluation of the cytotoxicity in a normal cell line. The results of the primary screening of the crude DCM extract at 25 ppm and diterpenoids **1**–**3**,**1a**, **3**, and **5**–**11** at 25 µM are included in Table S3. Significant inhibition of the K562 cell line was observed for the crude DCM extract, with 96.6%, and no significant cytotoxicity was observed in the other cancer cell lines of the panel. Guevarain B (**2**) and the acetonide of 6α-hydroxy-patagonol (**7**) showed only moderate, although selective, activity against K562, with 43.3 and 46.9% inhibition, respectively, being non-cytotoxic to normal cell line COS-7 and the rest of the panel. Weak cytotoxic activity against K562 was observed for 6α-hydroxy-patagonal (**6**) (34.7%) and compound **10** (31.7%). Compound **1a** and 6α-hydroxy-patagonal (**6**) also exhibited weak activity against U251 (35.2%) and PC-3 (38.1%), respectively. Only diterpenoid **10** proved to be weakly cytotoxic against COS-7 (30.6%). Table 5 shows the results of the IC*_50_* of guevarain B (**2**) and the acetonide of 6α-hydroxy-patagonol (**7**) against the chronic myelogenous leukemia K562 cell line compared with that of Adriamycin.

#### 2.2.2. Anti-Inflammatory Activity Due to the Inhibition of NO Production in RAW 264.7 Macrophage Cells

Terpenoids have been widely studied due to their great structural diversity and the wide range of biological activities they have exhibited, among which an anti-inflammatory effect can be listed. Diterpenes belonging to different molecular arrangements, including labdane-, kaurane-, pimarane-, and clerodane-type diterpenoids, have been shown to have interesting anti-inflammatory effects in different experimental models, among them the inhibition of NO release in lipopolysaccharide-stimulated RAW 264.7 macrophage cells [38]. Based on these backgrounds, the evaluation of the anti-inflammatory effect of some of the diterpenes obtained from *S. guevarae* was carried out through the quantification of the release of NO via nitrite formation, through the Griess reaction, in stimulated RAW 264.7 macrophages. The induction of NO was promoted using *E. coli* lipopolysaccharide at 1 µg/mL (*E. coli*, serotype 055 B5, Sigma). Table S4 shows the results obtained for diterpenoids **1**–**3**, **5**–**7**, and **9**–**10**. Aminoguanidine and celecoxib, at a concentration of 100 µM, were used as standards. Cell viability was evaluated via the MTT method. It is important to mention that none of the evaluated diterpenes generated nitrites in the supernatant, which indicates that they do not induce the formation of NO. Compound **5** showed moderate inhibition; however, a higher percentage of activity was observed for compounds **6**, **7**, and **10**. Table 6 shows the IC_50_ value for the most active compounds compared with that of aminoguanidine. 6α-Hydroxy-patagonal (**6**) showed an activity comparable with that of the standard; however, a 15% decrease in the viability of macrophages was observed (data shown in Table S4). On the other hand, compounds **7** and **10** showed an IC_50_ lower than that observed for aminoguanidine, with high viability in the RAW 264.7 cells. These results indicate that products **6**, **7**, and **10** have promising anti-inflammatory activity.

### 2.3. Taxonomic Considerations

**Figure 14:**
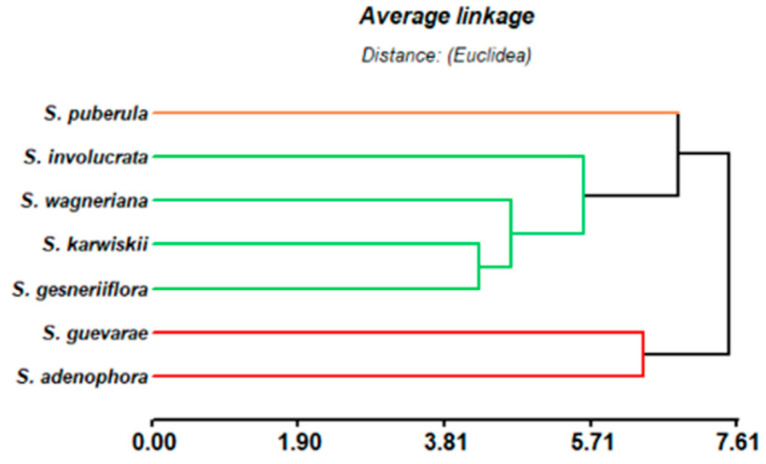
Hierarchical cluster analysis of the *Salvia* species from section *Holwaya* with phytochemicals studies, including *S. guevarae*.

In its original description, *S. guevarae* was classified in section Holwaya Ramamoorthy [9], together with the Mexican species *S. holwayi* Blake, *S. karwinskii* Benth., *S. involucrata* Cav., *S. puberula* Fernald, *S. stolonifera* Benth., and *S. wagneriana* Polak [8]. On the other hand, in 1991, during a taxonomic revision of section Nobiles Epling, *S. adenophora* Fernald, *S. disjuncta* Fernald, and *S. gesneriflora* Lindl & Paxton were transferred to section Holwaya [39]. Although from the taxonomic point of view, *S. guevarae* is closely related to *S. karwinskii*, *S. wagneriana*, *S. involucrata*, and *S. holwayii*, a hierarchical cluster analysis of the diterpenoid content of the members of section Holwaya phytochemically studied thus far, such as *S. adenophora* [40], *S. gesneriflora* [41,42], *S. involucrata* [43], *S. puberula* [44], *S. karwinskii* [45], and *S. wagneriana* [46], including the results obtained in this work from *S. guevarae*, indicated the presence of three groups that can be distinguished (Figure 14). *S. puberula* is, from the chemosystematic point of view, different from the rest of the species in section Holwaya, since *S. puberula* contents only rearranged isosalvipuberulane and salvipuberulane diterpenoids. On the other hand, *S. wagneriana*, *S. karwinskii*, *S. involucrata*, and *S. gesneriflora* constitute a second group with similar diterpenoid constituents. The third group indicates a close similarity between the diterpenoids obtained from *S. adenophora* and *S. guevarae*. Although no phylogenetical analysis has been conducted on *S. guevarae*, the oxidation and substitution pattern of the *neo*-clerodane described in this work support the inclusion of *S. guevarae* in section Holwaya.

## 3. Materials and Methods

### 3.1. General Experimental Procedures

The melting points (uncorrected) were determined on a Fisher Johns apparatus (Fisher Scientific Company, Pittsburgh, PA, USA). Optical rotations were measured on a PerkinElmer 341 polarimeter (PerkinElmer Inc., London, UK). The UV spectra were recorded on a Shimadzu UV 160U spectrophotometer (Shimadzu, Kyoto, Japan). The ECD spectra were measured using a JASCO J-1500 Circular Dichroism Spectrophotometer (JASCO Inc., Easton, MD, USA). The IR spectra were obtained on a Bruker Tensor 27 spectrometer (Bruker Corporation, Billerica, MA, USA); 1D and 2D NMR experiments were performed on a Bruker Advance III HD spectrometer (Bruker Corporation, Billerica, MA, USA) at 700 MHz for ^1^H and 175 MHz for ^13^C, a Bruker AVANCE III HD spectrometer (Bruker Corporation, Billerica, MA, USA) at 500 MHz for ^1^H and 125 MHz for ^13^C, or a Bruker Avance III (Bruker Corporation, Billerica, MA, USA) at 400 MHz for ^1^H and 100 MHz for ^13^C. CDCl_3_, (CD_3_)_2_CO, and CD_3_OD were used as solvents as indicated, and the chemical shifts were attributed to the residual solvents CHCl_3_ (*δ*_H_ = 7.26, *δ*_C_ = 77.16), CH_3_OH (*δ*_H_ = 3.31, *δ*_C_ = 49), and (CH_3_)_2_CO (*δ*_H_ = 2.05, *δ*_C_ = 29.84). The HR-DART-MS data were obtained on The AccuTOF JMS-T100LC mass spectrometer created by Jeol (Jeol Ltd., Tokyo, Japan) or the HPLC-ESI-QTOF-MS 1260-G6530 model (Agilent Tech. Santa Clara, CA, USA) using a column Zorbax Extend-C18 (Agilent, 50 × 3.0 mm × 3.5 μm), with the mobile phase as follows: A, H_2_O with CH_3_COOH 0.1% and B, CH_3_CN (in a gradient (0–12 min (85% A, 15% B): 100% B), 0.400 mL/min 600.00 bar). The X-ray data were collected on a Bruker APEX II DUO diffractometer (Bruker Corporation, Billerica, MA, USA). Silica gel 230–400 mesh (Macherey-Nagel; Düren, Germany) and Sephadex LH-20 (Pharmacia Biotech AB, Uppsala, Sweden) were used for Column Chromatography (CC). Precoated silica gel TLC plates (Merck, St. Louis, MO, USA) were used for thin layer chromatography (TLC).

### 3.2. Plant Materials

*Salvia guevarae* was collected at Xilitla, San Luis Potosi, Mexico, 21°24′34″ N, −99°5′9″ W, and 2100 m altitude, in October 2018. The plant material was identified by Dr. Brenda Y. Bedolla-Garcia and Dr. Sergio Zamudio and deposited in the Herbarium of the Instituto de Ecología, A. C., Centro Regional del Bajío (voucher IEB-266884).

### 3.3. Extraction and Isolation

The dried and powdered leaves of *S. guevarae* (361 g) were extracted via percolation with CH_2_Cl_2_ (2 L). The CH_2_Cl_2_ extract was concentrated at reduced pressures to yield 10.5 g of residue, a portion (9.5 g) of which was subjected to column chromatography (CC, 21 cm in length by 5 cm in internal diameter) on silica gel using a gradient elution with C_6_H_14_/CH_3_COOCH_2_CH_3_ (100:0-0:100); (CH_3_)_2_CO (100%); and finally, CH_3_OH as the mobile phase to obtain 80 eluates, 150 mL each, which were combined into 22 major fractions (A−U) after thin-layer chromatography (TLC) evaluation. The acetylation of fraction H (50 mg) and subsequent purification using preparative TLC on silica gel, eluting with C_6_H_14_/CH_3_COOCH_2_CH_3_ (80:20) as the mobile phase, gave 3β,28-*O*-diacetyl betulin (13.2 mg) and a mixture of 3β-*O*-acetyl oleanolic acid and 3β*-O*-acetyl betulinic acid (18.9 mg). Oleanolic and ursolic acids were identified as the constituents of fraction I (1296 mg). Fraction M (277 mg) was subjected to CC (35 × 2.5 cm) on silica gel, eluting with C_6_H_14_/CH_3_COOCH_2_CH_3_ (40:60) to obtain 25 subfractions, 50 mL each, which were combined into 8 major subfractions (MA−MH) based on a TLC evaluation. Subfraction MC (48 mg) was purified via preparative TLC on silica gel, eluting with CH_2_Cl_2_/CH_3_COOCH_2_CH_3_ (70:30) to give **13** (1.7 mg). Compound **10** (13.7 mg) was obtained as a solid from fraction N, and the mother liquors (369 mg) of this fraction were subjected to CC (20 × 35cm) on silica gel using C_6_H_14_/(CH_3_)_2_CO (80:20) as the eluent to obtain 14 subfractions (NA−NN) based on a TLC profile. Compound **7** (11 mg) was identified from fraction NC. Compound **10** (116 mg) was crystallized from C_6_H_14_/CH_3_COOCH_2_CH_3_ in fraction NJ. Subfraction NK (28.5 mg) was subjected to preparative TLC on silica gel, eluting with CH_2_Cl_2_/CH_3_COOCH_2_CH_3_ (8:2) to give **12** (2 mg). Crystals of compound **1** (711 mg) were obtained from fraction O, and the mother liquor’s residue (1079 mg) was subjected to CC on silica gel using CH_2_Cl_2_/(CH_3_)_2_CO (90:10) to obtain 18 subfractions (OA−OQ) based on a TLC evaluation. Subfraction OF (156 mg) was subjected to CC (32.5 × 2 cm) on Sephadex LH-20 using CH_3_OH as an eluent to obtain four subfractions (OFA−OFD) based on a TLC evaluation. Subfraction OFC (29 mg) was subjected to preparative TLC on silica gel, eluting with CH_2_Cl_2_/CH_3_COOCH_2_CH_3_ (70:30) to give **11** (8 mg). Subfraction OI (55 mg) was purified via TLC on silica gel in the C8 reverse phase (RP-8) using CH_3_OH/H_2_O (70:30) as the mobile phase to give **2** (30 mg). Compound 3β,20,25-trihydroxylupane (18 mg) was identified as the constituent of fraction ON. Fraction P (398 mg) was subjected to CC (15 × 1.8 cm) on Sephadex LH-20, eluting with methanol to obtain six subfractions (PA−PF) based on a TLC evaluation. Subfraction PC (261 mg) was subjected to CC (12 × 2 cm) on silica gel using C_6_H_14_/(CH_3_)_2_CO (50:50) to obtain 12 subfractions (PCA−PCL) based on a TLC evaluation. Compound **4** (3 mg) was purified from subfraction PCF via TLC on silica gel, eluting with C_6_H_14_/CH_3_COOCH_2_CH_3_/CH_3_OH/H_2_O (100:100:8:3.5). Compound **3** (410 mg) was obtained as a powder from fraction Q. Subfraction R (717 mg) was subjected to CC (22 × 2.5 cm) on silica gel using CH_2_Cl_2_/(CH_3_)_2_CO (60:40) to obtain 13 subfractions (RA−RM) after TLC evaluation. Subfraction RC (48 mg) was purified via preparative TLC using C_6_H_14/_C_7_H_8_/CH_3_COOCH_2_CH_3_ (30:30:40) as the mobile phase to obtain compound **6** (1.8 mg). Subfraction RD (146 mg) was purified via CC (11 × 1.8 cm) on silica gel using C_6_H_14_/CH_3_COOCH_2_CH_3_ (70:30) as the mobile phase to obtain 9 subfractions (RDA−RDI) based on a TLC evaluation. Compound **8** (61.8 mg) was obtained from subfraction RDE. Subfraction RF (100 mg) was purified via CC (11 × 2.5 cm) on silica gel using C_6_H_14_/CH_3_COOCH_2_CH_3_ (30:70) as an eluent to obtain 7 subfractions (RFA−RFG) based on a TLC profile. Compound **5** (4 mg) was obtained from subfraction RFD. Subfraction RFE (54 mg) was treated with anhydrous acetone (2 mL) and copper sulfate (120 mg) to obtain a mix of compounds. Compound **9** (2 mg) was obtained after purification via TLC on silica gel, eluting with CH_2_Cl_2_/(CH_3_)_2_CO (70:30) as the mobile phase.

The material previously extracted with dichloromethane was subjected to percolation with methanol to obtain 12.8 g of an extract. The methanolic extract was subjected to a liquid–liquid extraction process with a mixture of (C_6_H_14_:C_6_H_6_)/(CH_3_OH:H_2_O) (25:25:45:5), and the CH_3_OH:H_2_O fraction (11.4 g) was fractionated on a Sephadex LH 20 column using methanol as the mobile phase to obtain 7 fractions (A–G). Subfraction C (600 mg) was subjected to flash chromatography (14 × 5 cm) in the C_18_ reverse phase (RP-18), eluting with a mixture of CH_3_CN:H_2_O (0.1% formic acid) (5:100-100:0) to obtain 20 subfractions (CA-CT) based on an analytical TLC evaluation. Taxifolin-7-*O*-β-glucopyranoside (45 mg) was obtained from subfraction CF. Subfraction CI (35 mg) was purified via TLC on RP-18 using CH_3_COOCH_2_CH_3_/CH_3_OH (70:30) to give quercetin-3-*O*-β-xylopyranosyl-(1 → 2)-β-galactopyranoside (12 mg) and caffeic acid (2.5 mg). A mixture of 2*R* and 2*S* eriodictyol-7-*O*-β-glucopyranoside (1.8 mg) was obtained from subfraction CJ. Subfraction CN (20 mg) was subjected to TLC on RP-18 using CH_3_OH/H_2_O (40:60) as the mobile phase to give naranginin-7-*O*-β-glucopyranoside (6 mg) and rosmarinic acid (10 mg). 3-*O*-Methylrosmarinic acid was identified from subfraction CR.

**Guevarain A** (**1**): colorless crystals; mp 136−138 °C; [α] ^25^_D_ +23.3 (c 0.0045, MeOH); UV (MeOH) λ_max_ (log ε) 207 (3.16) nm; IR (ATR) ν_max_ 3606, 2964, 2932, 2876, 1755, 1654, 1602, 1451, 1349, 1203, 1076, 998 cm^−1^; ^1^H and ^13^C NMR ((CD_3_)_2_CO): see Table 1; HR-DART-MS *m*/*z* 317.2103 [M + H − H_2_O]^+^; (calcd for C_20_H_29_O_3_, 317.2117).

**Guevarain B** (**2**): colorless crystals; mp 112−114 °C; [α]^25^_D_ −5.6 (c 0.0006, MeOH); UV (MeOH) λ_max_ (log ε) 214.4 (4.08), 239.8 (4.07), 227.8 (4.01) nm; ECD (*c* 3.01 mM, MeOH) [*θ*]_257_ +92349.6, [*θ*]_278_ 0, [*θ*]_325_ −68602.6; IR (ATR) ν_max_ 3417, 2957, 2930, 2869, 1747, 1644, 1607, 826 cm^−1^; ^1^H and ^13^C NMR (CDCl_3_): see Table 1; HR-DART-MS *m*/*z* 333.2063 [M+H]^+^; (calcd for C_20_H_29_O_4_, 333.2066).

**2α,6α-Dihydroxy-patagonol** (**3**): white powder; mp 98−100 °C; [α]^25^_D_ +23.1 (c 0.0016, MeOH); UV (MeOH) λ_max_ (log ε) 207.5 (6.45) nm; ECD (*c* 3.14 mM, MeOH) [*θ*]_213_ +6266.6, [*θ*]_229_ −4287.7, [*θ*]_255_ −5936.8, [*θ*]_321_ −2968.4; IR (ATR) ν_max_ 3266, 1754, 1649, 1642 cm^−1^; ^1^H and ^13^C NMR (CD_3_OD): see Table 2; HR-DART-MS *m*/*z* 350.2090 [M-H]^+^; (calcd for C_20_H_30_O_5_, 349.2093).

**2-Oxo-patagonol** (**4**): white powder; mp 103−105 °C; [α]^25^_D_ −25.6 (c 0.0018, MeOH); UV (MeOH) λ_max_ (log ε) 218.0 (3.65) nm; ECD (*c* 5.41 mM, MeOH) [*θ*]_215_ −53430.8, [*θ*]_244_ +42876.6, [*θ*]_322_ −20448.8; IR (ATR) ν_max_ 3411, 2927, 2872, 1747, 1651, 1050, 773 cm^−1^; ^1^H and ^13^C NMR (CDCl_3_): see Table 3; HR-DART-MS *m*/*z* 333.2066 [M+H]^+^; (calcd for C_20_H_29_O_4_, 333.2066).

**6α-Hydroxy-2-oxo-patagonol** (**5**): white powder; mp 92–94 °C; [α]^25^_D_ +16 (c 0.001, MeOH); UV (MeOH) λ_max_ (log ε) 241.2 (3.85) nm; ECD (*c* 2.87 mM, MeOH) [*θ*]_245_ +40897.7, [*θ*]_284_ 0, [*θ*]_326_ 21108.5; IR (ATR) ν_max_ 3400, 2956, 2929, 2874, 1741, 1650, 1449, 1266, 1209, 1006, 733 cm^−1^; ^1^H and ^13^C NMR (CDCl_3_): see Table 3; HR-DART-MS *m*/*z* 349.2025 [M+H]^+^; (calcd for C_20_H_29_O_5_, 349.2015).

**6α-Hydroxy-2-oxo-patagonal** (**6**): white powder; mp 48−50 °C; [α]^25^_D_ −12.7 (c 0.0011, MeOH); UV (MeOH) λ_max_ (log ε) 241.4 (4.08) nm; ECD (*c* 3.17 mM, MeOH) [*θ*]_251_ +41557.3, [*θ*]_295_ 0, [*θ*]_338_ −14512.1; IR (ATR) ν_max_ 3439, 2958, 2931, 2876, 1744, 1676, 1449, 1348, 1261, 1206, 1072, 1048, 832 cm^−1^; ^1^H and ^13^C NMR (CDCl_3_): see Table 4; HR-DART-MS *m*/*z* 347.1859 [M + H]^+^; (calcd for C_20_H_29_O_4_, 347.1859).

**2,6-Dioxo-guevarain** (**1a**): Treatment of **1** (50 mg) with pyridinium chlorochromate (20.1 mg) in DCM gave a complex mixture, which was subjected to CC in silica gel using EtOAc as the mobile phase to afford guevarain B (**2**) (14.4 mg) and **1a** (7 mg) as a white powder; mp 148−150 °C; [α]^25^_D_ −36 (c 0.003, MeOH); UV (MeOH) λ_max_ (log ε) 241.4 (4.08) nm; IR (ATR) ν_max_ 3416, 1746, 1643, 1607 cm^−1^; ^1^H and ^13^C NMR (CDCl_3_): see Table 2; HR-DART-MS m/z 331.1898 [M + H]^+^; (calcd for C_20_H_27_O_4_, 331.1909).

**6α-Hydroxy-2-oxo-patagonal** (**6**): Treatment of **3** (24.8 mg) with pyridinium chlorochromate (15.25 mg) gave a complex mixture, which was subjected to CC in silica gel using EtOAc as the mobile phase to afford **5** (2.7 mg) and **6** (7.7 mg) as white powders.

**Acetonide 7**. Compound **3** (51.4 mg) was dissolved in freshly distilled acetone and treated with anhydrous CuSO4 (35 mg) and stirred for 1 h. A work-up of the reaction afforded 34.4 mg of a compound, identical in all aspects to **7**.

**Acetonide 9**. The treatment of synthetic diterpenoid **7** (34 mg) was oxidized with 57 mg of PCC in DCM. After stirring at room temperature for 24 h, 26 mg (76%) of a compound identical to acetonide **9** was obtained.

### 3.4. Electronic Circular Dichroism Calculations

The compounds **2**–**6** and **10**–**13** were constructed, and their geometry was optimized using a semiempirical method (PM3), as implemented in Spartan’10. A conformational analysis was performed using the same software and force field. All conformers were filtered and checked for redundancy. Subsequently, the conformers with relativity energies ≤ 2.0 or 2.5 Kcal/mol were minimized and optimized with Gaussian 09 using a DFT force field at the B3YLP/DGZVP level of theory for optimization and frequency. The ECD calculations for compounds **2**–**7** and **12**–**14** in a MeOH solution were carried out by employing a TD-SCF force field at the B3LYP/6-31G(d) theory level, with the default solvent model. The calculated excitation energy (nm) and rotatory strength (R) in dipole velocity (Rvel) form were simulated into an ECD curve using Equation (1), as implemented in SpecDis software (Version 1.71), where E0k and R0k are the transition energy and rotatory strength of kth electronic transition, respectively, and σ is the exponential half width.

(1) $$\Delta \varepsilon =\frac{1}{2.297\times 10^{-39}}\;\times \;\frac{1}{\sigma \sqrt{\pi }}\sum_{k}E_{0k}R_{0k}exp\left[-{\left\{\frac{E-E_{0k}}{\sigma }\right\}}^{2}\right]$$

All calculations were performed on the HP Cluster Platform 3000SL Miztli, a parallel supercomputer with a Linux operating system, containing 25,312 cores and a total of 45,000 GB of RAM.

### 3.5. Single-Crystal X-Ray Diffraction Analysis of 1 and 10

The relevant details of the crystals, data collection, and structure refinement for compounds **1** and **10** can be found in Table S1. The data for guevarain **A** (**1**) and diterpenoid **10** were collected on a Bruker APEX II CCD Diffractometer at 100 K, using Cu-Ka radiation (k = 1.54178 Å) from an Incoatec ImuS source and Helios optic monochromator. Suitable crystals were coated with hydrocarbon oil, picked up with a nylon loop, and mounted in the cold nitrogen stream of the diffractometer. Frames were collected using ω scans and integrated with SAINT multi-scan absorption correction (SADABS). The structures were solved using direct methods and refined via full-matrix least-squares on F2 with SHELXL-2018 [47] using the SHELXLE GUI [48]. The hydrogen atoms of the C–H bonds were placed in idealized positions, the hydrogens of the O–H moiety and the hydrogens of the water molecules were found on a map of residual density, and their position was refined with U_iso_ = aU_eq_ (where a is 1.5 for –CH_3_ and –OH moieties and 1.2 for others). The molecular graphics were prepared using the POV-RAY and GIMP programs. The crystallographic data for the crystal’s structures have been deposited at the Cambridge Crystallographic Data Centre under reference numbers CCDC 2,430,636 and CCDC 2430637 for compounds **1** and **10**, respectively.

The crystal data of **1** were collected from a colorless prism (0.433 × 0.405 × 0.290 mm^3^) at 100(2) K: C_20_H_32_O_5_, MW = 352.45, monoclinic, space group P2_1_, unit cell dimensions a = 8.78990(10) Ǻ, b = 10.45200(10) Ǻ, c = 10.27570(10) Ǻ, α = γ = 90°, β = 90.2546(4)°, V = 944.040(17) Ǻ^3^, Z = 2, Dc = 1.240 Mg/m^3^, F(000) = 384. A total of 33,896 reflections were collected in the range 4.302° < θ < 71.003°, with 3601 independent reflections [R(int) = 0.0304]; completeness to θ_max_ was 99.8%. The structure was solved using direct methods and refined via full-matrix least squares on F^2^, with anisotropic temperature factors for non-hydrogen atoms converging at R final indices [I > 2σ(I)], R1 = 0.0266, wR2 = 0.0725; R indices (all data), R1 = 0.0266, wR2 = 0.0725. The absolute Flack, Hooft, and Parsons structure parameters were −0.02(3), −0.02(2), and −0.019(16), respectively, thus indicating that the absolute configuration of compound **1** is that depicted in Figure 3. The configuration of the chiral carbon atoms is 2*R*, 5*R*, 6*S*, 8*R*, 9*S*, and 10*R*.

Crystallographic data for compound **10** were collected from a colorless prism (0.401 × 0.319 × 0.278 mm^3^) at 100(2) K, orthorhombic, space group P2**_1_**2**_1_**2**_1_** (Table S1). A total of 32,163 reflections were collected, and the structure was solved using direct methods. The absolute Flack, Hooft, and Parsons structure parameters were 0.01(5), 0.00(5), and 0.00(3), thus indicating that the absolute configuration of compound **10** is that depicted in Figure 13.

### 3.6. Chemometric Analysis

A cluster analysis was carried out using a complete chaining method. The data consisted of structural characteristics of 47 diterpenoids reported from the *Salvia* species classified in section *Holwaya*, including *S. guevarae*. The type of diterpenoid scaffolds, the unsaturation index, the oxidation pattern, and the presence of lactones or furan groups in the structure were analyzed. The analysis was carried out using InfoStat software (version 2017, released in 2020).

### 3.7. Antiproliferative Activity

The crude DCM extracts at 25 ppm and diterpenoids **1**–**3**, **1a**, **3**, and **5**–**11** at 25 µM were evaluated in vitro against a panel of six human cancer cell lines: U251 (glioblastoma), PC-3 (prostate cancer), K562 (chronic myelogenous leukemia), HCT-15 (colon cancer), MCF-7 (breast cancer), and SKLU-1 (lung adenocarcinoma). The cell line COS-7 (monkey kidney) was used for the evaluation of the cytotoxicity in a normal cell line. The cell lines were supplied by the National Cancer Institute (USA) and American Type Culture Cells (ATCC). The cytotoxicity was evaluated using the protein-binding dye sulforhodamine B (SRB) in a microculture assay as previously reported by Monks et al. [7,49].

### 3.8. NO Inhibition in RAW 264.7 Macrophages

RAW 264.7 cells were obtained from the American Type Culture Collection (ATCC TIB-71), and cultured as previously described. Diterpenoids **1**–**3**, **5**–**7**, and **9**–**10** were dissolved in DMSO at 25 µM. The nitrite concentration in the medium was quantified as an indirect indicator of NO production following the Griess reaction and the experimental protocol previously reported [50]. Cell viability was evaluated via the MTT method, according to the protocol described by Mosmann and coworkers [51]. Table S4 shows the results obtained for the inhibition in nitrite production, and Table 6 shows the IC_50_ value for the most active compounds compared with those of aminoguanidine.

## 4. Conclusions

From the dichloromethane and methanolic extracts of the leaves of the new species *Salvia guevarae*, 13 *neo*-clerodanes diterpenoids (**1**–**13**) were isolated, of which **1**–**6** have yet to be described in the literature. The three acetonides **7**–**9** were artifacts produced via the reaction of diol derivatives with the acetone used in some parts of the separation process. The *neo*-clerodane diterpenoids **10**–**13** were known diterpenoids previously described from different sources. Six triterpenoids were also isolated and identified: 3β,20,25-trihydroxylupane, oleanolic acid, 3β-*O*-acetyl-oleanolic acid, ursolic acid, 3β-*O*-acetyl-betulinic acid, and 3β,28-*O*-diacetyl-betulin. Additionally, from the methanol extract of *S. guevarae*, several phenolic derivatives were isolated and identified: quercetin-3-*O*-β-xylopyranosyl-(1 → 2)-β-galactopyranoside, taxifolin-7-*O*-β-glucopyranoside, naringenin-7-*O*-β-glucopyranoside, a mixture of 2*R* and 2*S* eriodictyol-7-*O*-β-glucopyranoside, caffeic acid, the methyl ester of rosmarinic acid, and rosmarinic acid. The structure of the isolated compounds was established by spectroscopic means, mainly ^1^H and ^13^C NMR, including 1D and 2D homo- and heteronuclear experiments and some chemical reactions. The antiproliferative activity of some diterpenoids was determined via the sulforhodamine B method, where guevarain B (**2**) and 6α-hydroxy patagonol acetonide (**7**) showed moderate activity against the K562 line, with IC_50_ (μM) = 33.0 ± 1.3 and 39.8 ± 1.5, respectively. The NO inhibition in the RAW 264.7 macrophage activity via nitrite formation was also determined for some compounds, where 2-oxo-patagonal (**6**), 6α-hydroxy patagonol acetonide (**7**), and 7α-acetoxy-*ent*-clerodan-3,13-dien-18,19:16,15-diolide (**10**) were proven to be active, with IC_50_ (μM) = 26.4 ± 0.4, 17.3 ± 0.5, and 13.7 ± 2.0, respectively.

The hierarchical cluster analysis of the diterpenoid content of the members of section *Holwaya*, including those obtained in this work, indicated a close phytochemical similarity between *S. adenophora* and *S. guevarae*, thus supporting the classification of the latter in section *Holwaya*.

## Figures and Tables

**Table 1:** NMR data (700 MHz) of **1** and **2**.

1 ^a^				2 ^b^		
Position	*δ_C_*	Type	*δ*_H_, Multiplicity (*J* in Hz)	*δ_C_*	Type	*δ*_H_, Multiplicity (*J* in Hz)
1	29.2	CH_2_	1.94, ddd (12.6, 7.0, 1.4)	34.4	CH_2_	2.48, dd (17.8, 13.9)
			1.49, td (12.6, 10.1)			2.32, dd (17.8, 3.6)
2	69.1	CH	4.13, m	199.7	C	
3	127.9	CH	5.19, brs	126.5	CH	5.72, brs
4	146.7	C		172.8	C	
5	45.2	C		45.5	C	
6	75.3	CH	3.48, dt (9.5, 5.6)	73.5	CH	3.70, dd (11.1, 4.8)
7	38.7	CH_2_	1.57, m	37.9	CH_2_	1.69 ddd(13.0, 4.7, 3.7)
						1.62, ddd (13.0, 12.4, 11.1)
8	35.2	CH	1.70, m	34.4	CH	1.73, m
9	38.8	C		38.7	C	
10	45.4	CH	1.35, dd (12.6, 1.4)	44.9	CH	1.88, dd (13.9, 3.6)
11	36.8	CH_2_	1.62, ddd (14.6, 12.7, 5.1)	35.0	CH_2_	1.58, m
			1.55, m			1.47, ddd (14.6, 13.0, 4.9)
12	19.4	CH_2_	2.14, m	18.7	CH_2_	2.16, m
			2.05, m			2.00, m
13	134.3	C		134.3	C	
14	146.2	CH	7.41, p (1.7) *	144.0	CH	7.09, p (1.7) *
15	71.0	CH_2_	4.79, q (1.8)	70.4	CH_2_	4.76, p (1.7)
16	174.7	C		174.3	C	
17	16.0	CH_3_	0.84, d (6.8)	15.5	CH_3_	0.89, d (6.8)
18	22.6	CH_3_	1.84, dd (1.6, 1.7)	23.0	CH_3_	2.14, d (1.3)
19	15.6	CH_3_	1.06, s	13.6	CH_3_	1.12, s
20	18.3	CH_3_	0.75, s	17.4	CH_3_	0.84, s
2 OH			3.6, d (6.1)			
6 OH			3.39, d (5.6)			

^a^ (CD_3_)_2_CO; ^b^ CDCl_3_; * ^4^*J*.

**Table 2:** NMR data of **1a** and **3**.

1a ^a^				3 ^b^		
Position	*δ_C_*	Type	*δ*_H_, Multiplicity (*J* in Hz)	*δ_C_*	Type	*δ*_H_, Multiplicity (*J* in Hz)
1	34.2	CH_2_	2.53, dd (17.0, 14.3)	28.9	CH_2_	2.02, ddt (12.5, 6.8, 1.3)
			2.39, dd (17.0, 3.1)			1.57, td (12.3, 10.3)
2	197.8	C		69.5	CH	4.23, m
3	127.9	CH	5.82, brs	129.6	CH	5.56, brs
4	167.2	C		149.4	C	
5	55.4	C		45.6	C	
6	210.7	C		75.9	CH	3.58, m
7	43.7	CH_2_	2.85, dd (12.7, 12.6)	37.20	CH_2_	1.64, m
			2.19, dd (12.7, 4.0)			
8	40.5	CH	2.13, m	35.9	CH	1.76, m
9	39.3	C		39.2	C	
10	48.2	CH	2.25, dd (14.2, 3.2)	45.6	CH	1.40, brd (12.3)
11	35.4	CH_2_	1.68, ddd (14.2, 12.7, 4.2)	37.2	CH_2_	1.68, ddd (13.7, 12.3, 4.9)
			1.53, ddd (14.6, 12.7, 4.9)			1.61, m
12	19.1	CH_2_	2.14, m	19.7	CH_2_	2.21, m
			2.00, m			2.18, m
13	133.8	C		134.7	C	
14	144.5	CH	7.10, p (1.6) *	147.4	CH	7.36, p (1.5) *
15	70.3	CH_2_	4.76, q (1.8)	72.1	CH_2_	4.82, q (1.7)
16	174.1	C		176.8	C	
17	16.2	CH_3_	1.01, d (6.6)	16.0	CH_3_	0.89, d (6.8)
18	21.4	CH_3_	2.17, d (1.3)	65.5	CH_2_	4.08, d (13.5)
						4.23, brd (13.5)
19	18.4	CH_3_	1.51, s	16.5	CH_3_	1.13, s
20	17.9	CH_3_	1.06, s	18.6	CH_3_	0.79, s

^a^ 500 MHz, ^b^ CDCl_3_, 700 MHz; CD_3_OD; * ^4^*J*.

**Table 3:** NMR data (500 MHz, CDCl_3_) of **4** and **5**.

4				5		
Position	*δ_C_*	Type	*δ*_H_, Multiplicity (*J* in Hz)	*δ_C_*	Type	*δ*_H_, Multiplicity (*J* in Hz)
1	35.3	CH_2_	2.44, dd (17.8, 13.5)	34.7	CH_2_	2.54, dd (17.8, 13.9)
			2.37, dd (17.8, 4.3)			2.37, dd (17.8, 3.4)
2	200.1	C		200.2	C	
3	121.4	CH	6.10, br s	125.4	CH	5.97, br s
4	173.6	C		170.6	C	
5	39.2,	C		35.0	C	
6	34.6	CH_2_	1.57, m	73.8	CH	3.83, dd (8.7, 6.9)
			1.45, m			
7	26.7	CH_2_	1.53, m	37.0	CH_2_	1.63, m
8	36.2	CH	1.58, m			1.55, m
9	38.9	C		34.5	CH	1.70, m
10	45.8	CH	1.98, dd (13.5, 4.3)	45.1	CH	1.91, dd (13.9, 3.6)
11	35.1	CH_2_	1.54, m	35.0	CH_2_	2.51, ddd (13.7, 12.3, 4.9)
						1.49, m
12	18.9	CH_2_	2.21, m	18.8	CH_2_	2.13, m
			2.04, m			1.96, m
13	134.4	C		134.2	C	
14	143.9	CH	7.09, q (1.7)	144.2	CH	7.10, p (1.4)
15	70.4	CH_2_	4.77, m	70.4	CH_2_	477, q (1.7)
16	173.6	C		170.6	C	
17	15.8	CH_3_	0.87, d (6.4)	14.8	CH_3_	0.91, d (6.4)
18	60.6	CH_2_	4.40, dd (17.6, 1.8)	65.1	CH_2_	4.51, d (15.0)
			4.35, dd (17.6, 1.8)			4.37, br d (15.0)
19	19.6	CH_3_	1.20, s	17.6	CH_3_	1.22, s
20	17.9	CH_3_	0.85, s	15.5	CH_3_	0.85, s

**Table 4:** NMR data (500 MHz, CDCl_3_) of **6**.

6				6			
Position	*δ_C_*	Type	*δ*_H_ (*J* in Hz)	Position	*δ_C_*	Type	*δ*_H_ (*J* in Hz)
1	35.3	CH_2_	2.65 dd (18.1, 14.1)	11	35	CH_2_	1.62, m
			2.56, dd (18.1, 3.6)				1.51, ddd (14.7, 12.8, 4.8)
2	199.9	C		12	18.7	CH_2_	2.18, tdd (12.9, 4.3, 2.0)
3	141.1	CH	6.43, br s				1.97, m
4	163.8	C		13	134.1	C	
5	45.8	C		14	144.1	CH	7.10, br s
6	72.1	CH	3.75, dd (11.1, 4.7)	15	70.4	CH_2_	4.77, q (2.1)
7	35.5	CH_2_	177, ddd (13.3, 4.8, 3.0)	16	174.2	C	
			1.65, m	17	15.6	CH_3_	0.84, d (6.6)
8	34.2	CH	1.71, m	18	197.5	CH	9.75, s
9	38.6	C		19	15.2	CH_3_	1.13, s
10	44.8	CH	1.95, dd (14.1, 3.5)	20	17.1	CH_3_	0.78, s
				6-OH			4.63, s

**Table 5:** IC_50_ (µM) values of the antiproliferative activity for compounds **2** and **7** against K562.

Compounds	IC_50_
2	33.1 ± 1.3
7	39.8 ± 1.5
Adriamycin	0.2 ± 0.0

**Table 6:** IC_50_ (µM) values for the inhibition of nitrite production for compounds **7**, **8**, and **11**.

Compound	IC_50_ (µM)
**6**	26.4 ± 0.4
**7**	17.3 ± 0.5
**10**	13.7 ± 2.0
Aminoguanidine	26.2 ± 0.4

The results are expressed as the mean ± S.E.M. of three determinations.

## Data Availability

The crystallographic data for compounds **1** and **10** have been deposited at the Cambridge Crystallographic Data Centre under the reference numbers CCDC 2430636 and CCDC 2430637, respectively. Copies of the data can be obtained free of charge via application to the CCDC, 2 Union Road, Cambridge CB2 IEZ, UK. Fax: +44-(0)1223-336033 or email: deposit@ccdc.cam.ac.uk.

## Supplementary Materials

The following supporting information can be downloaded at https://www.mdpi.com/article/10.3390/molecules30071628/s1, Figures S1–S60: Copies of the 1D and 2D NMR spectra of compounds **1**–**9**; Figures S61–S63: Comparison between the experimental and calculated ECD for the enantiomers of **11**, **12**, and **13**; Tables S1 and S2: Crystallographic data for compounds **1** and **10**; Table S3: Screening for the antiproliferative activity of the isolated compounds from *Salvia guevarae* (25 μM) and the dichloromethane extract of *S. guevarae* (25 ppm); Table S4: Screening for the inhibition of nitrite (NO) production for compounds **1**–**3** and **5**–**10** in the RAW 264.7 macrophages (25 μM); Spectroscopic data of compounds **7**–**13**.
