# Supplementary material for: The Antiproliferative Activity and NO Inhibition of Neo-Clerodane Diterpenoids from Salvia guevarae in RAW 264.7 Macrophages
Source: Molecules. 2025 Apr 5;30(7):1628. doi: 10.3390/molecules30071628 (PMC11990168; doi:10.3390/molecules30071628)
Supplement: Supplementary file 1 [file molecules-30-01628-s001.zip › molecules-3552094-supplementary.pdf]

The Antiproliferative Activity and NO Inhibition of *Neo*-Clerodane Diterpenoids from *Salvia guevarae* in RAW 264.7 Macrophages

Juan Pablo Torres-Medicis [1], Celia Bustos-Brito [1], Leovigildo Quijano [1], Brenda Y. Bedolla-García [2], Sergio Zamudio [3], Teresa Ramírez-Apan [1], Diego Martínez-Otero [1] and Baldomero Esquivel [1,*]

[1] Instituto de Química, Universidad Nacional Autónoma de México, Circuito Exterior, Ciudad Universitaria, Ciudad de México 04510, Mexico; quijano@unam.mx (L.Q.); diegomtz@unam.mx (D.M.-O.)
[2] Instituto de Ecología, A.C., Centro Regional del Bajío, Apartado Postal 386, Pátzcuaro 61600, Mexico; brenda.bedolla@inecol.mx
[3] Independent Researcher, Apartado Postal 392, Pátzcuaro 61600, Mexico
* Correspondence: baldo@unam.mx

Supplementary Materials

Figure S1. ^{1}H-NMR spectrum of **1** ($(CD_3)_2CO$, 700 MHz)........5
Figure S2. APT spectrum of **1** ($(CD_3)_2CO$, 175 MHz).........5
Figure S3. HSQC spectrum of **1** ($(CD_3)_2CO$, 700 MHz).........6
Figure S4. HMBC spectrum of **1** ($(CD_3)_2CO$, 700 MHz). 6
Figure S5. ^{1}H-^{1}H COSY spectrum of **1** ($(CD_3)_2CO$, 700 MHz).........7
Figure S6. NOESY spectrum of **1** ($(CD_3)_2CO$, 700 MHz).........7
Figure S7. ^{1}H-NMR spectrum of **1a** ($CDCl_3$, 700 MHz). 8
Figure S8. APT spectrum of **1a** ($CDCl_3$, 175 MHz). 8
Figure S9. HSQC spectrum of **1a** ($CDCl_3$, 700 MHz). 9
Figure S10. HMBC spectrum of **1a** ($CDCl_3$, 700 MHz).........9
Figure S11. ^{1}H-^{1}H COSY spectrum of **1a** ($CDCl_3$, 700 MHz).........10
Figure S12. NOESY spectrum of **1a** ($CDCl_3$, 700 MHz).........10
Figure S13. ^{1}H-NMR spectrum of **2** ($CDCl_3$, 700 MHz). 11
Figure S14. APT spectrum of **2** ($CDCl_3$, 175 MHz). 11
Figure S15. HSQC spectrum of **2** ($CDCl_3$, 700 MHz). 12
Figure S16. HMBC spectrum of compound **2** ($CDCl_3$, 700 MHz). 12
Figure S17. ^{1}H-^{1}H COSY spectrum of **2** ($CDCl_3$, 700 MHz).........13
Figure S18. NOESY spectrum of **2** ($CDCl_3$, 700 MHz).........13
Figure S19. ^{1}H-NMR spectrum of **3** (CD_3OD, 700 MHz). 14
Figure S20. APT spectrum of **3** (CD_3OD, 700 MHz). 14
Figure S21. HSQC spectrum of **3** (CD_3OD, 700 MHz). 15
Figure S22. HMBC spectrum of **3** (CD_3OD, 700 MHz).........15
Figure S23. ^{1}H-^{1}H COSY spectrum of **3** (CD_3OD, 700 MHz).........16
Figure S24. NOESY spectrum of **3** (CD_3OD, 700 MHz).........16
Figure S25. ^{1}H-NMR spectrum of **4** ($CDCl_3$, 700 MHz). 17
Figure S26. APT spectrum of **4** ($CDCl_3$, 175 MHz) 17
Figure S27. HSQC spectrum of **4** ($CDCl_3$, 700 MHz). 18
Figure S28. HMBC spectrum of **4** ($CDCl_3$, 700 MHz).........18
Figure S29. ^{1}H-^{1}H COSY spectrum of **4** ($CDCl_3$, 700 MHz).........19
Figure S30. NOESY spectrum of **4** ($CDCl_3$, 700 MHz).........19
Figure S31. ^{1}H-NMR spectrum of **5** ($CDCl_3$, 700 MHz). 20
Figure S32. APT spectrum of **5** ($CDCl_3$, 700 MHz). 20
Figure S33. HSQC spectrum of **5** ($CDCl_3$, 700 MHz). 21

Figure S34. HMBC spectrum of **5** ($CDCl_3$, 700 MHz). 21
Figure S35. ^{1}H-^{1}H COSY spectrum of **5** ($CDCl_3$, 700 MHz). 22
Figure S36. NOESY spectrum of **5** ($CDCl_3$, 700 MHz). 22
Figure S37. ^{1}H-NMR spectrum of **6** ($CDCl_3$, 700 MHz). 23
Figure S38. APT spectrum of **6** ($CDCl_3$, 175 MHz) 23
Figure S39. HSQC spectrum of **6** ($CDCl_3$, 700 MHz). 24
Figure S40. HMBC spectrum of **6** ($CDCl_3$, 700 MHz) 24
Figure S41. ^{1}H-^{1}H COSY spectrum of **6** ($CDCl_3$, 700 MHz). 25
Figure S42. NOESY spectrum of **6** ($CDCl_3$, 700 MHz). 25
Figure S43. ^{1}H-NMR spectrum of **7** ($CDCl_3$, 400 MHz). 26
Figure S44. APT spectrum of **7** ($CDCl_3$, 400 MHz). 26
Figure S45. HSQC spectrum of **7** ($CDCl_3$, 400 MHz). 27
Figure S46. HMBC spectrum of **7** ($CDCl_3$,400 MHz). 27
Figure S47. ^{1}H-^{1}H COSY spectrum of **7** ($CDCl_3$, 400 MHz). 28
Figure S48. NOESY spectrum of **7** ($CDCl_3$, 400 MHz). 28
Figure S49. ^{1}H-NMR spectrum of **8** ($CDCl_3$, 700 MHz). 29
Figure S50. APT spectrum of **8** ($CDCl_3$, 700 MHz). 29
Figure S51. HSQC spectrum of **8** ($CDCl_3$, 700 MHz). 30
Figure S52. HMBC spectrum of **8** ($CDCl_3$, 700 MHz). 30
Figure S53. ^{1}H-^{1}H COSY spectrum of **8** ($CDCl_3$, 700 MHz). 31
Figure S54. NOESY spectrum of **8** ($CDCl_3$, 700 MHz). 31
Figure S55. ^{1}H-NMR spectrum of **9** ($CDCl_3$, 700 MHz) 32
Figure S56. APT spectrum of **9** ($CDCl_3$,175 MHz). 32
Figure S57. HSQC spectrum of **9** ($CDCl_3$, 700 MHz). 33
Figure S58. HMBC spectrum of **9** ($CDCl_3$, 700 MHz). 33
Figure S59. ^{1}H-^{1}H COSY spectrum of 9 ($CDCl_3$,700 MHz). 34
Figure S60. NOESY spectrum of **9** ($CDCl_3$,700 MHz). 34
Figure S61. Comparison between the experimental (black line) and the calculated ECD spectra for the enantiomers (5*S*, 8*S*, 9*R*, 10*R*)**-11** (blue line) and (5*R*, 8*R*, 9*S*, 10*S*)**-11** (red line). 35
Figure S62. Comparison between the experimental (black line) and the calculated ECD spectra for the enantiomers (5S, 7R, 8*S*, 9*R*, 10*R*)**-12** (blue line) and (5*R*, 7*S*, 8*R*, 9*S*, 10*S*)**-12** (red line). 35
Figure S63. Comparison between the experimental (black line) and the calculated ECD spectra for the enantiomers (5*S*, 8*R*, 9*S*, 10*R*)**-13** (blue line) and (5R, 8*S*, 9*R*, 10*S*)**-13** (red line). 35
Table S1. Crystallographic data for compounds **1** and **10**. 36
Table S2. Hydrogen bonds for **1** [Å and °]. 36
Table S3. Screening for antiproliferative activity of the isolated compounds from *Salvia*

guevarae (25 μM) and dichloromethane extract of *S. guevarae* (25 ppm). 37

Table S4. Screening for inhibition of nitrite (NO) production for compounds (**1-3**, **5-10**) in macrophages RAW 264.7 (25 μM). 37

Spectroscopic data of compounds **7-13**... 38

References 40

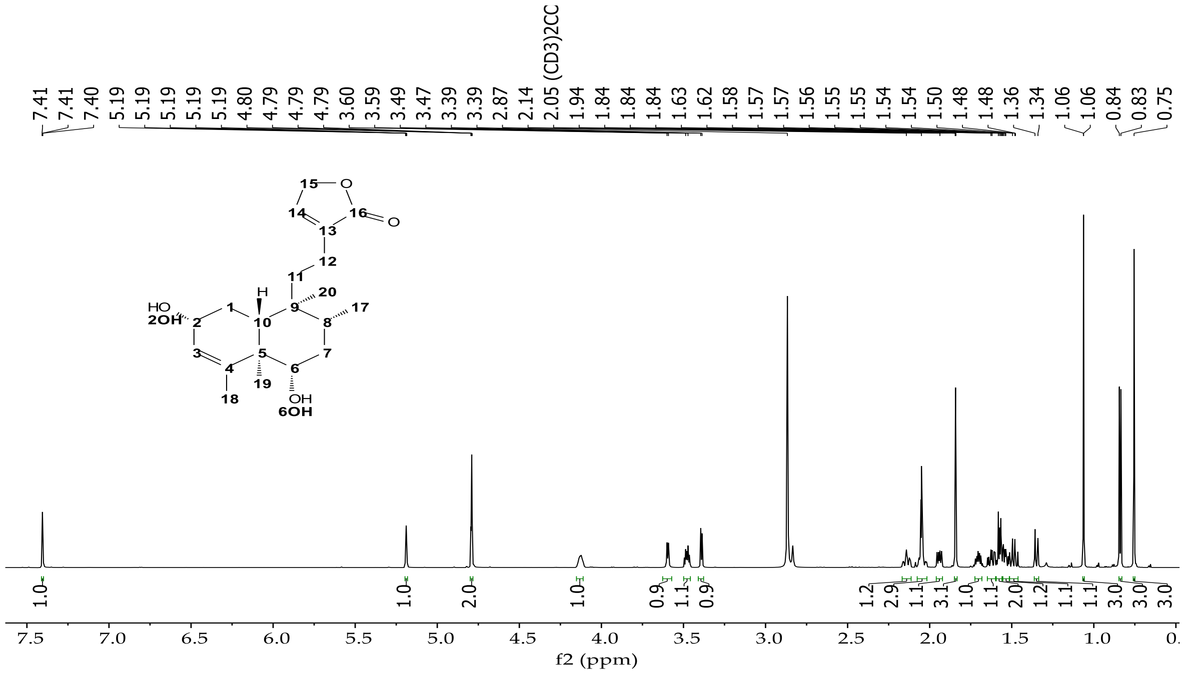

Figure S1. ^{1}H-NMR spectrum of **1** (($(CD_3)_2CO$, 700 MHz).

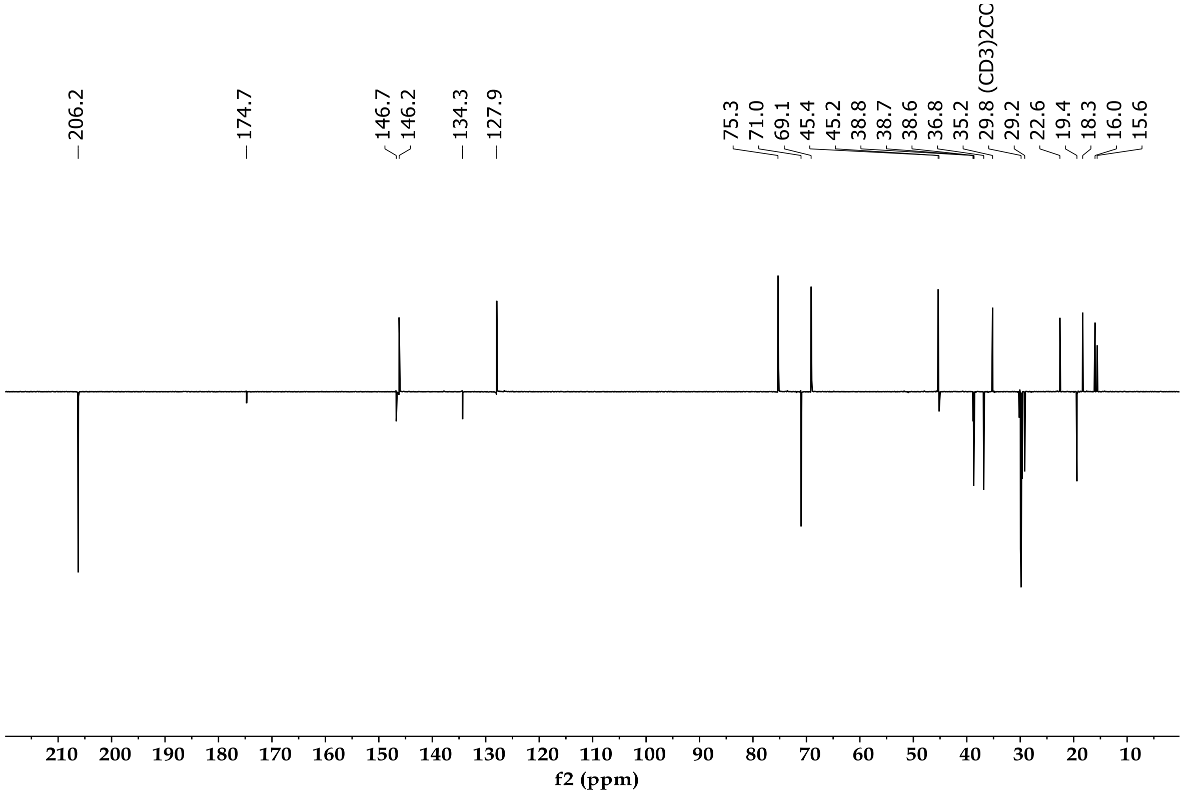

Figure S2. APT spectrum of **1** ($(CD_3)_2CO$, 175 MHz).

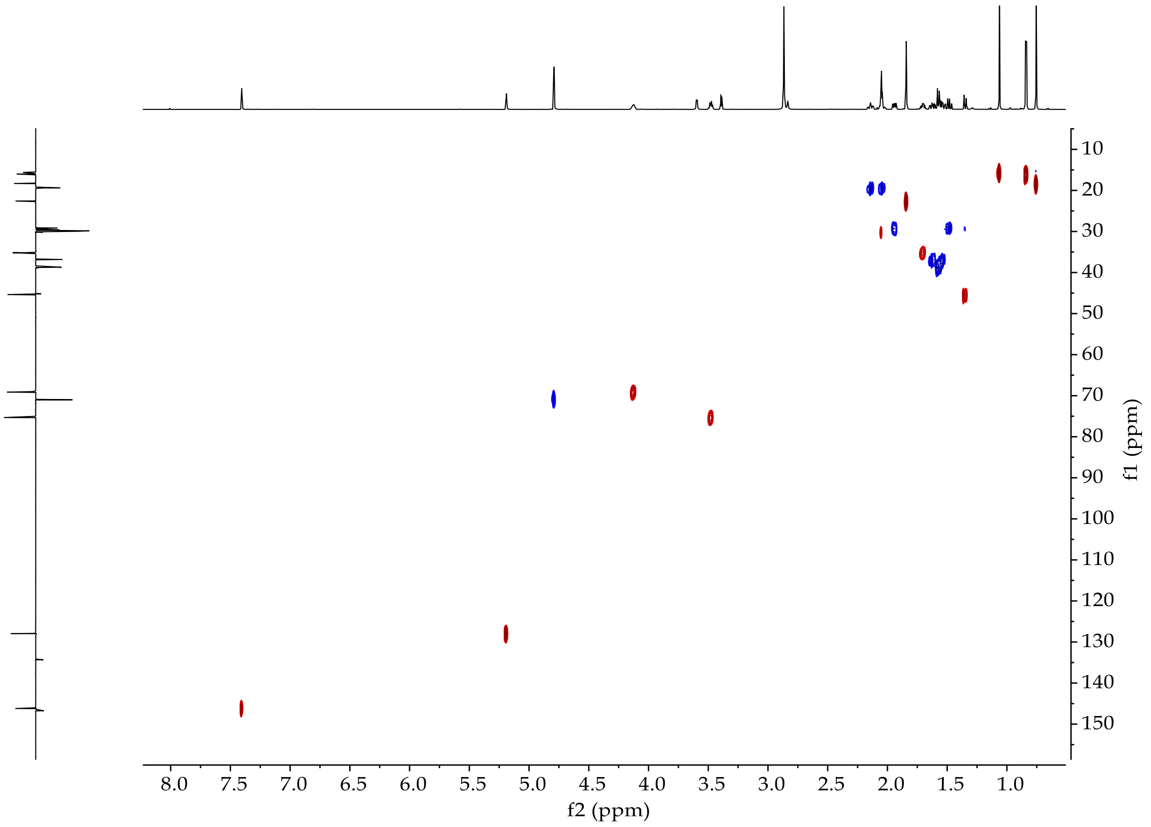

Figure S3. HSQC spectrum of **1** ($(CD_3)_2CO$, 700 MHz).

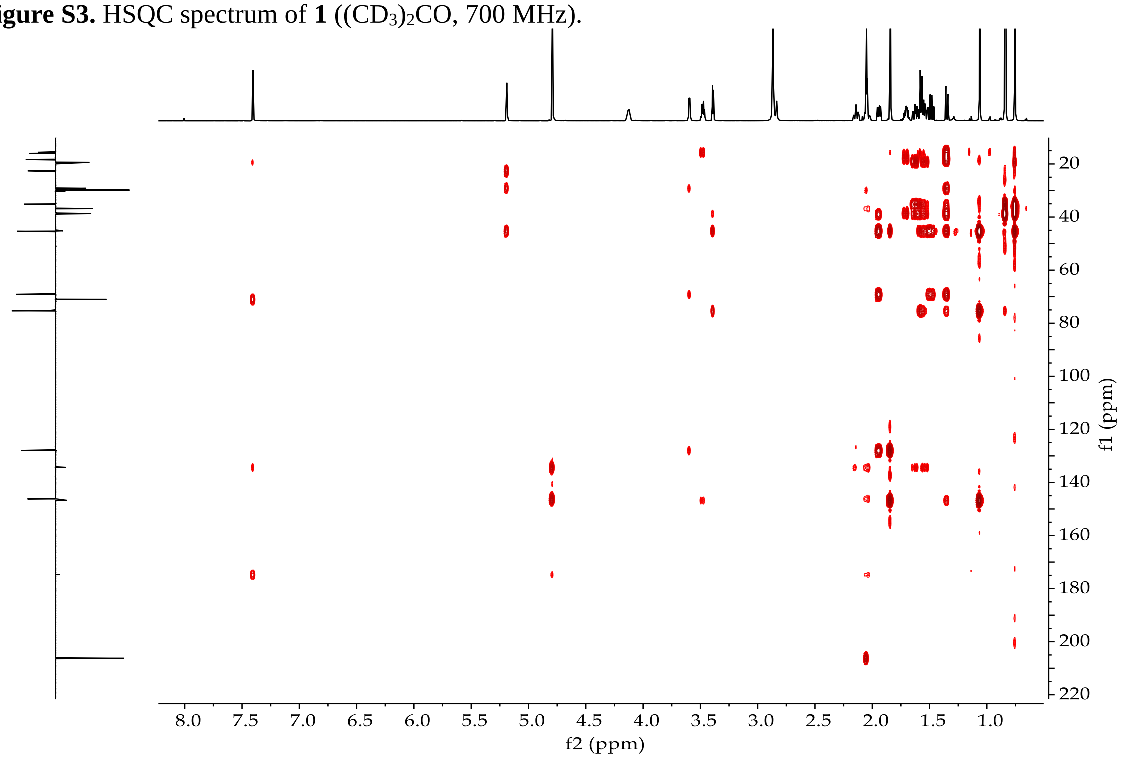

Figure S4. HMBC spectrum of **1** ($(CD_3)_2CO$, 700 MHz).

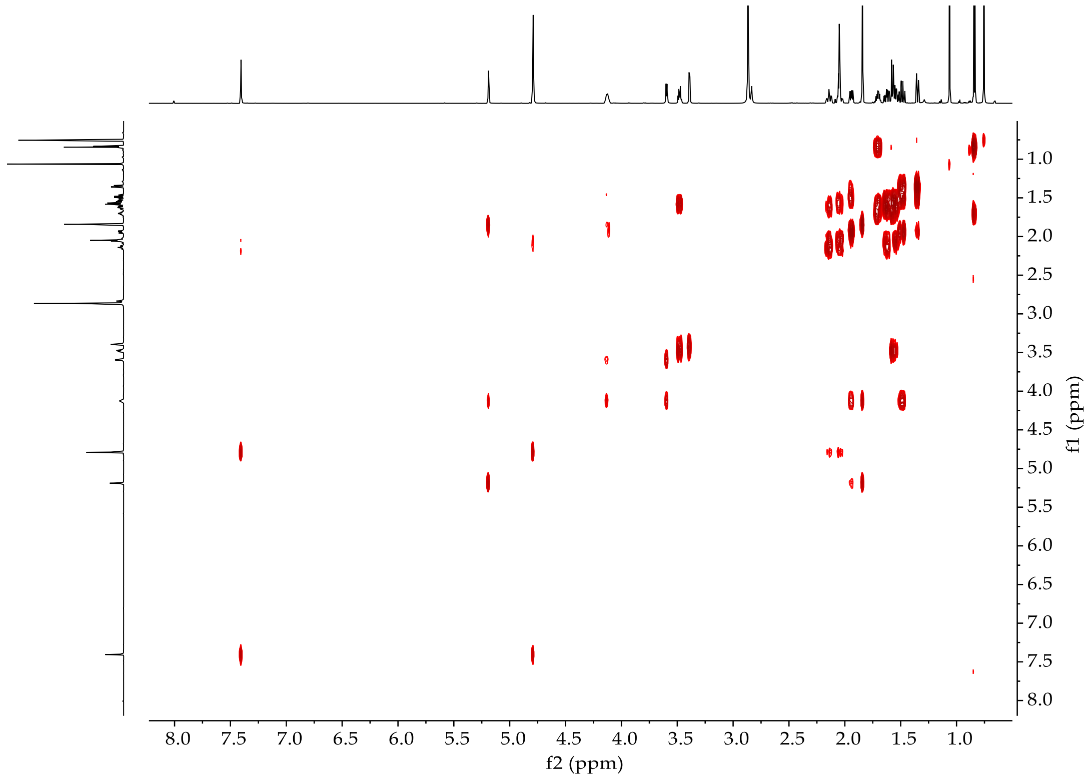

Figure S5. ^{1}H-^{1}H COSY spectrum of **1** ($(CD_3)_2CO$, 700 MHz).

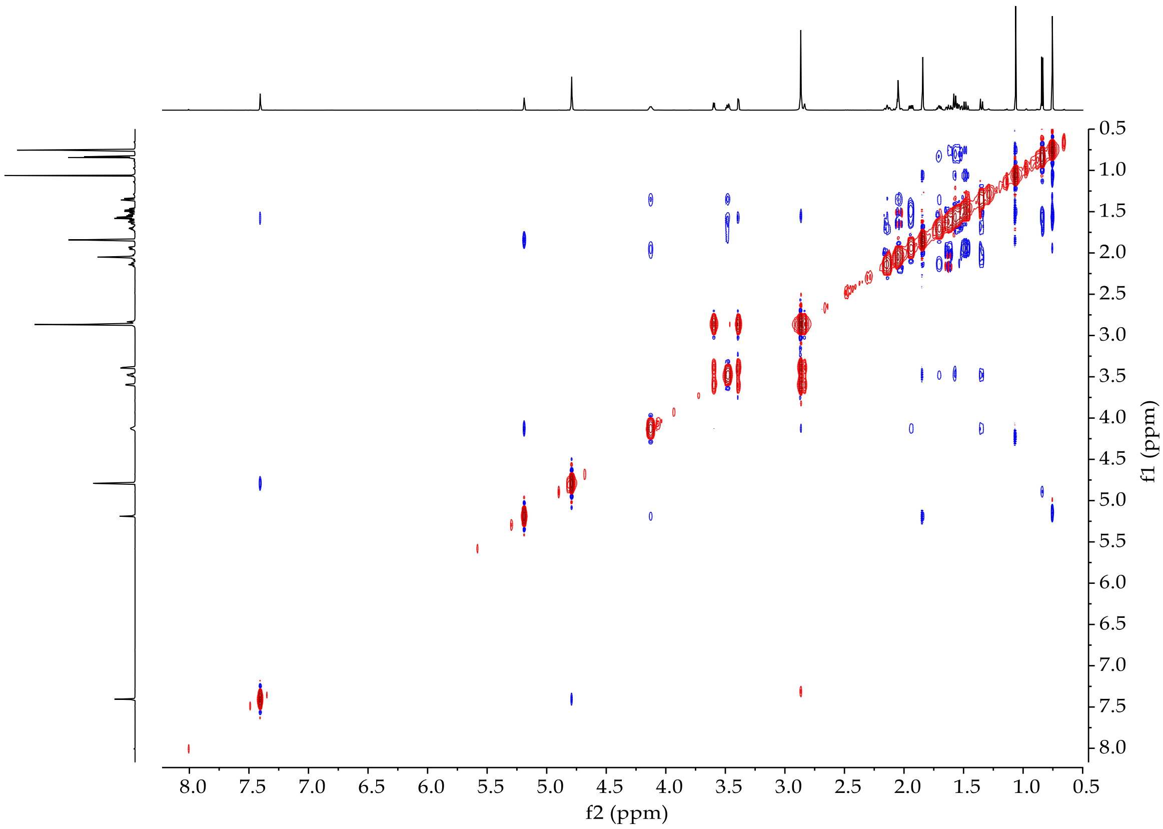

Figure S6. NOESY spectrum of **1** ($(CD_3)_2CO$, 700 MHz).

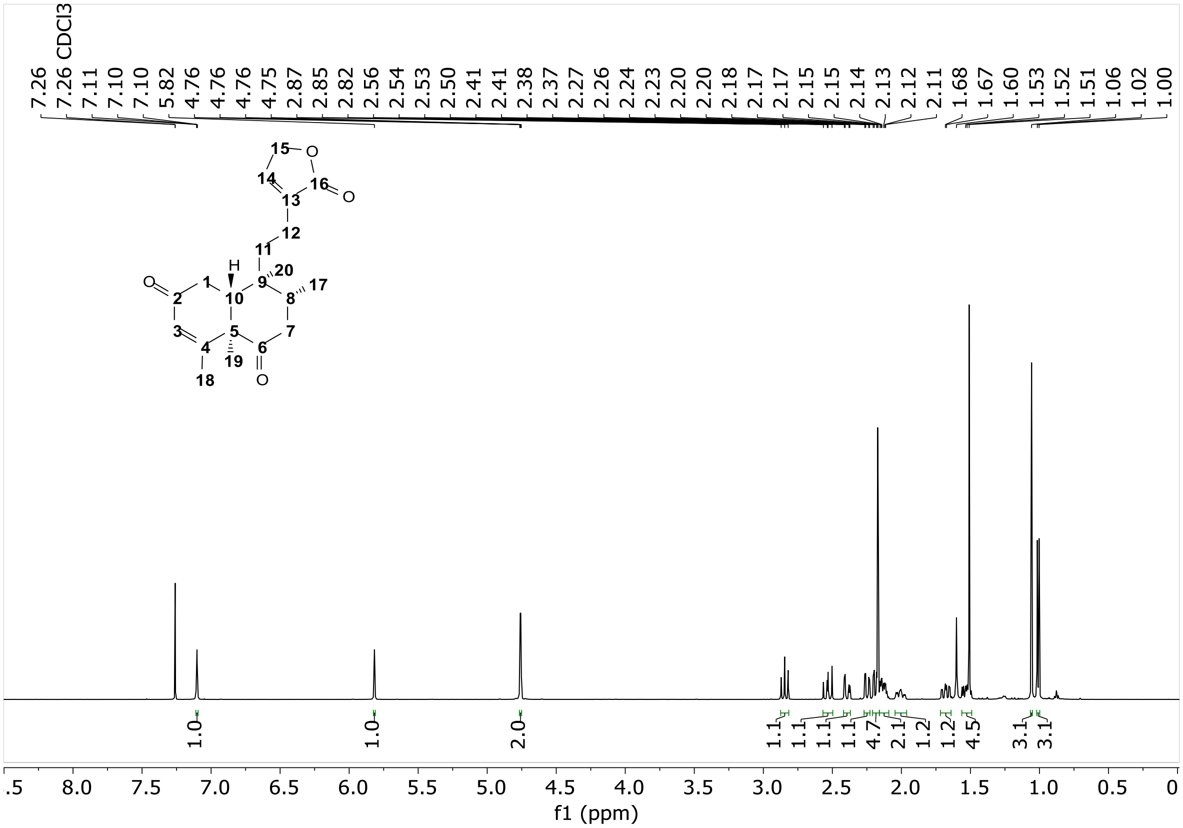

Figure S7. ^{1}H-NMR spectrum of **1a (**$CDCl_3$, 700 MHz)**.**

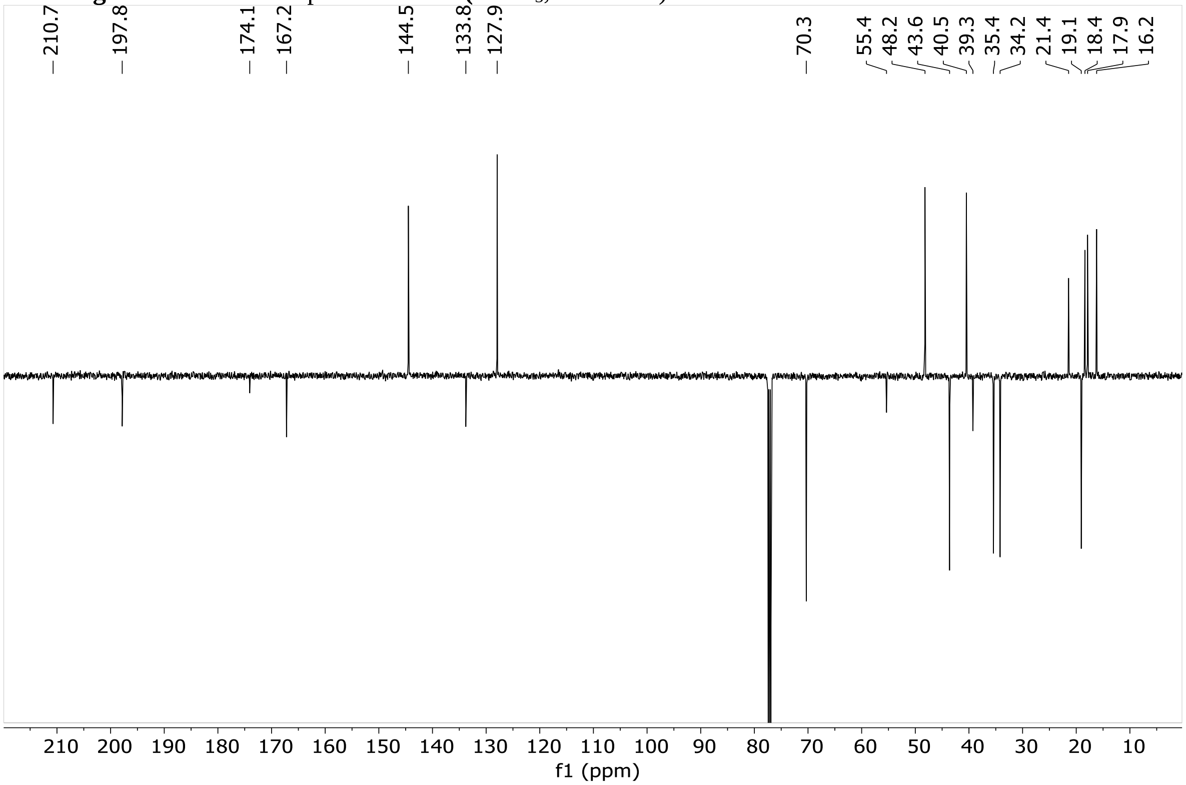

Figure S8. APT spectrum of **1a** ($CDCl_3$, 175 MHz).

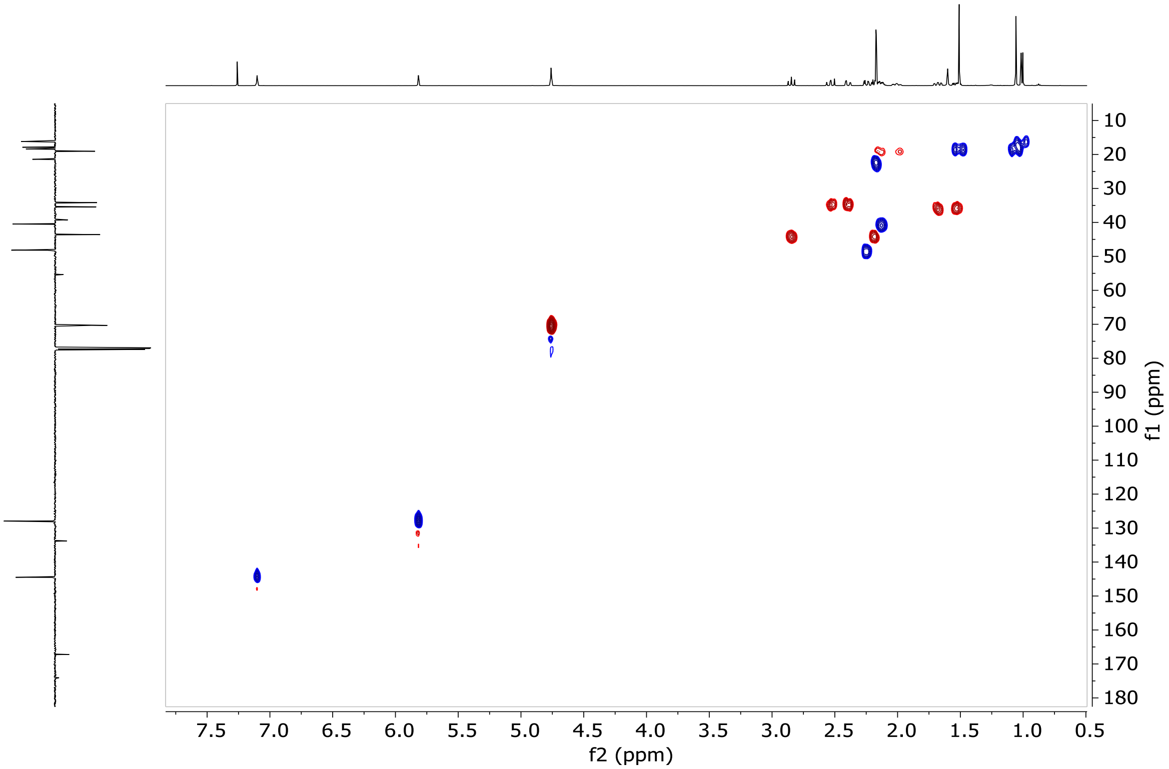

Figure S9. HSQC spectrum of **1a** ($CDCl_3$, 700 MHz).

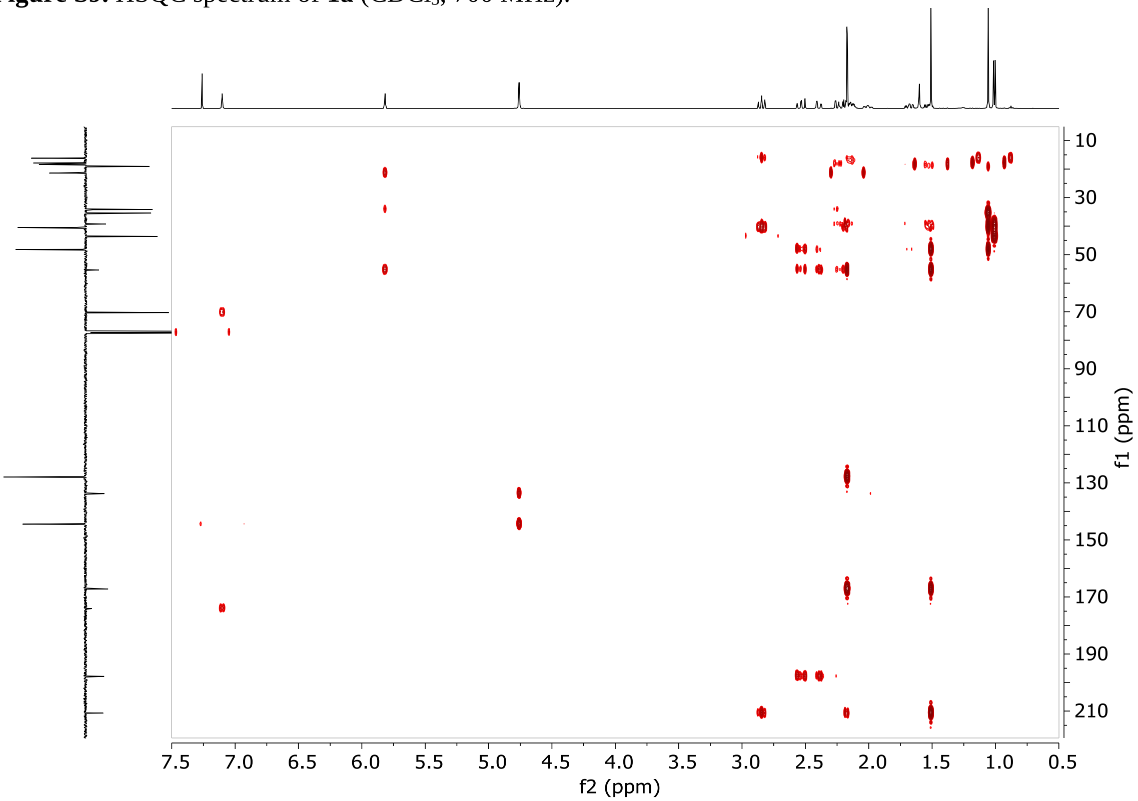

Figure S10. HMBC spectrum of **1a** ($CDCl_3$, 700 MHz).

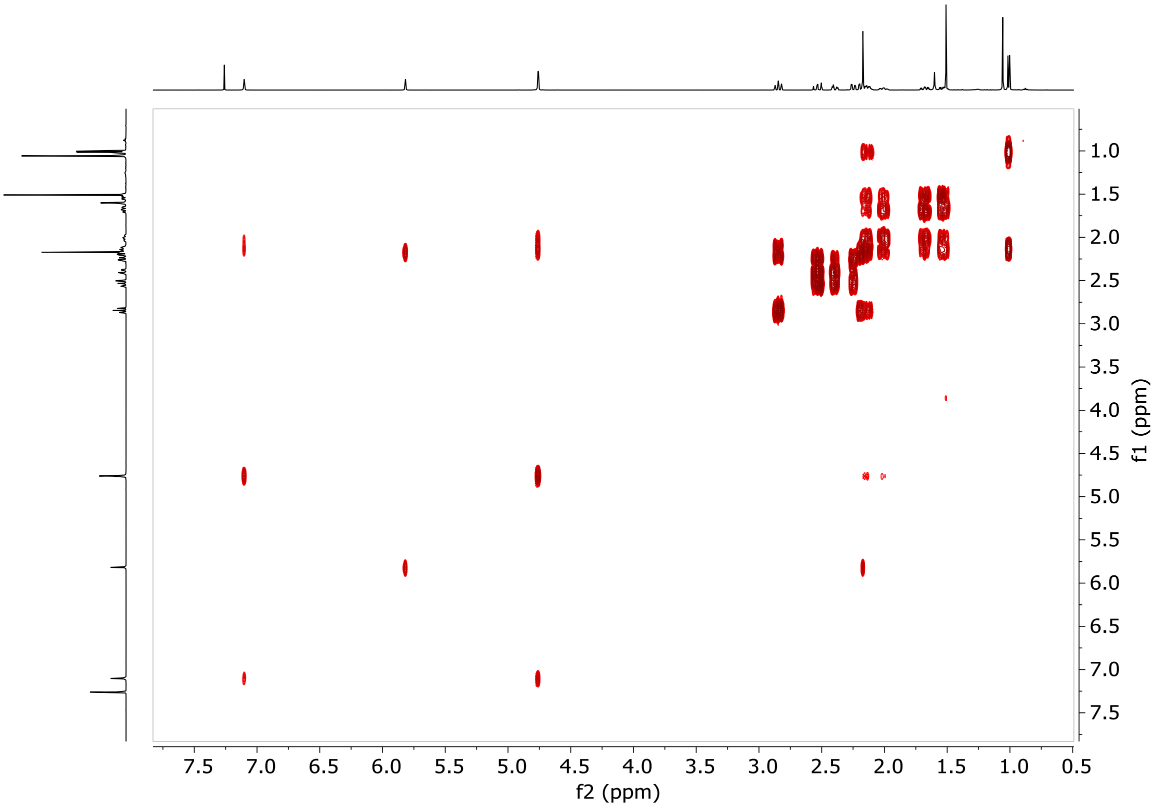

Figure S11. ^{1}H-^{1}H COSY spectrum of **1a** ($CDCl_3$, 700 MHz).

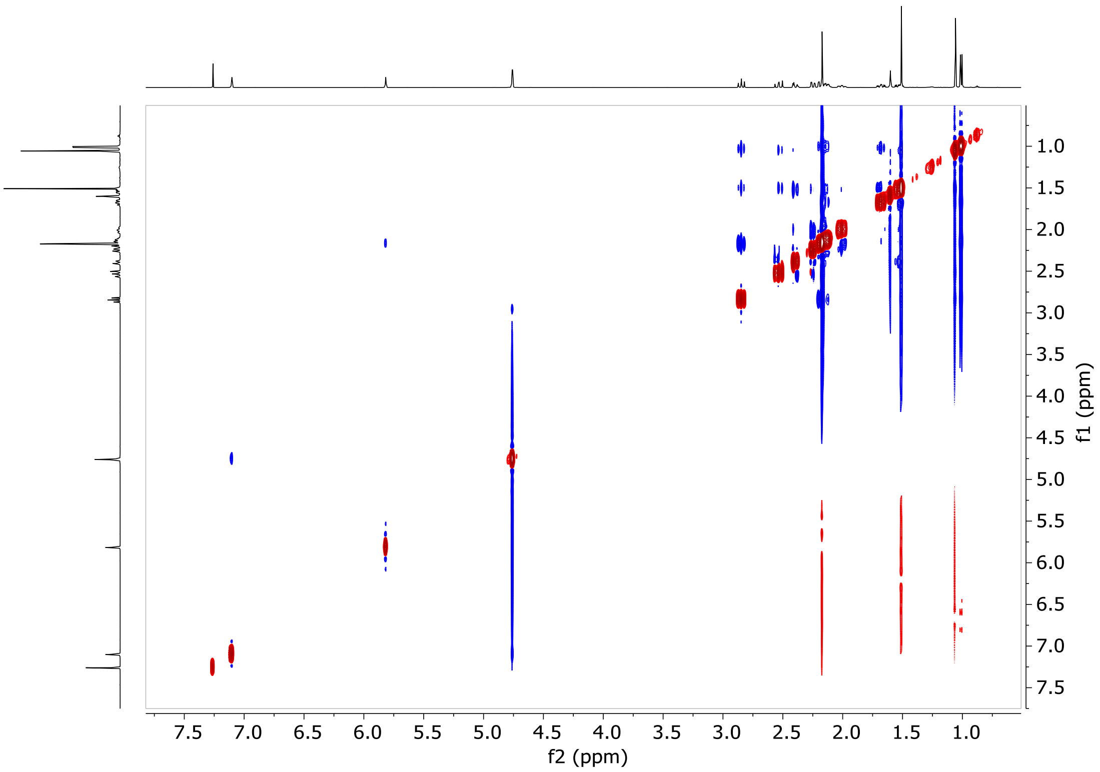

Figure S12. NOESY spectrum of **1a** ($CDCl_3$, 700 MHz).

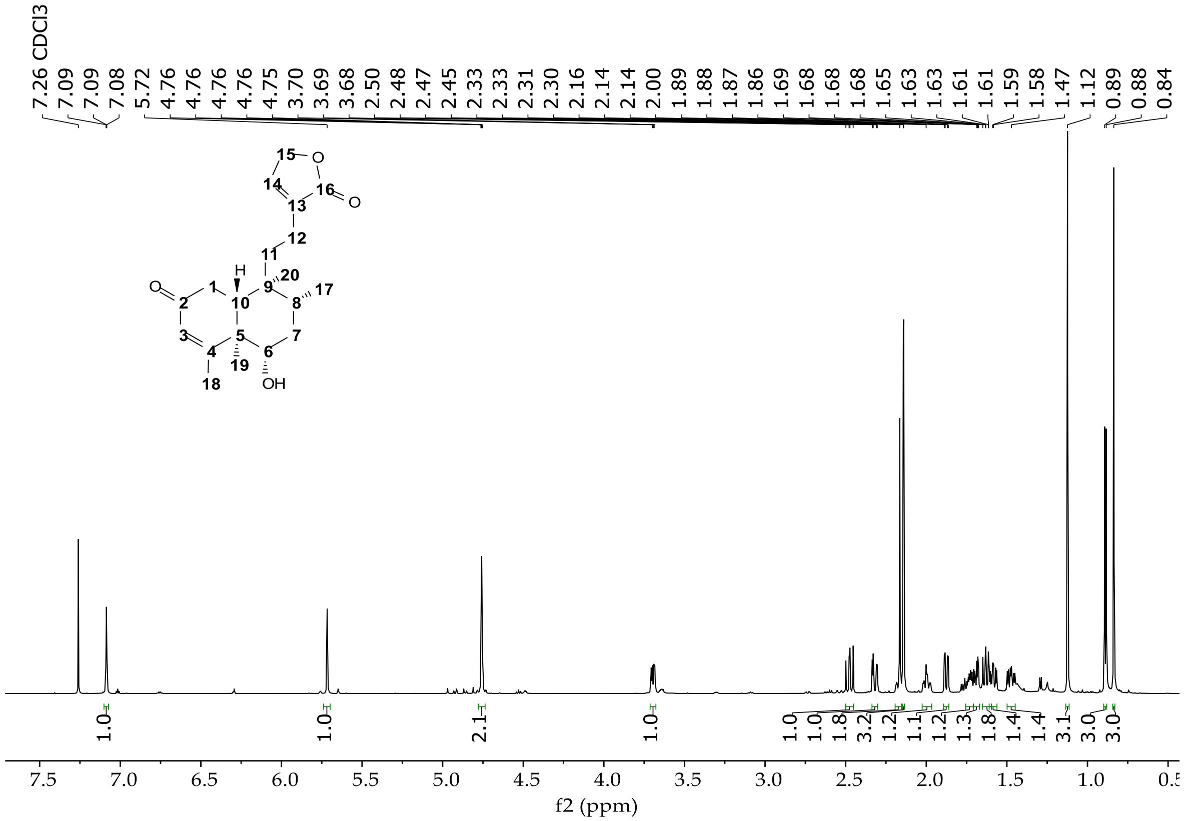

Figure S13. ^{1}H-NMR spectrum of **2** ($CDCl_3$, 700 MHz).

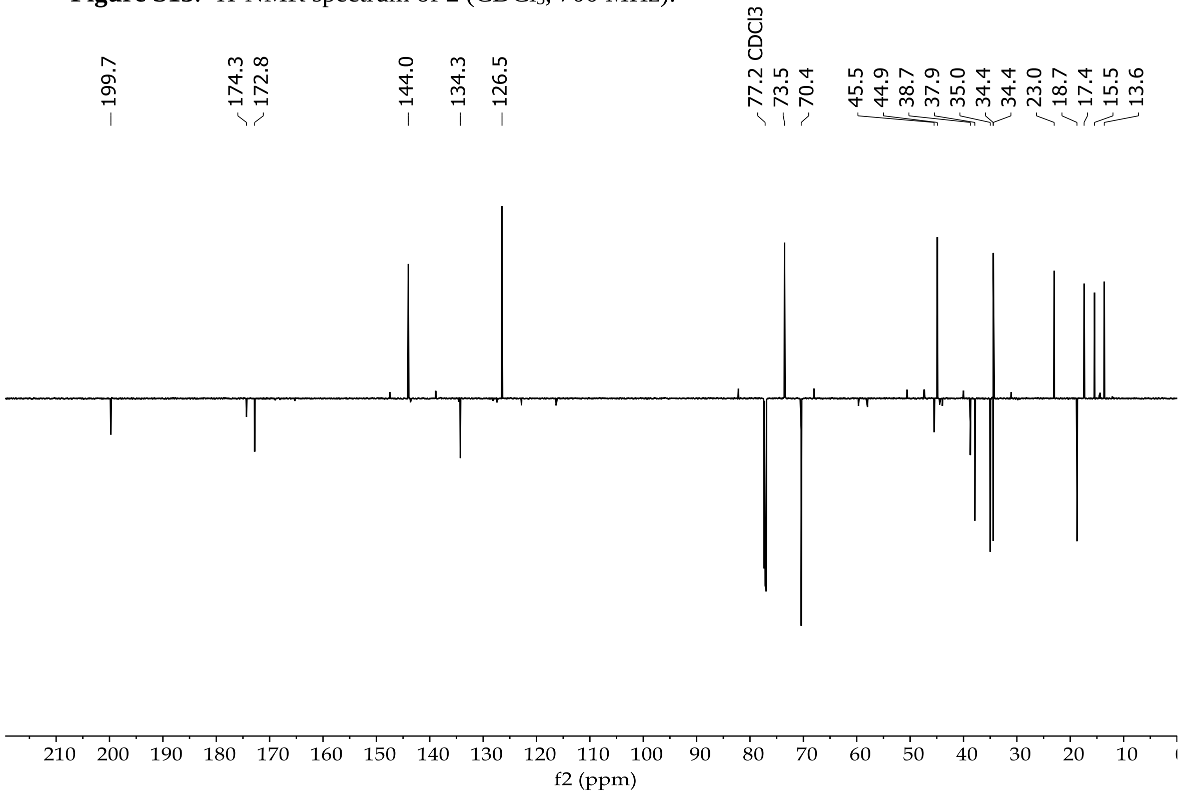

Figure S14. APT spectrum of **2** ($CDCl_3$, 175 MHz).

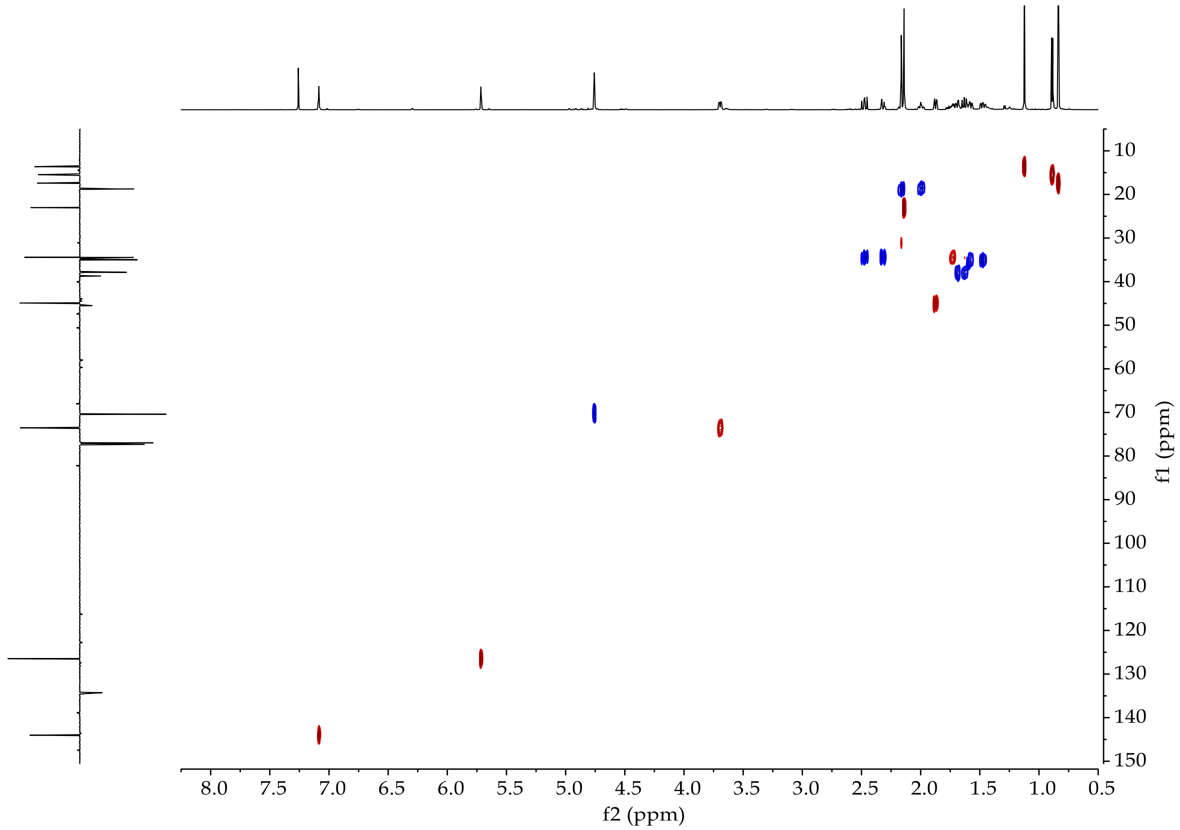

Figure S15. HSQC spectrum of **2** ($CDCl_3$, 700 MHz).

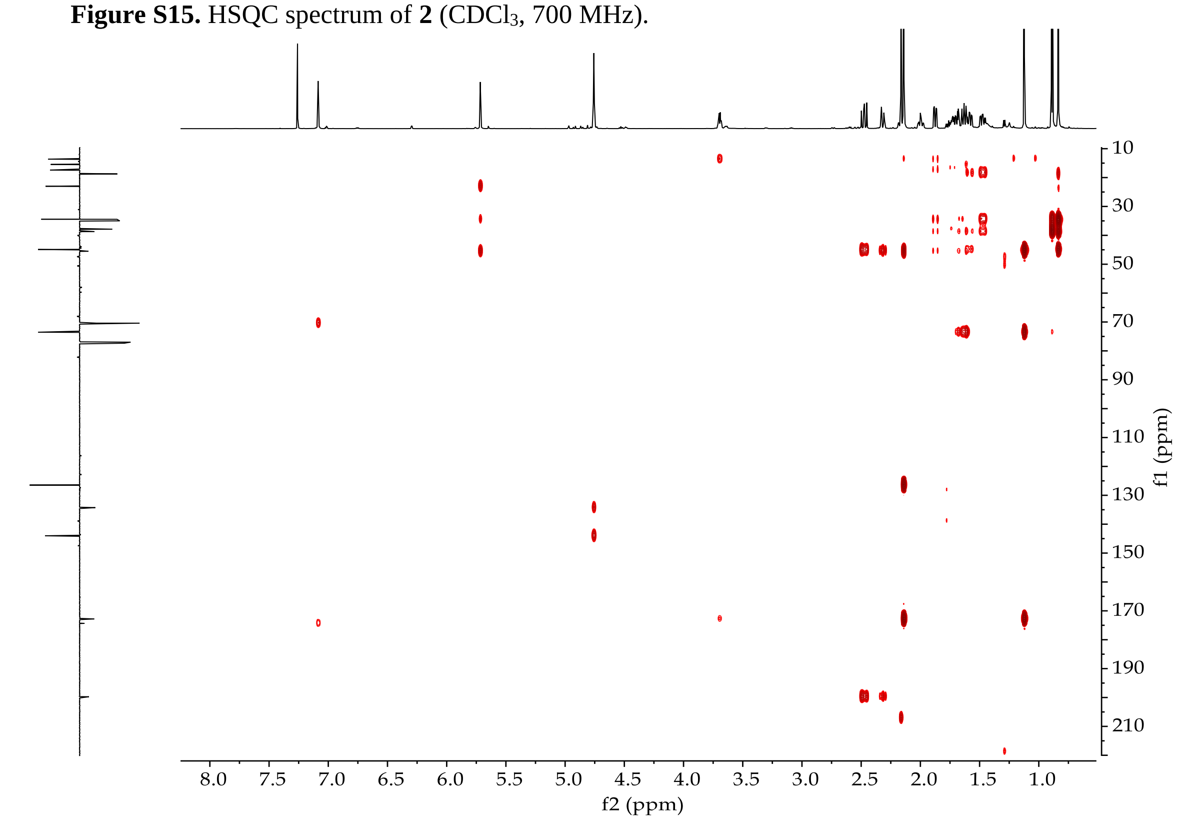

Figure S16. HMBC spectrum of compound **2** ($CDCl_3$, 700 MHz).

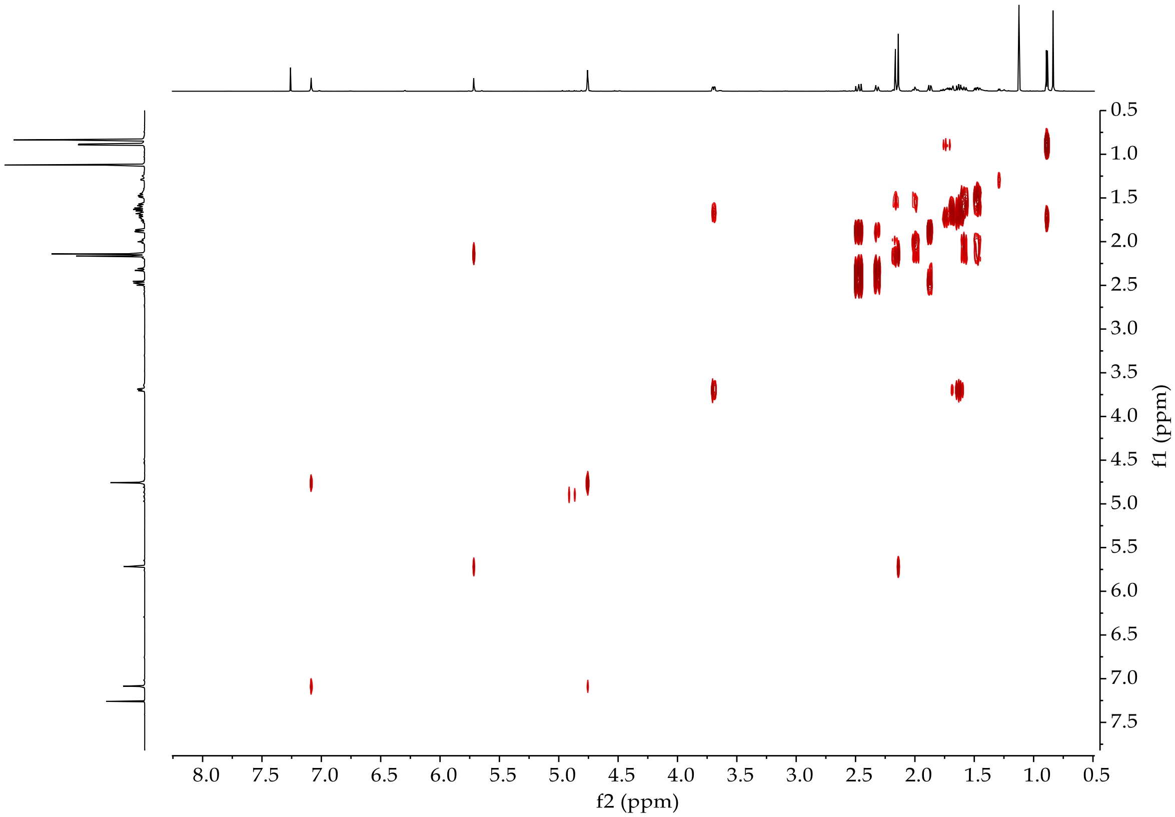

Figure S17. ^{1}H-^{1}H COSY spectrum of **2** ($CDCl_3$, 700 MHz).

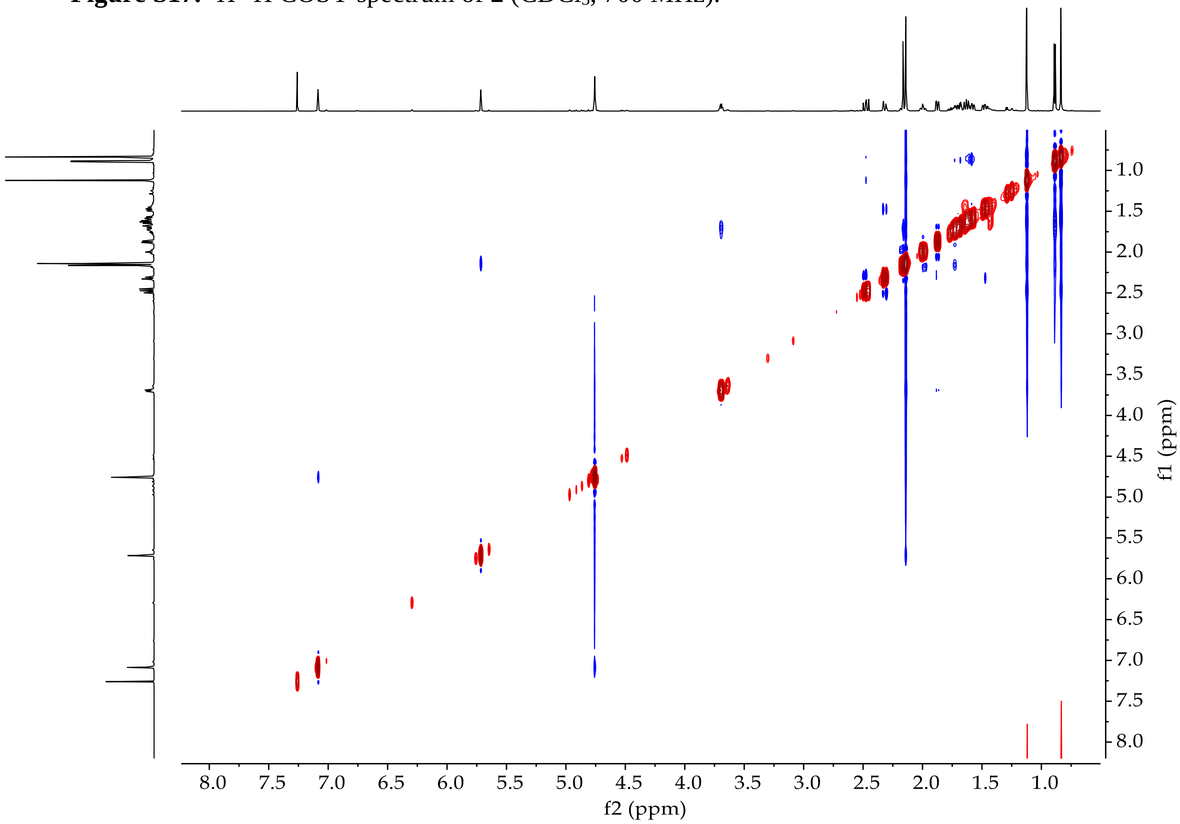

Figure S18. NOESY spectrum of **2** ($CDCl_3$, 700 MHz).

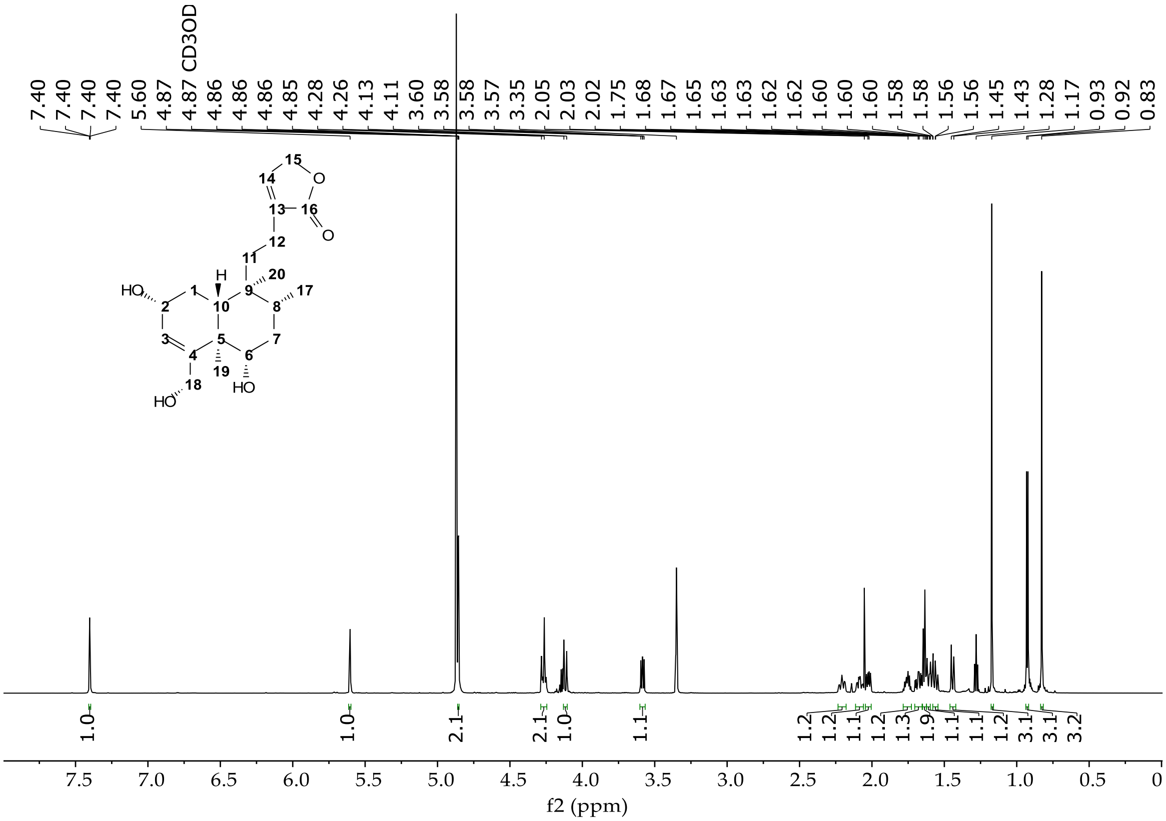

Figure S19. ^{1}H-NMR spectrum of **3** (CD_3OD, 700 MHz).

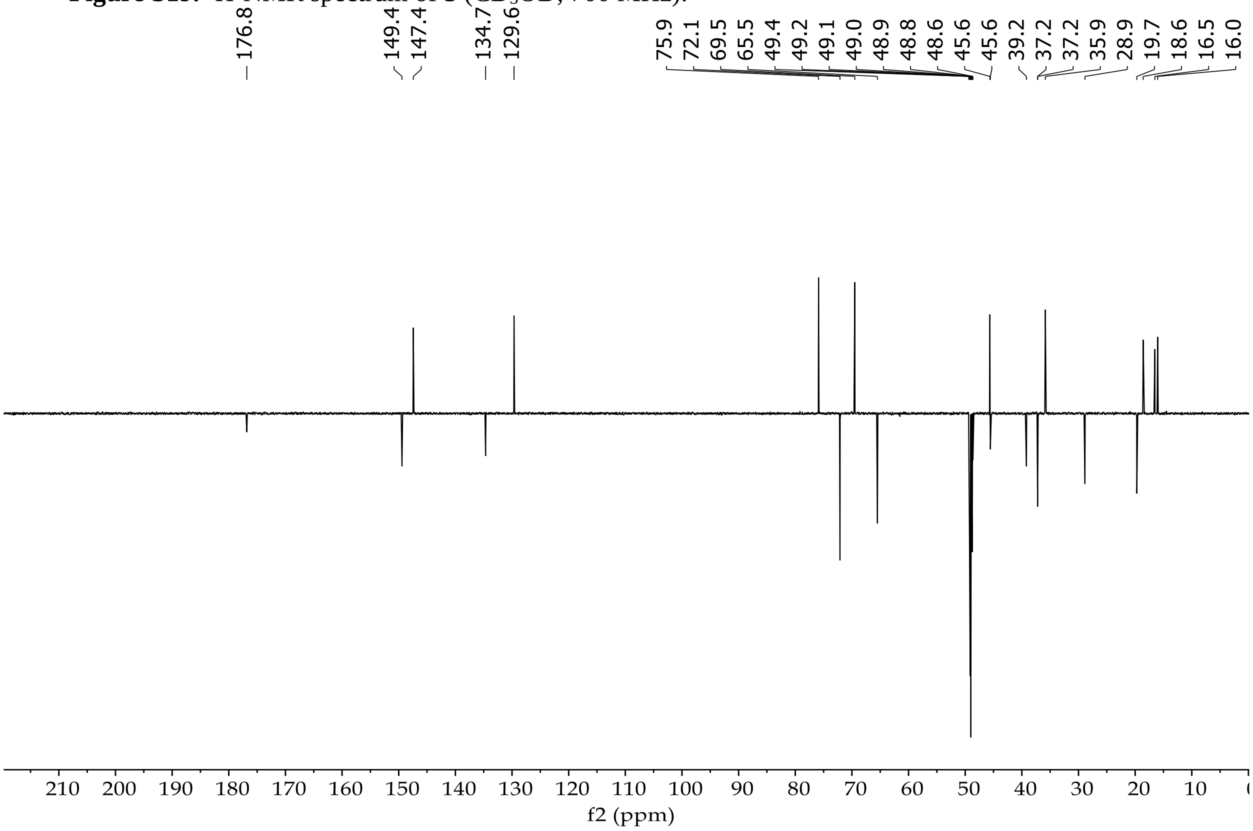

Figure S20. APT spectrum of **3** (CD_3OD, 700 MHz).

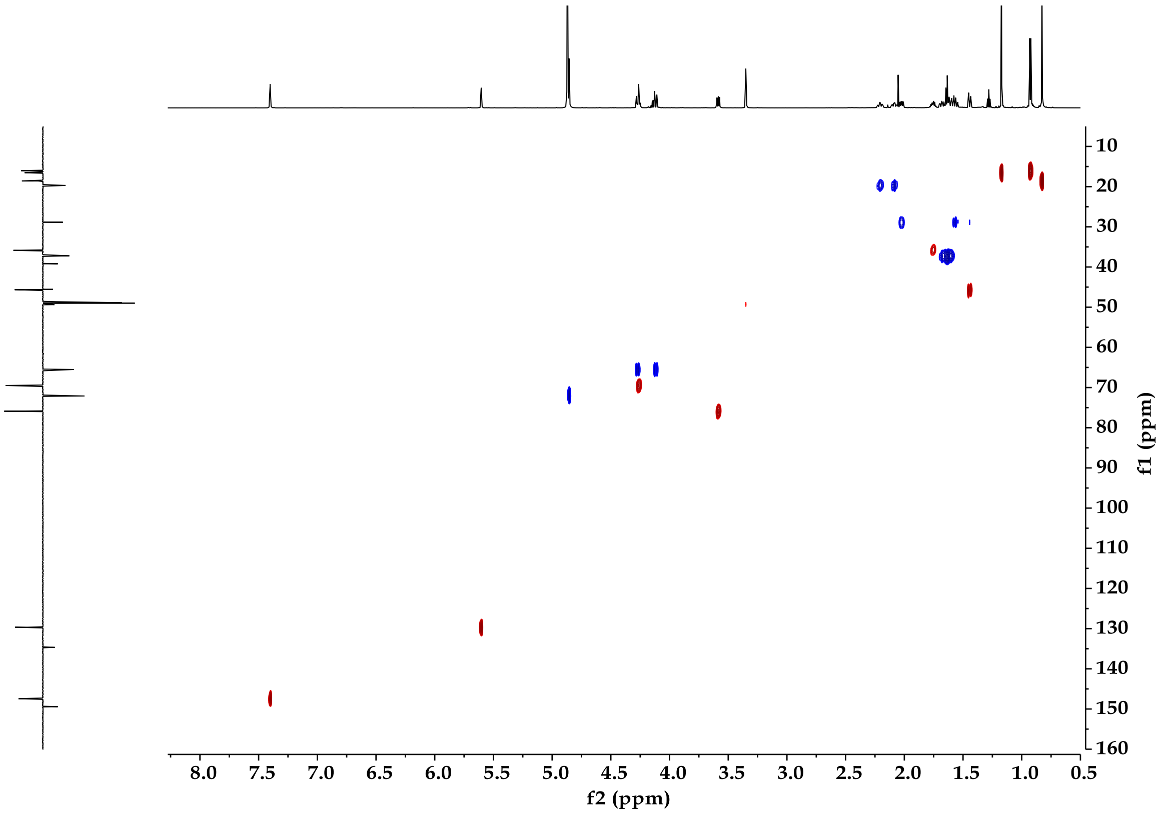

Figure S21. HSQC spectrum of **3** (CD_3OD, 700 MHz).

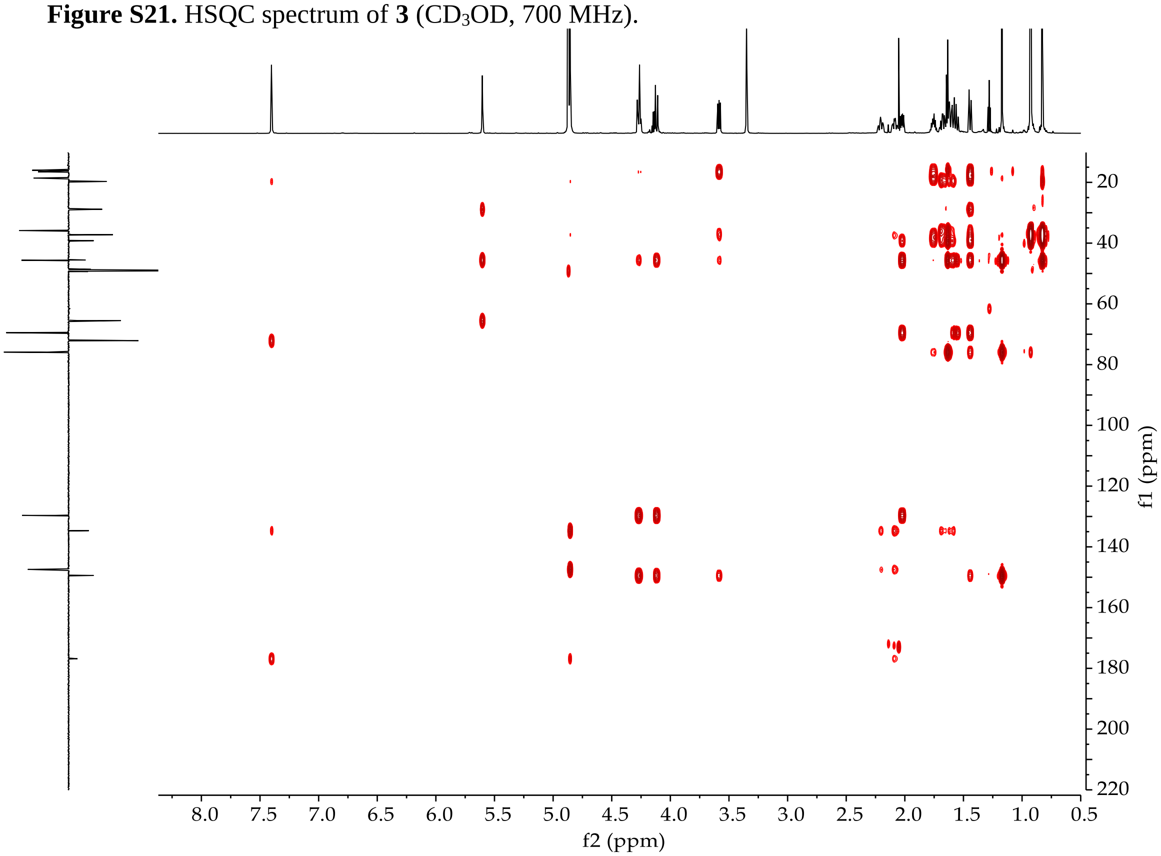

Figure S22. HMBC spectrum of **3** (CD_3OD, 700 MHz).

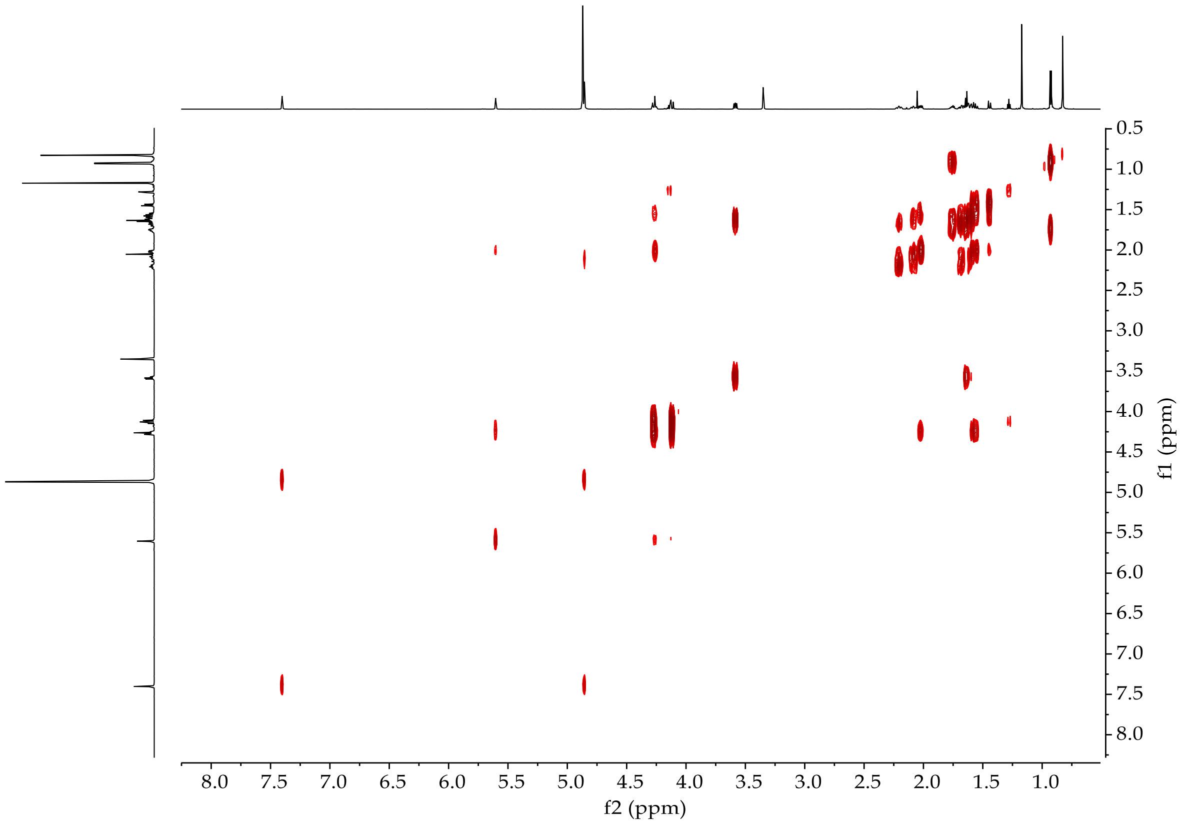

Figure S23. ^{1}H-^{1}H COSY spectrum of **3** (CD_3OD, 700 MHz).

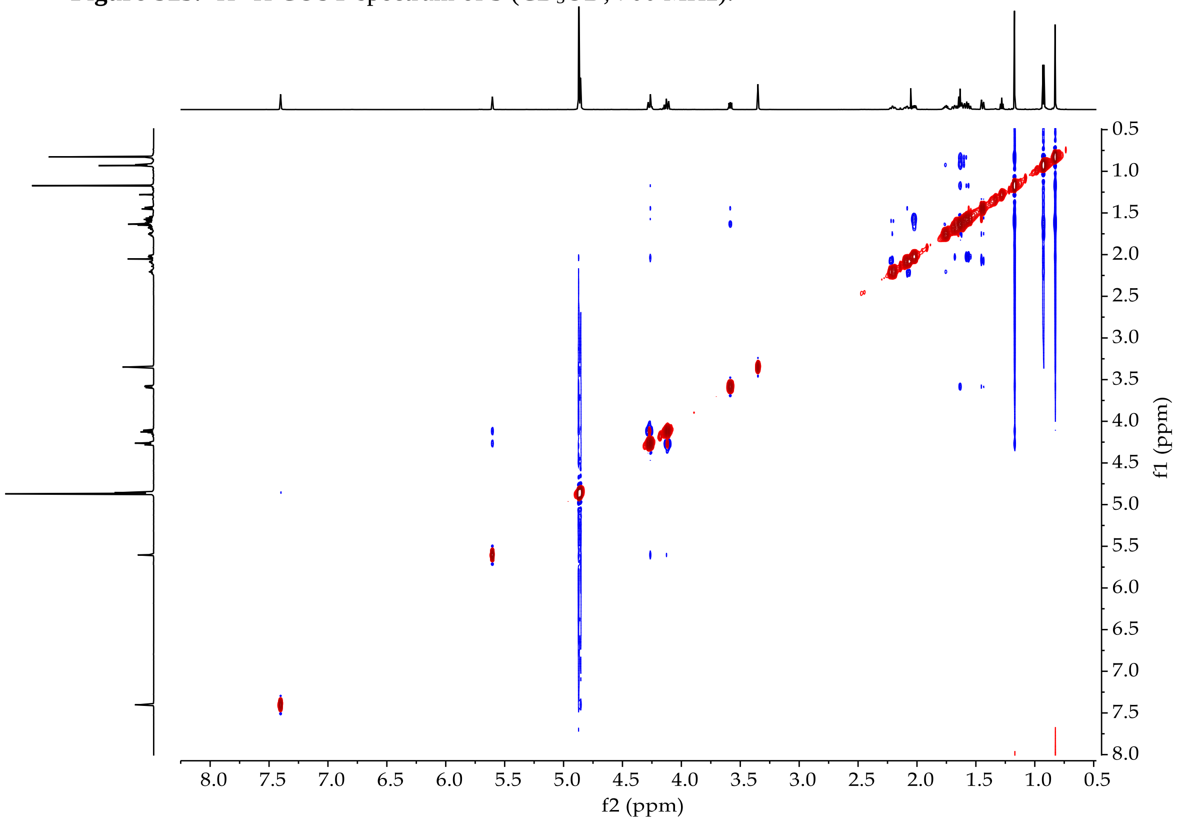

Figure S24. NOESY spectrum of **3** (CD_3OD, 700 MHz).

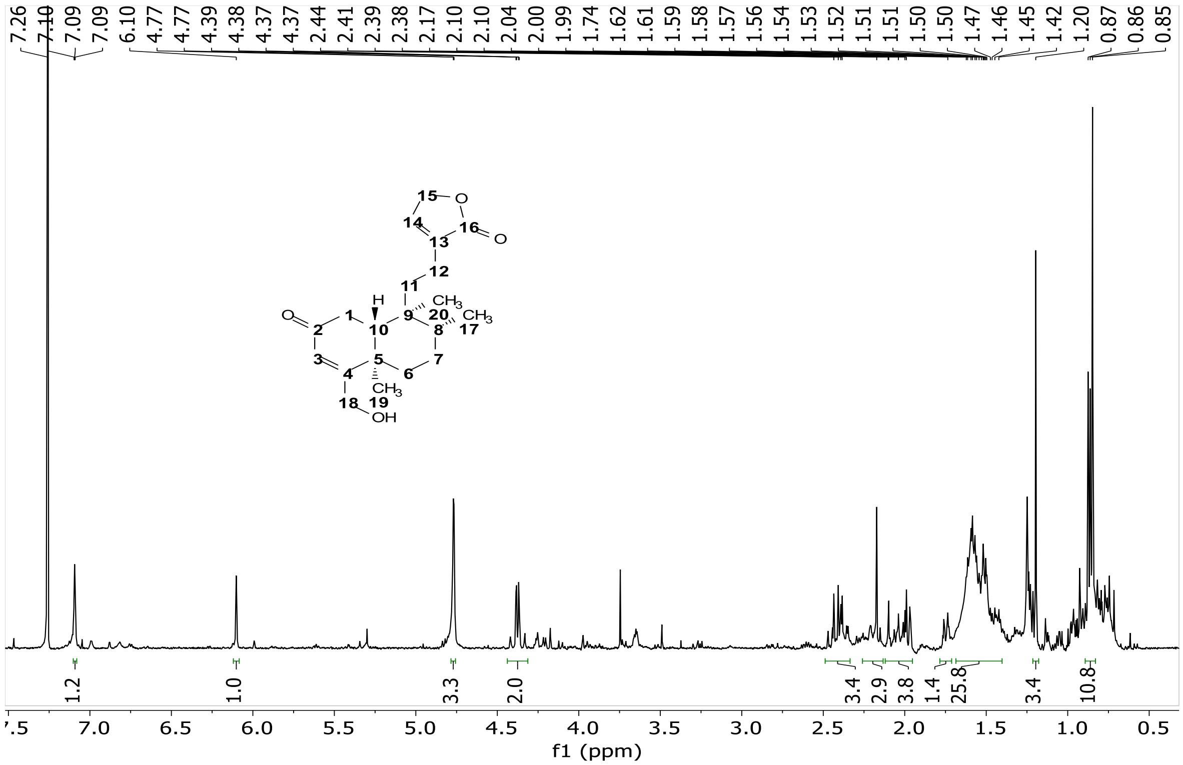

Figure S25. ¹H-NMR spectrum of **4** ($CDCl_3$, 700 MHz).

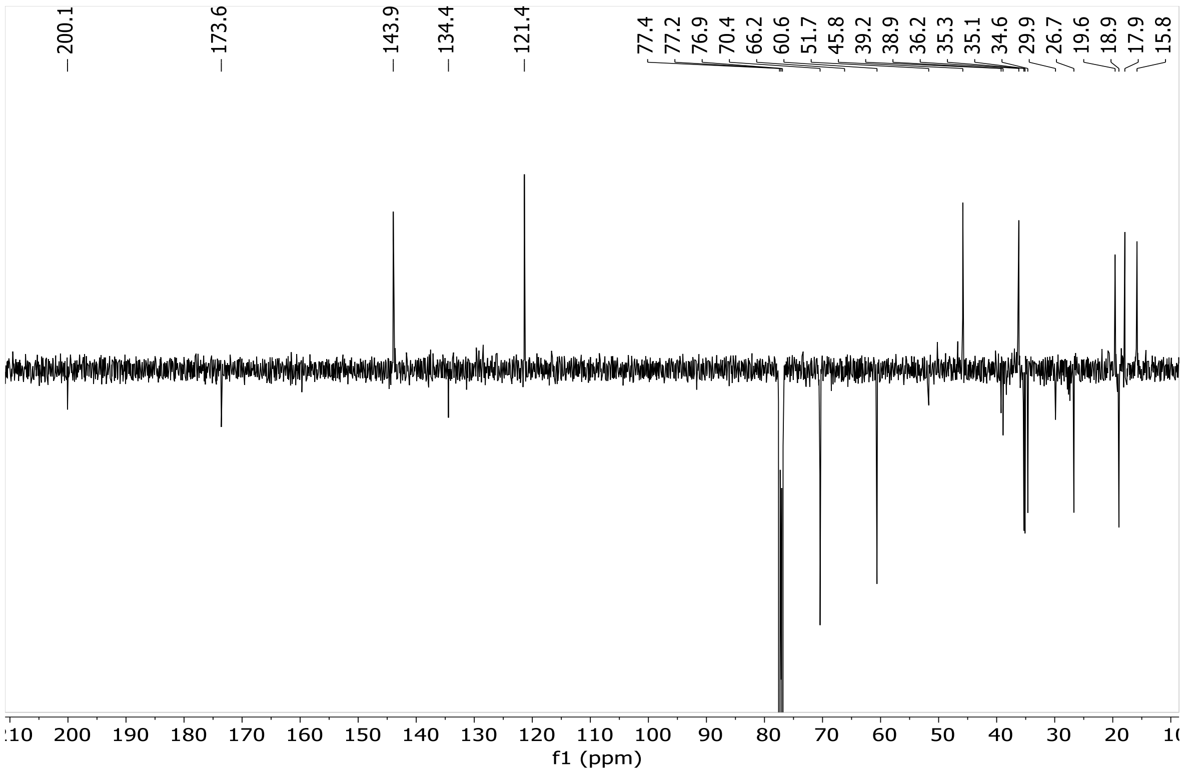

Figure S26. APT spectrum of **4** ($CDCl_3$, 175 MHz)

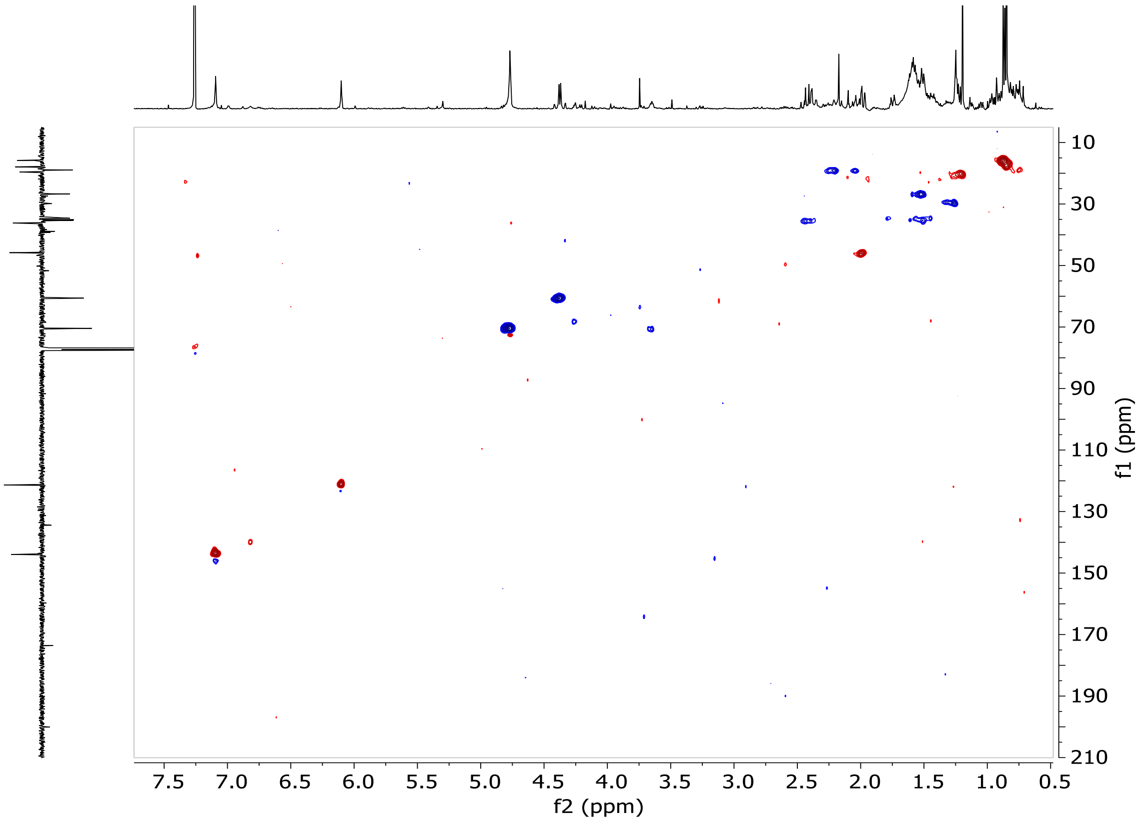

Figure S27. HSQC spectrum of **4** ($CDCl_3$, 700 MHz).

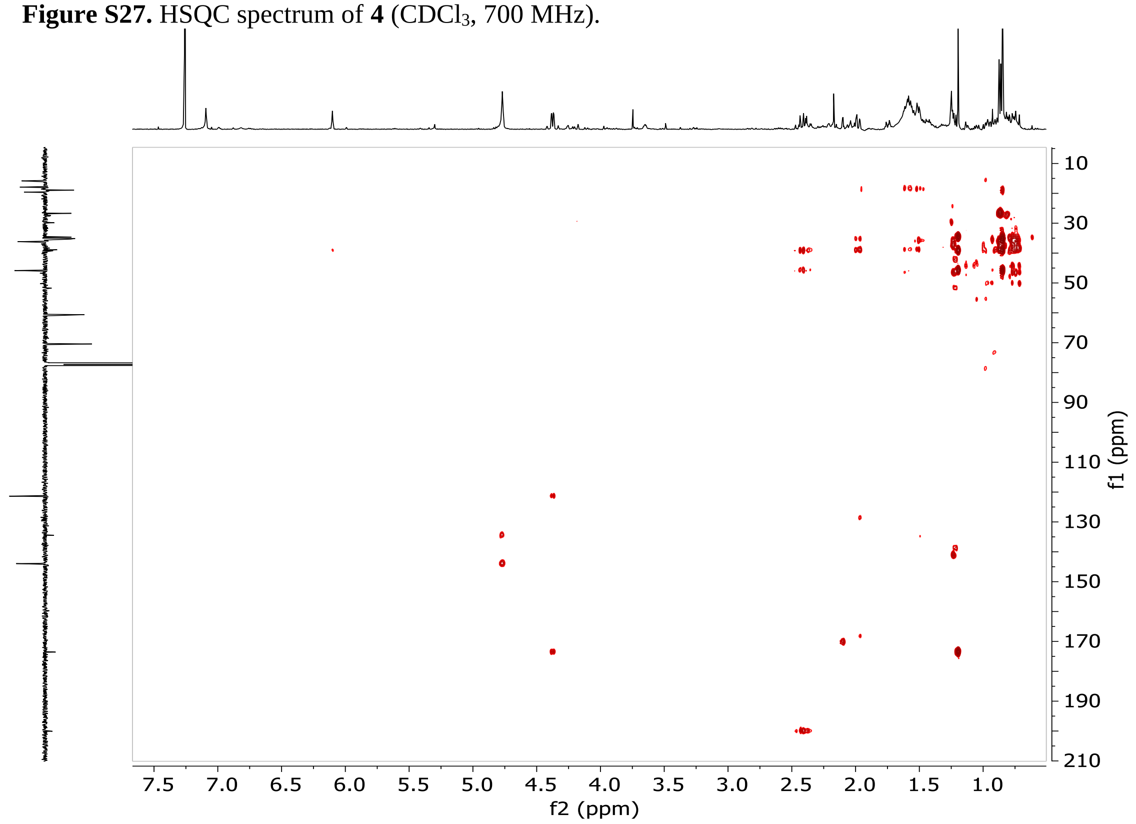

Figure S28. HMBC spectrum of **4** ($CDCl_3$, 700 MHz).

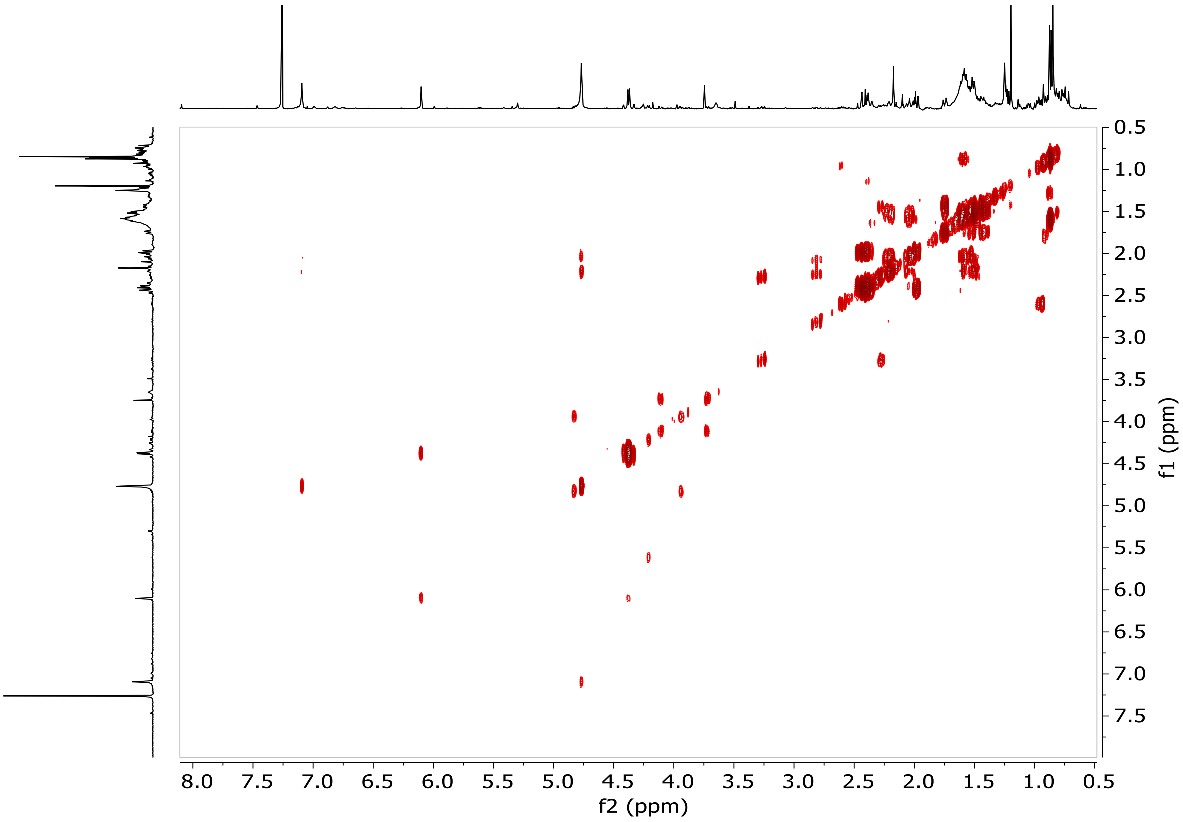

Figure S29. ^{1}H-^{1}H COSY spectrum of **4** ($CDCl_3$, 700 MHz).

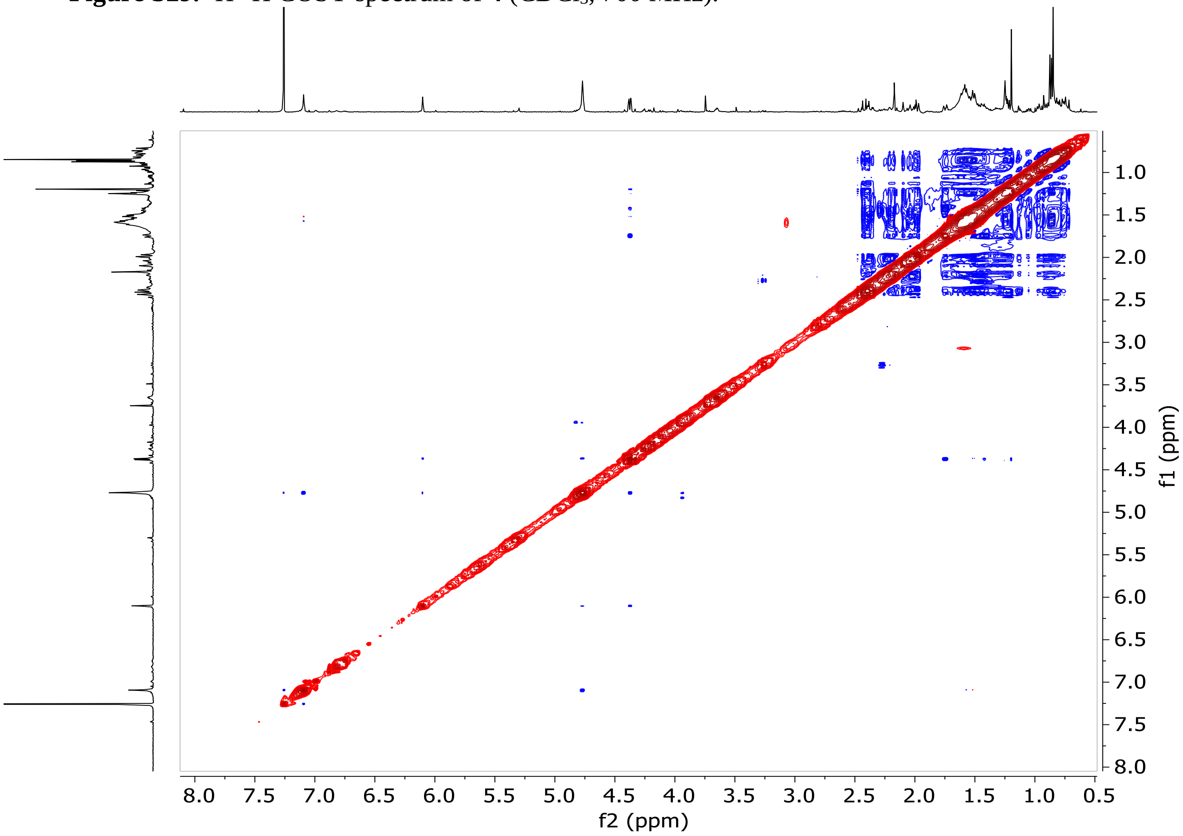

Figure S30. NOESY spectrum of **4** ($CDCl_3$, 700 MHz).

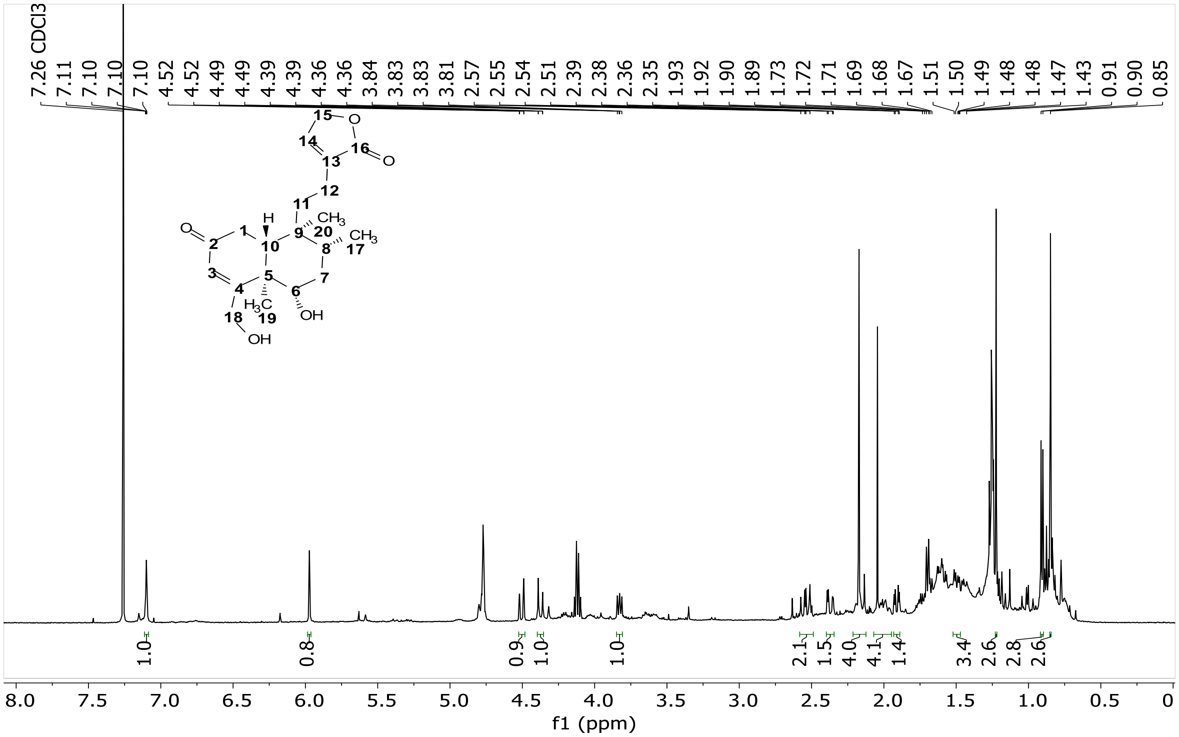

Figure S31. ^{1}H-NMR spectrum of **5** ($CDCl_3$, 700 MHz).

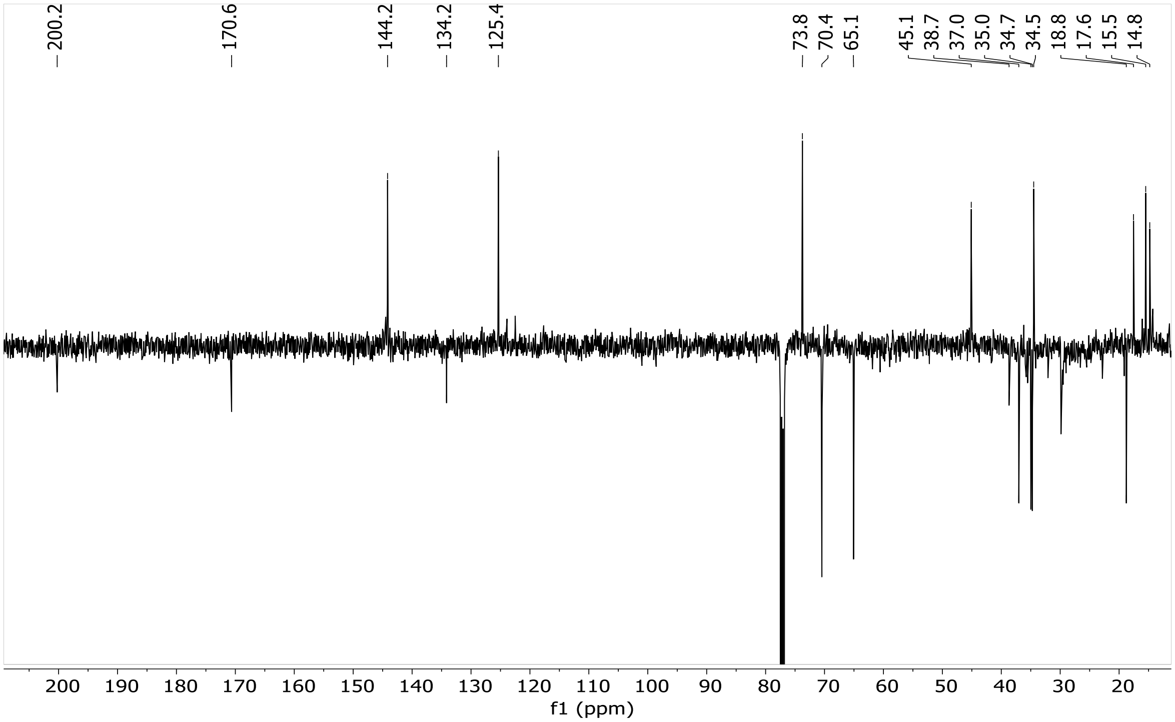

Figure S32. APT spectrum of **5** ($CDCl_3$, 700 MHz).

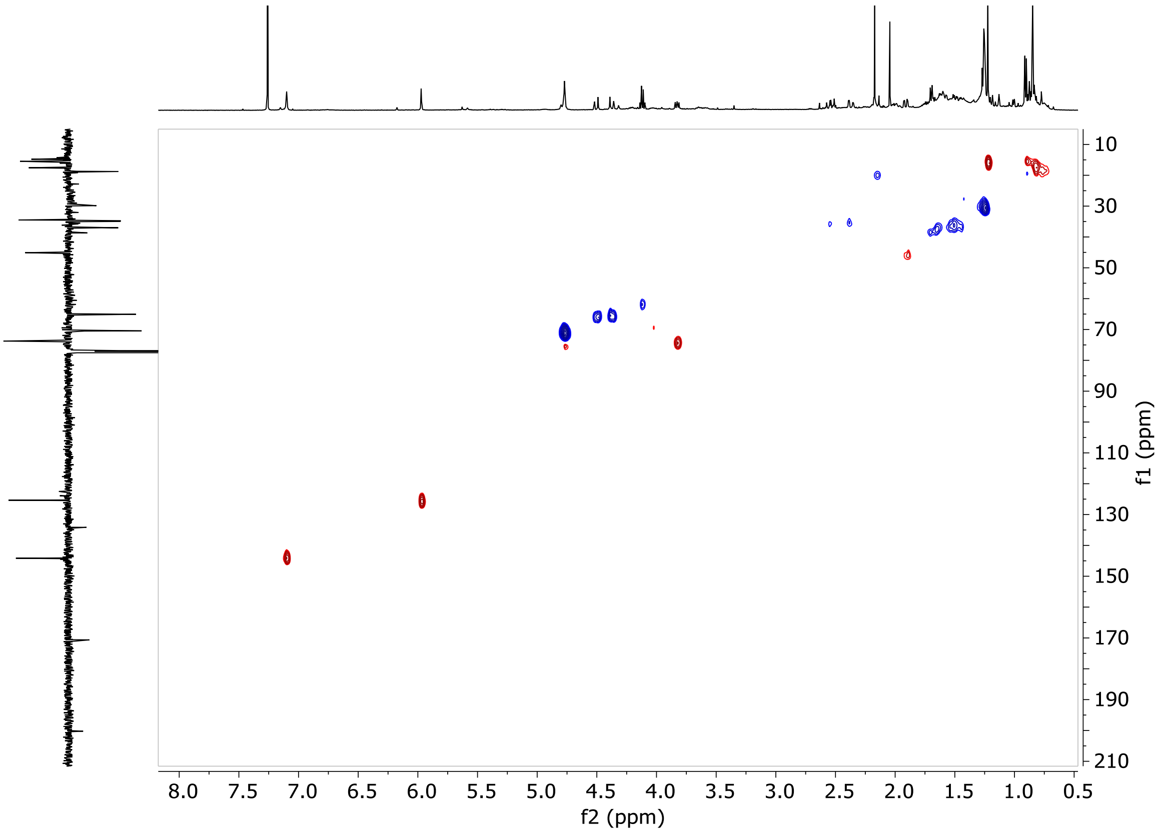

Figure S33. HSQC spectrum of **5** ($CDCl_3$, 700 MHz).

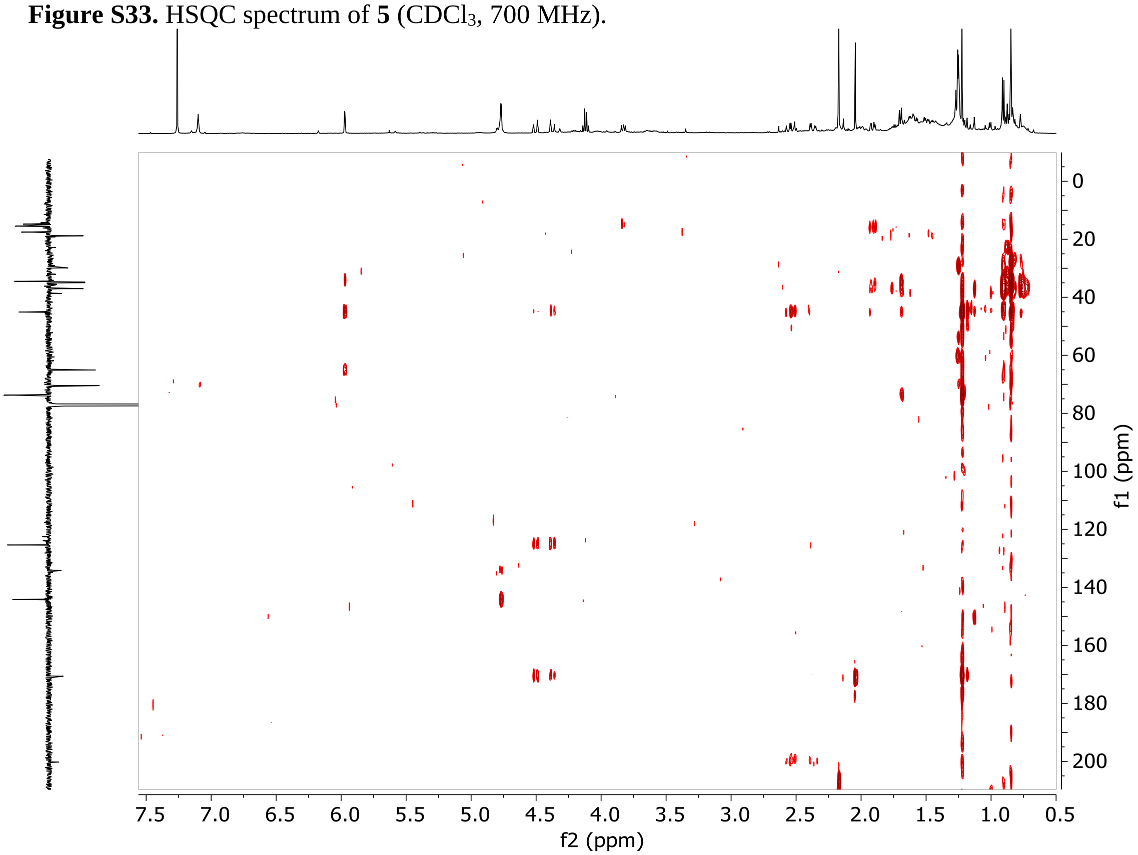

Figure S34. HMBC spectrum of **5** ($CDCl_3$, 700 MHz).

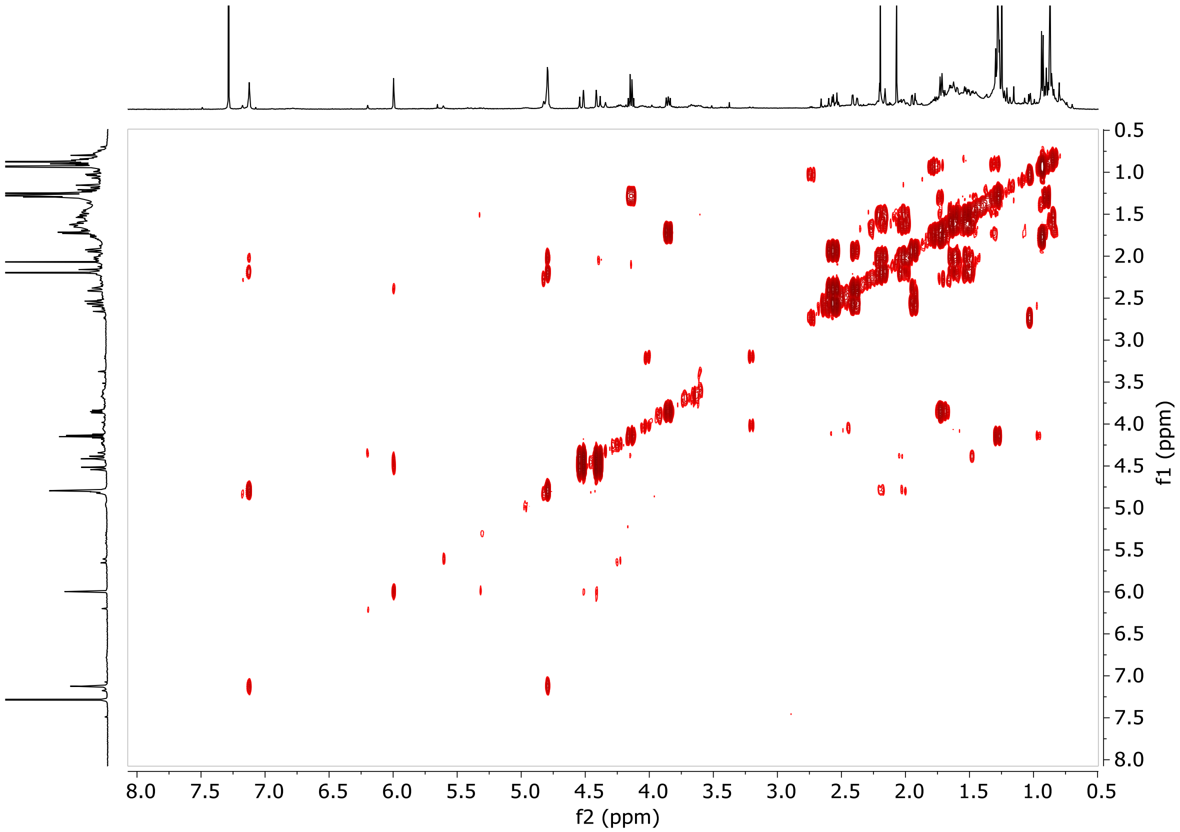

Figure S35. ^{1}H-^{1}H COSY spectrum of **5** ($CDCl_3$, 700 MHz).

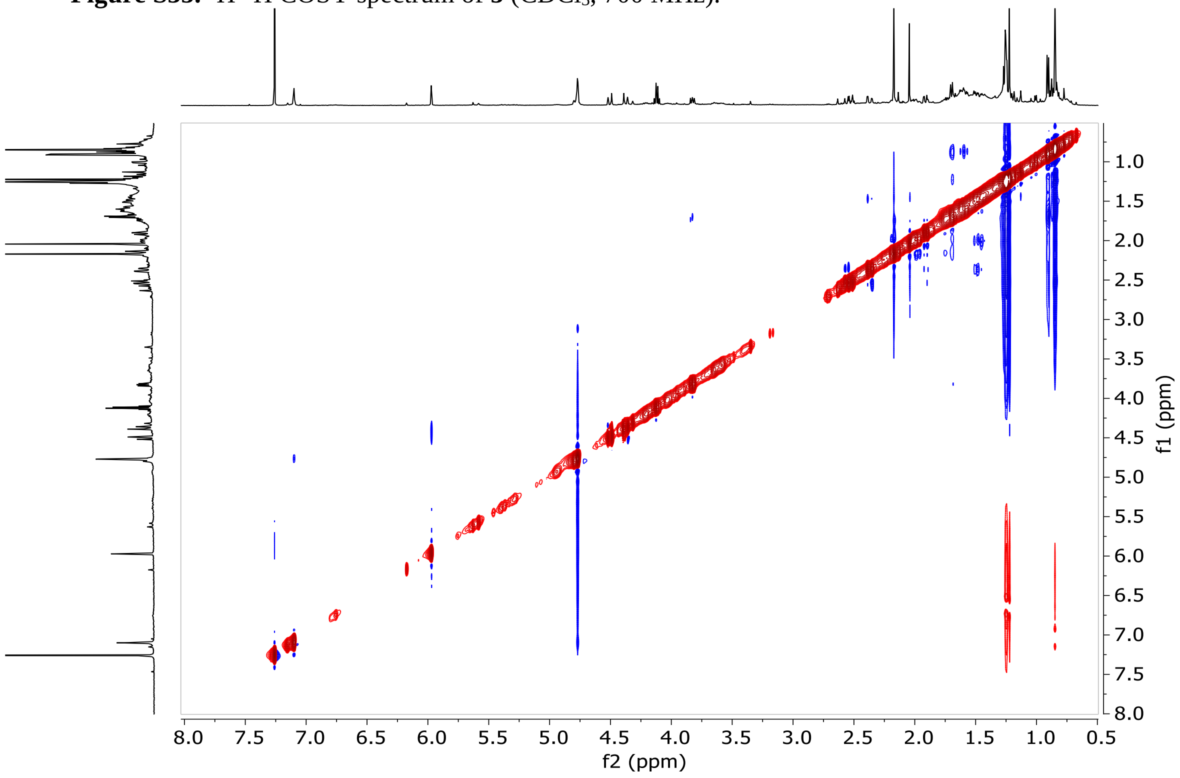

Figure S36. NOESY spectrum of **5** ($CDCl_3$, 700 MHz).

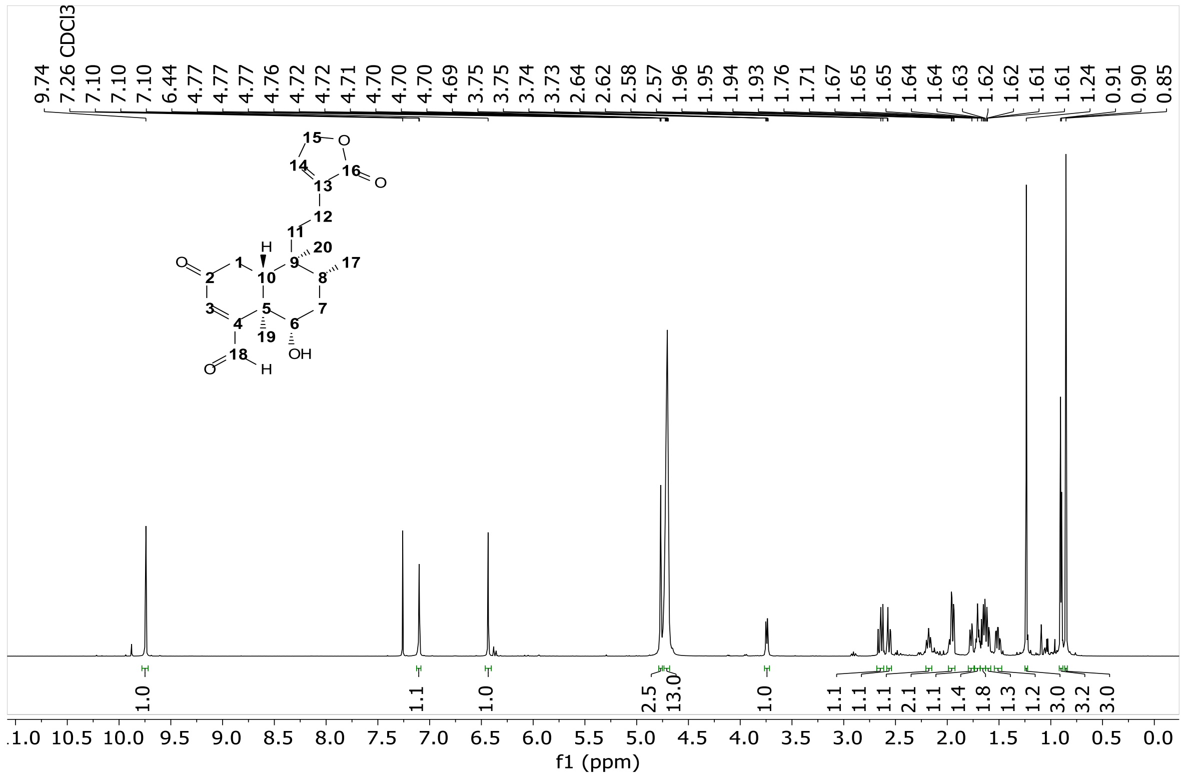

Figure S37. ^{1}H-NMR spectrum of **6** ($CDCl_3$, 700 MHz).

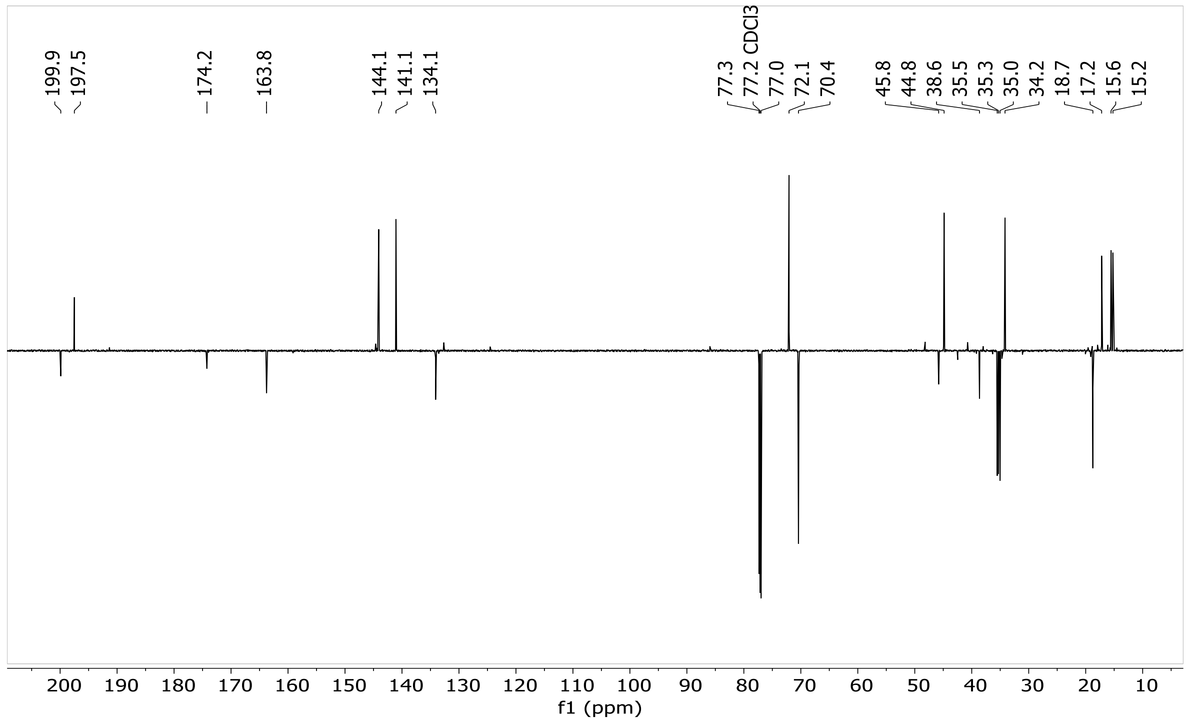

Figure S38. APT spectrum of **6** ($CDCl_3$, 175 MHz)

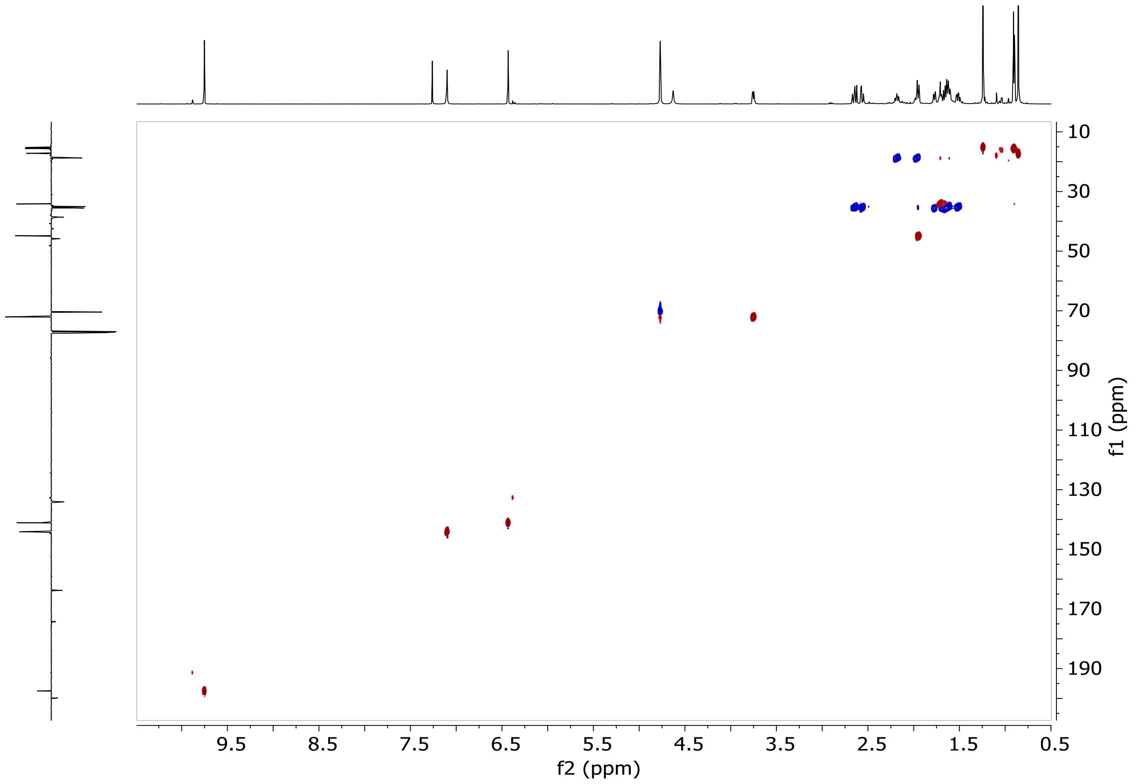

Figure S39. HSQC spectrum of **6** ($CDCl_3$, 700 MHz).

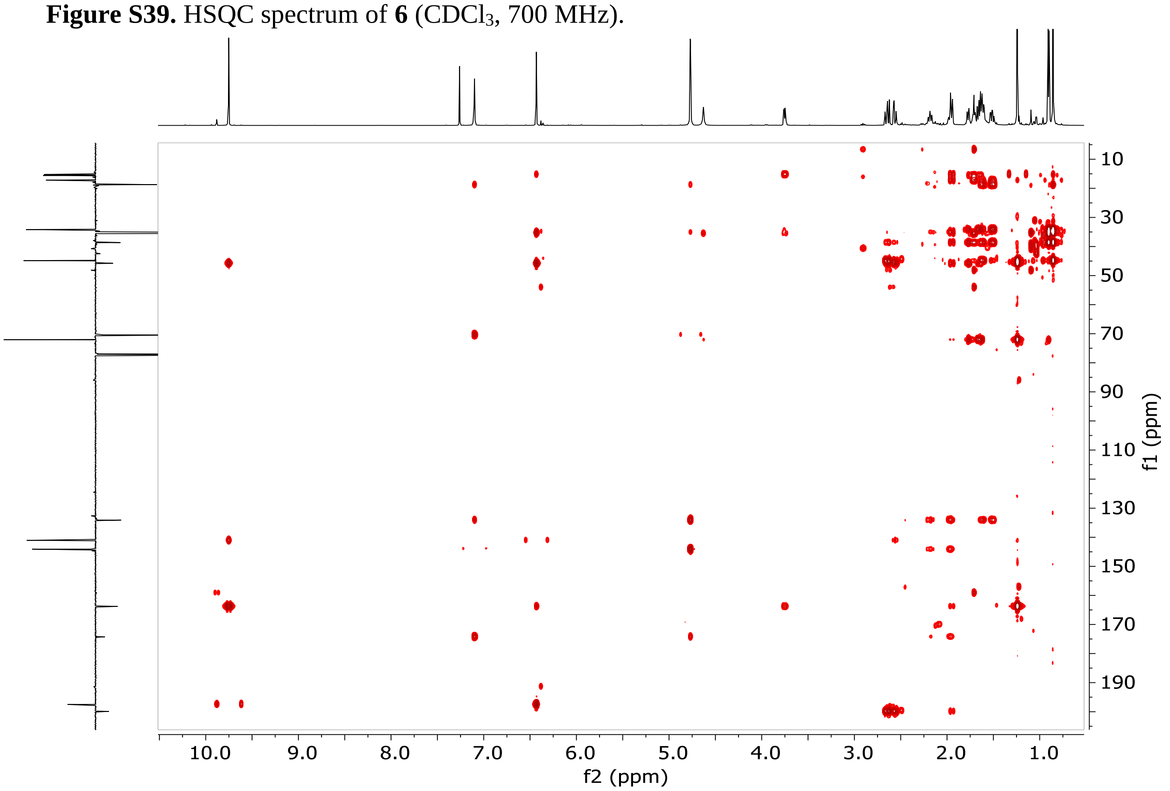

Figure S40. HMBC spectrum of **6** ($CDCl_3$, 700 MHz)

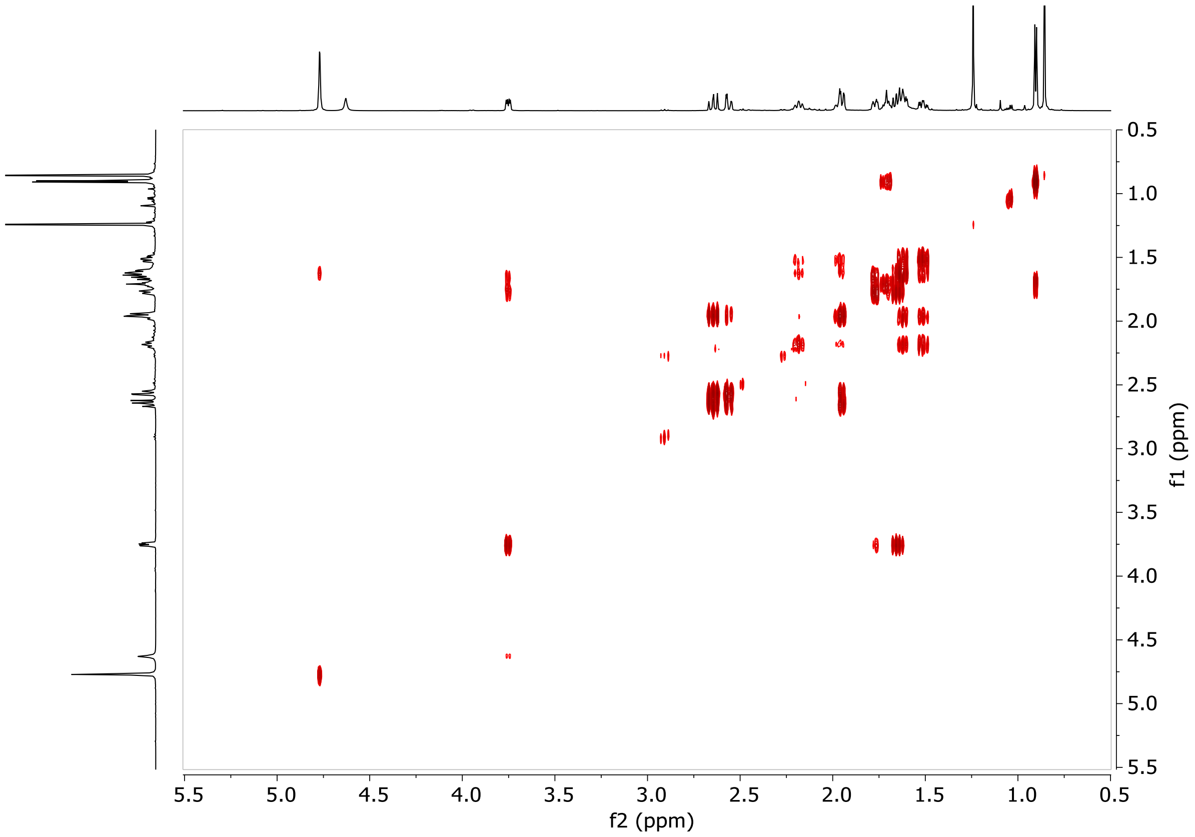

Figure S41. ^{1}H-^{1}H COSY spectrum of **6** ($CDCl_3$, 700 MHz).

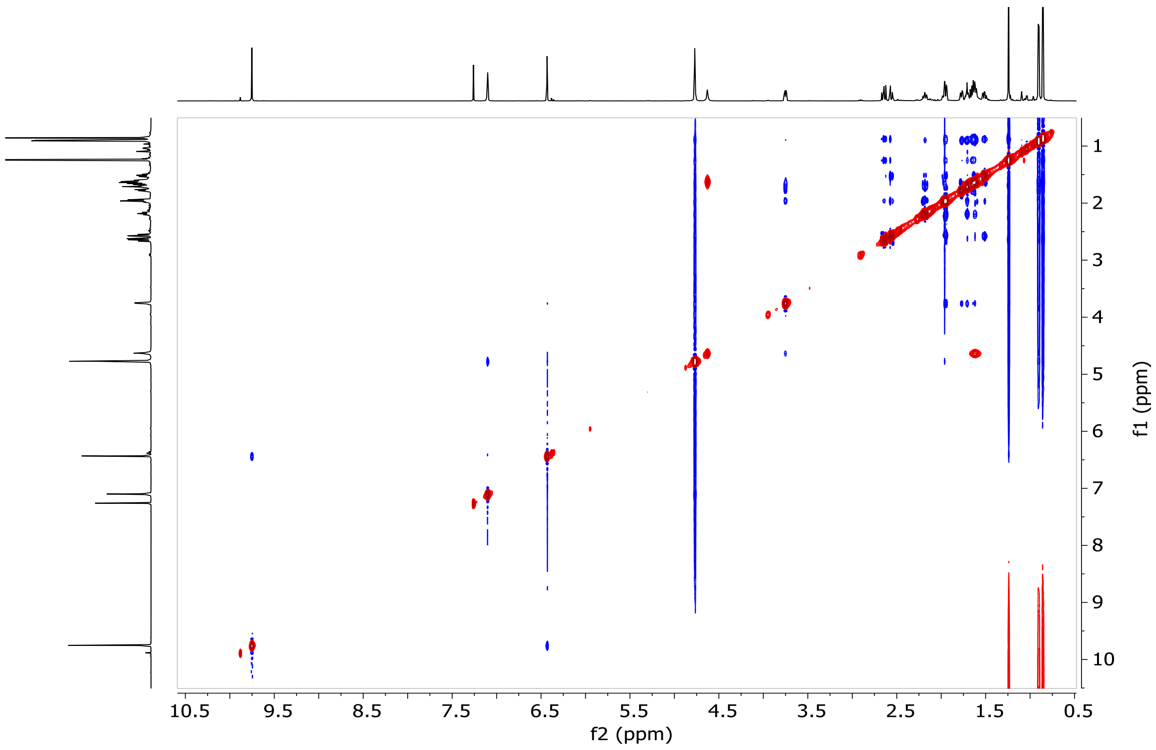

Figure S42. NOESY spectrum of **6** ($CDCl_3$, 700 MHz).

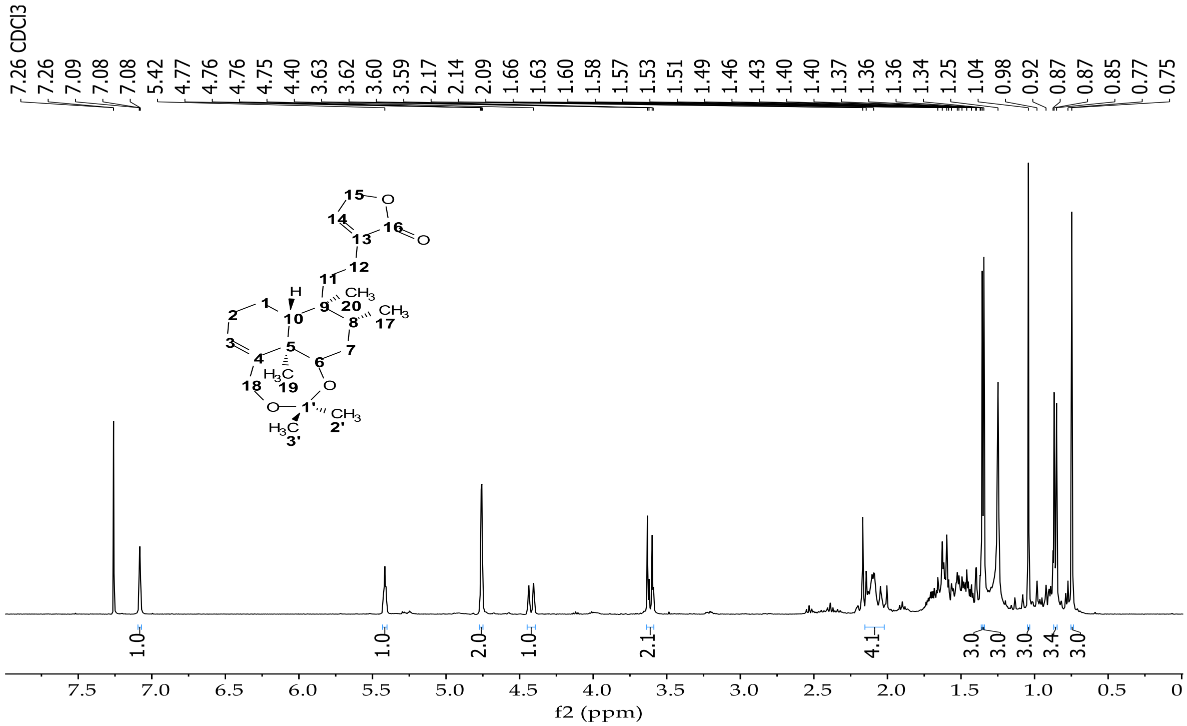

Figure S43. ^{1}H-NMR spectrum of **7** ($CDCl_3$, 400 MHz).

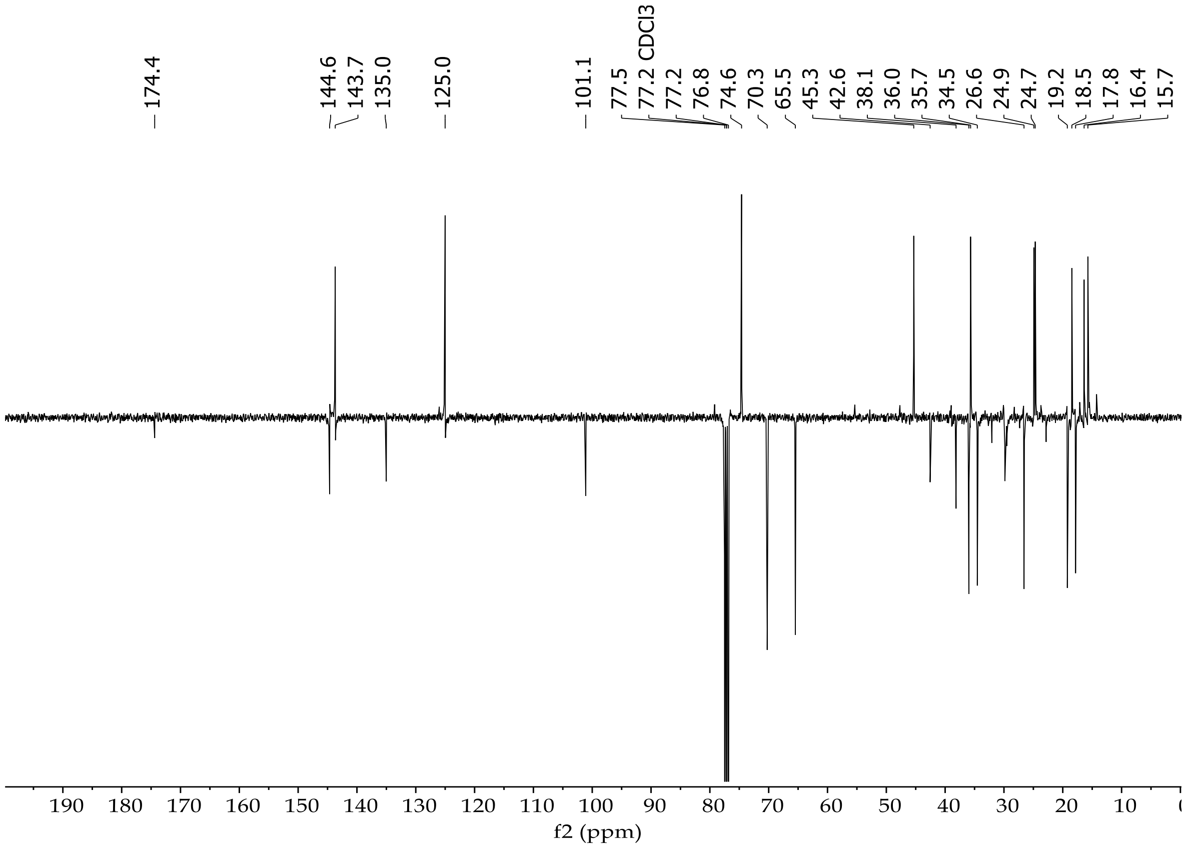

Figure S44. APT spectrum of **7** ($CDCl_3$, 400 MHz).

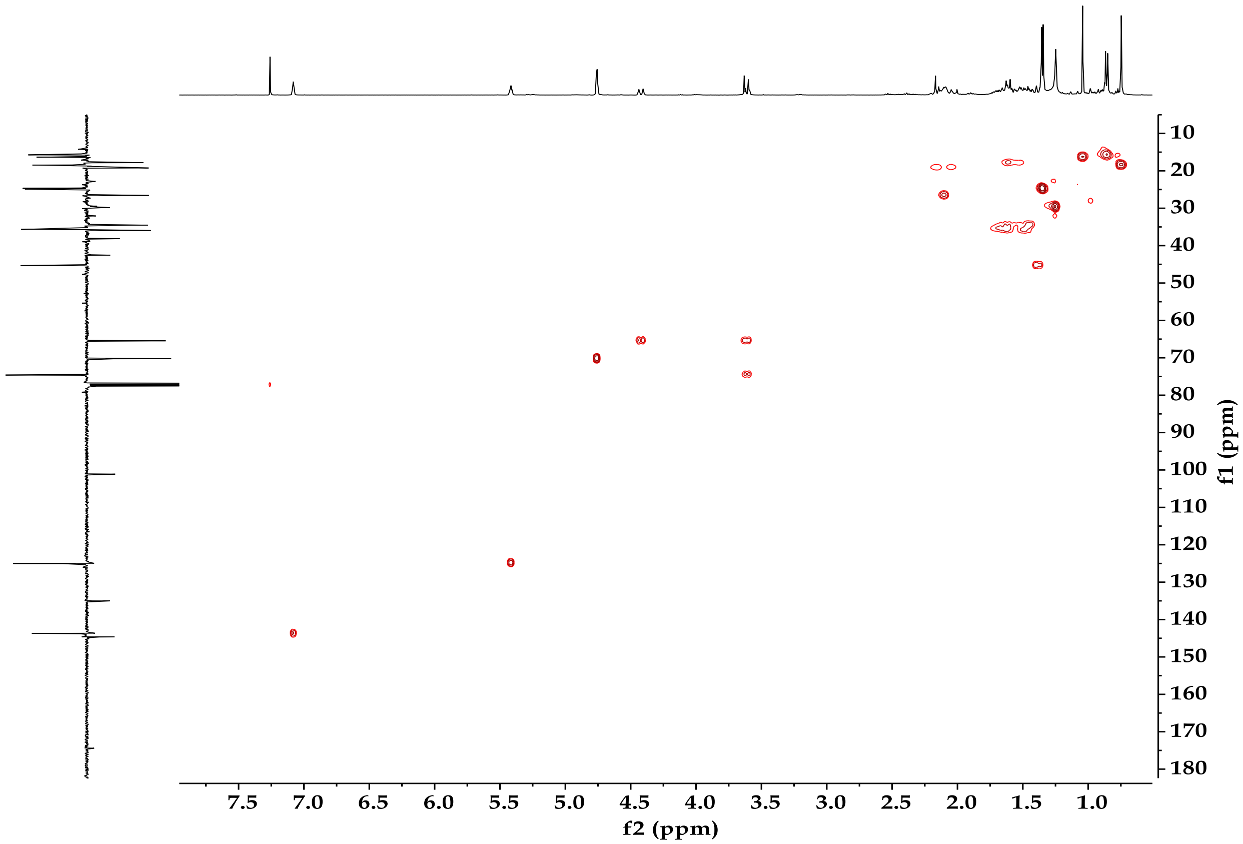

Figure S45. HSQC spectrum of **7** ($CDCl_3$, 400 MHz).

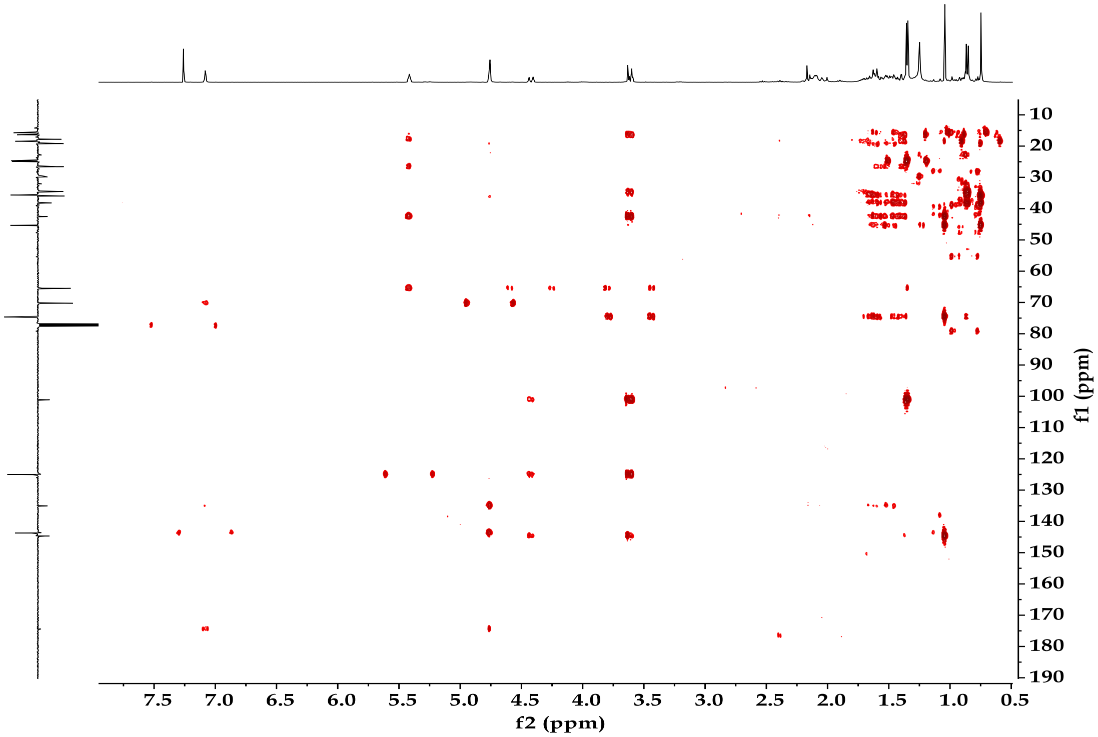

Figure S46. HMBC spectrum of **7** ($CDCl_3$,400 MHz).

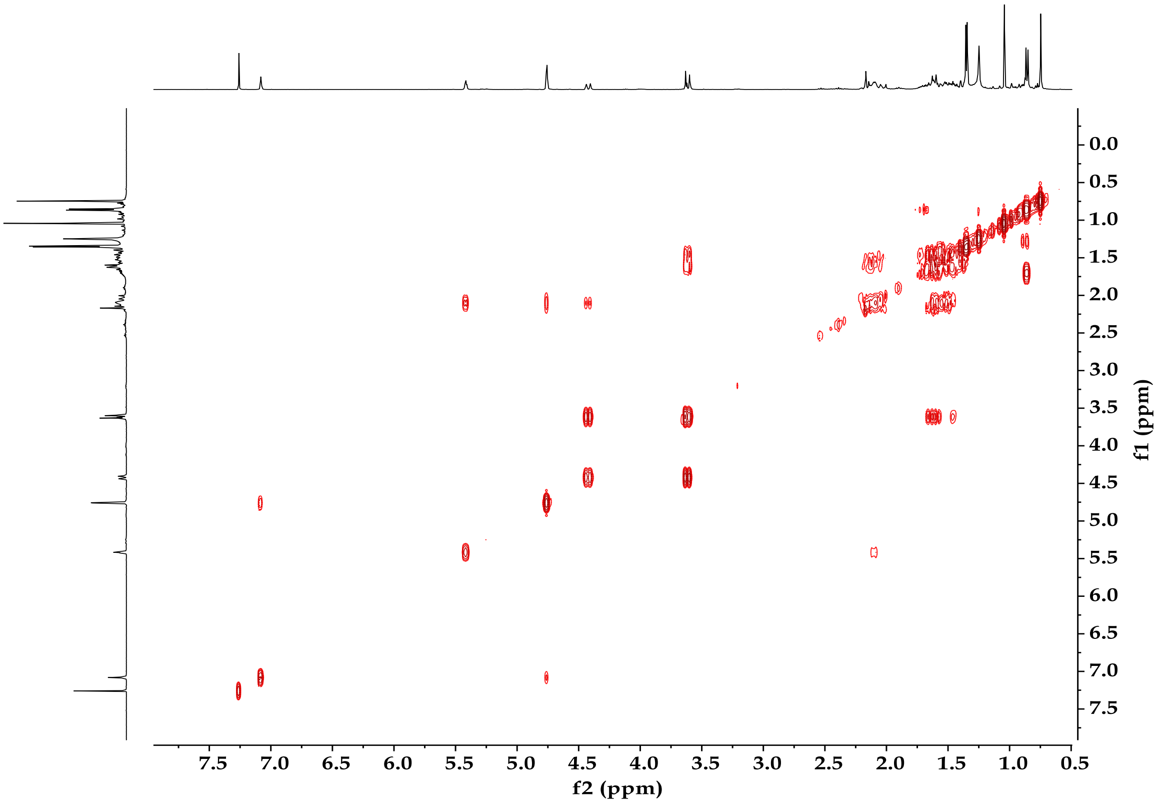

Figure S47. ^{1}H-^{1}H COSY spectrum of **7** ($CDCl_3$, 400 MHz).

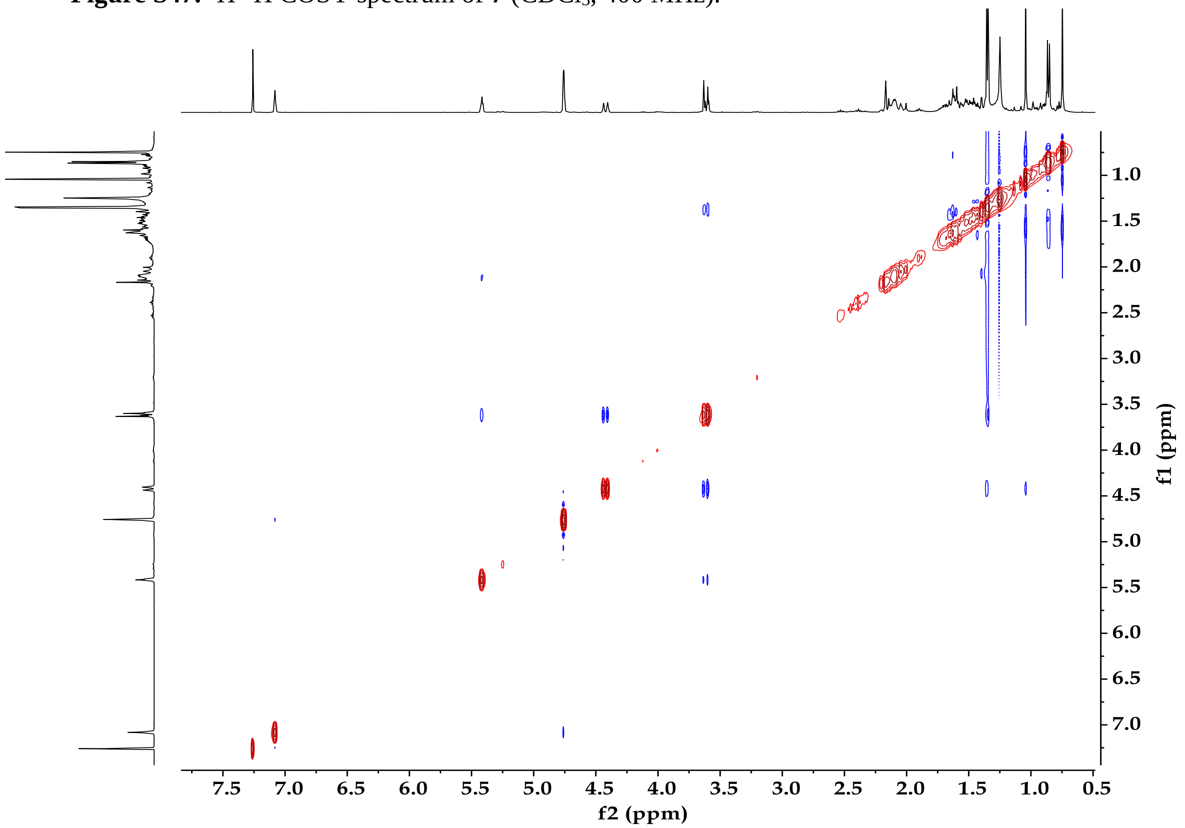

Figure S48. NOESY spectrum of **7** ($CDCl_3$, 400 MHz).

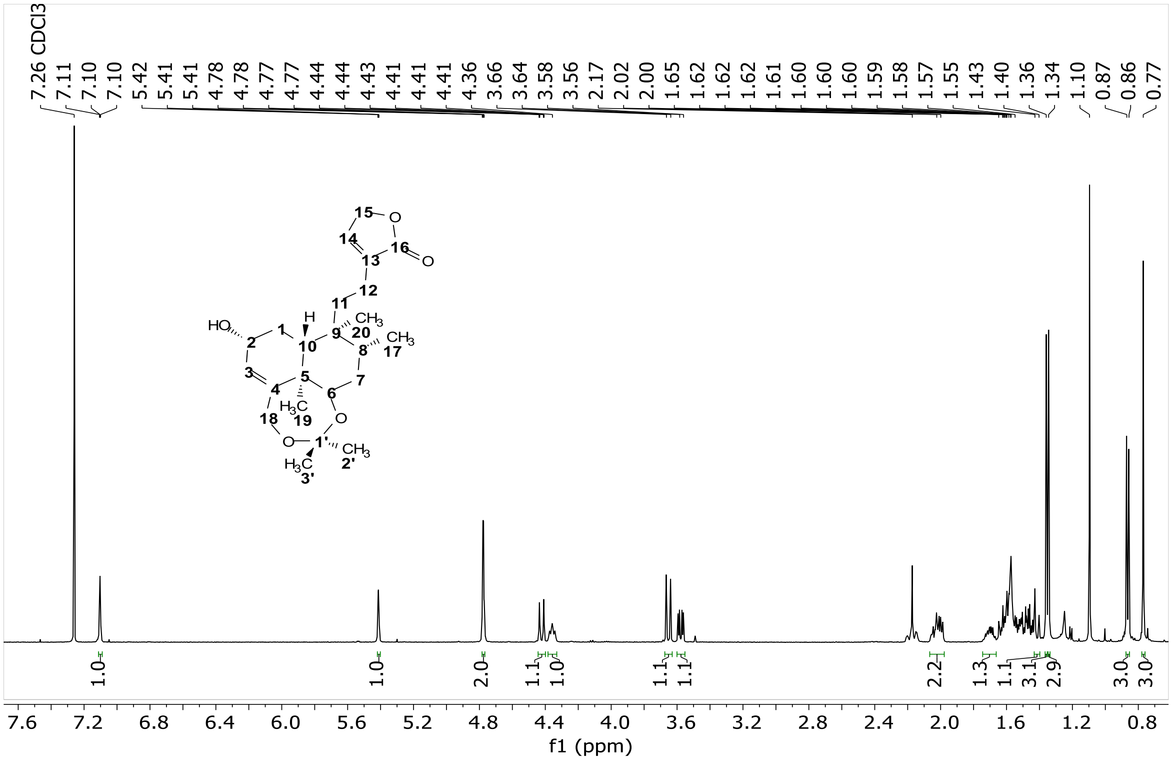

Figure S49. ^{1}H-NMR spectrum of **8** ($CDCl_3$, 700 MHz).

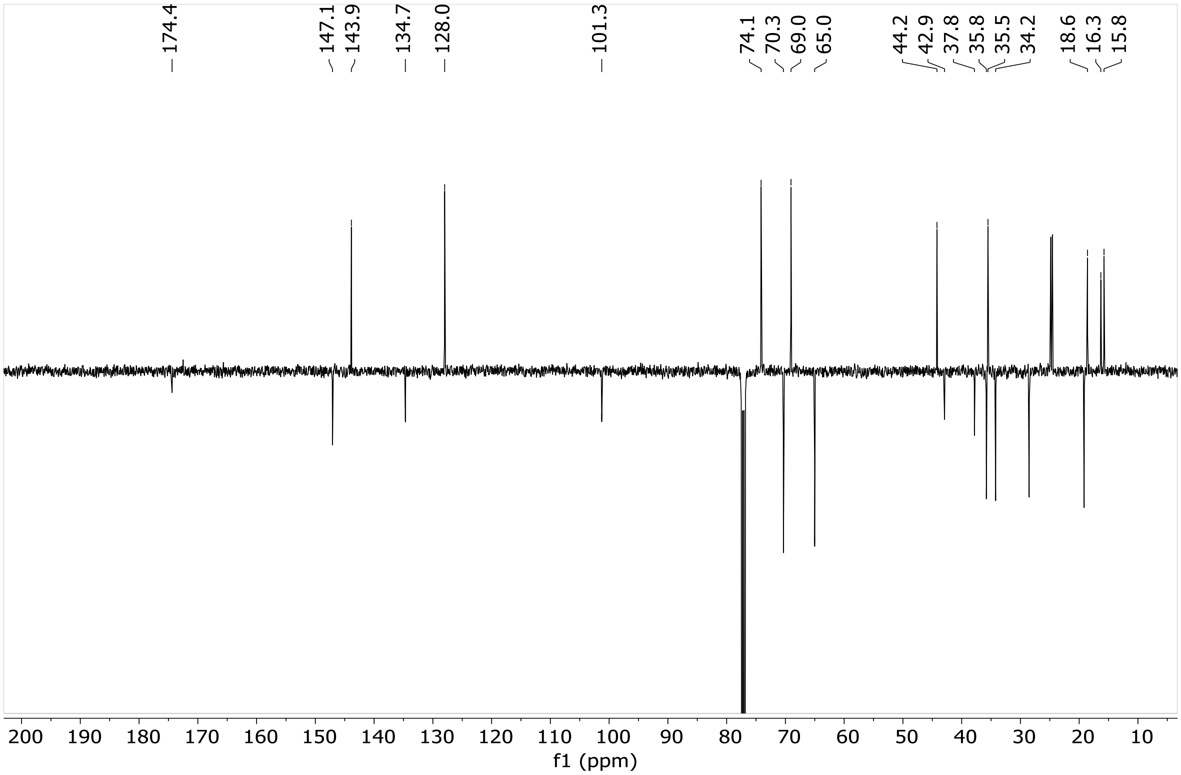

Figure S50. APT spectrum of **8** ($CDCl_3$, 700 MHz).

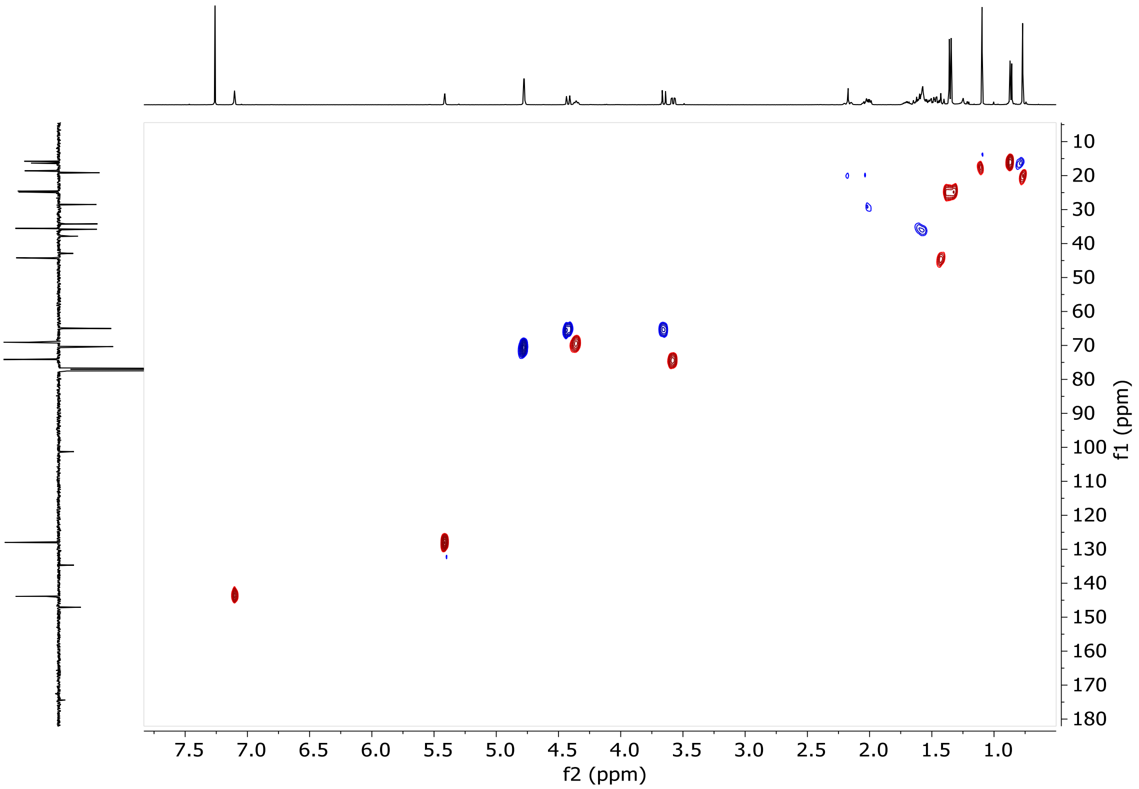

Figure S51. HSQC spectrum of **8** ($CDCl_3$, 700 MHz).

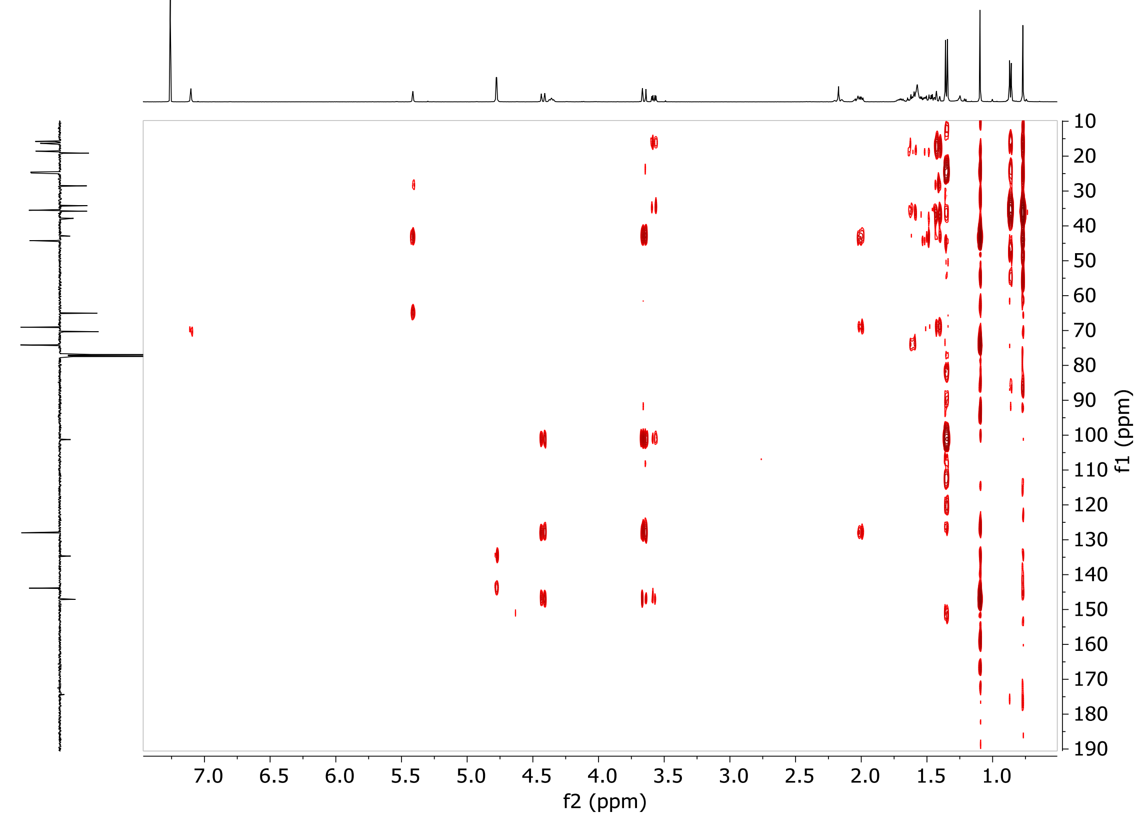

Figure S52. HMBC spectrum of **8** ($CDCl_3$, 700 MHz).

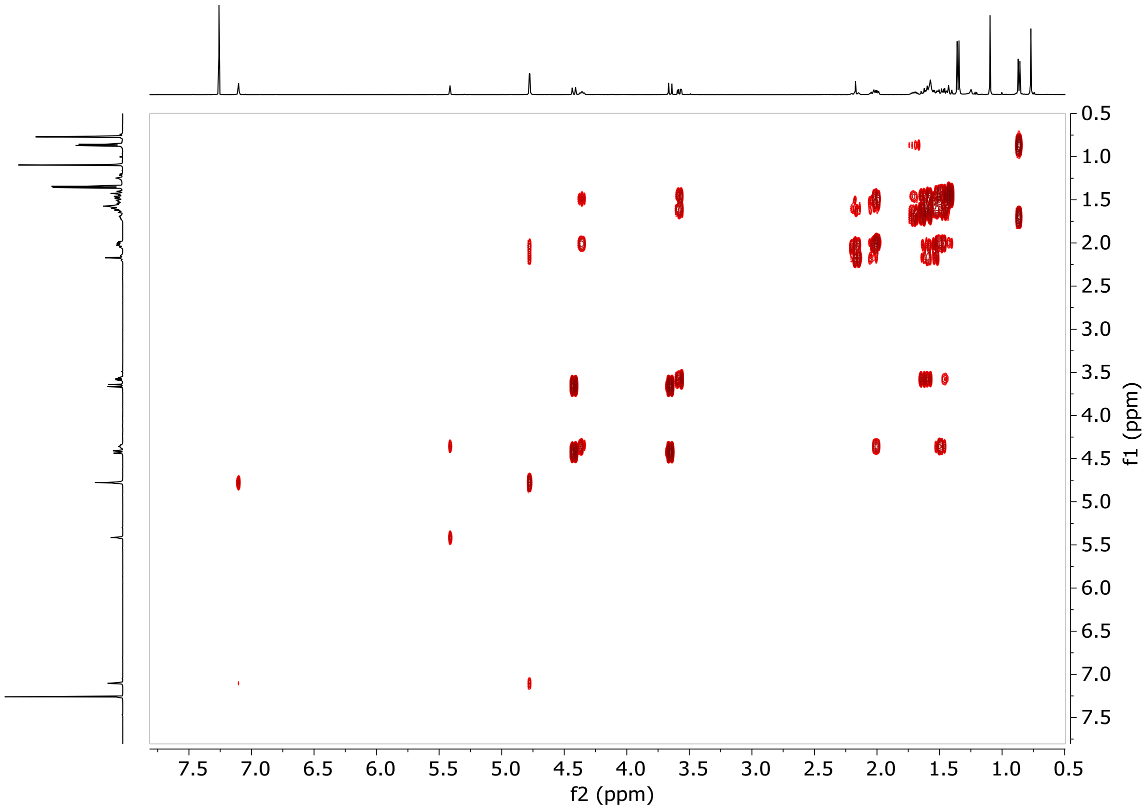

Figure S53. ^1H-^1H COSY spectrum of **8** ($CDCl_3$, 700 MHz).

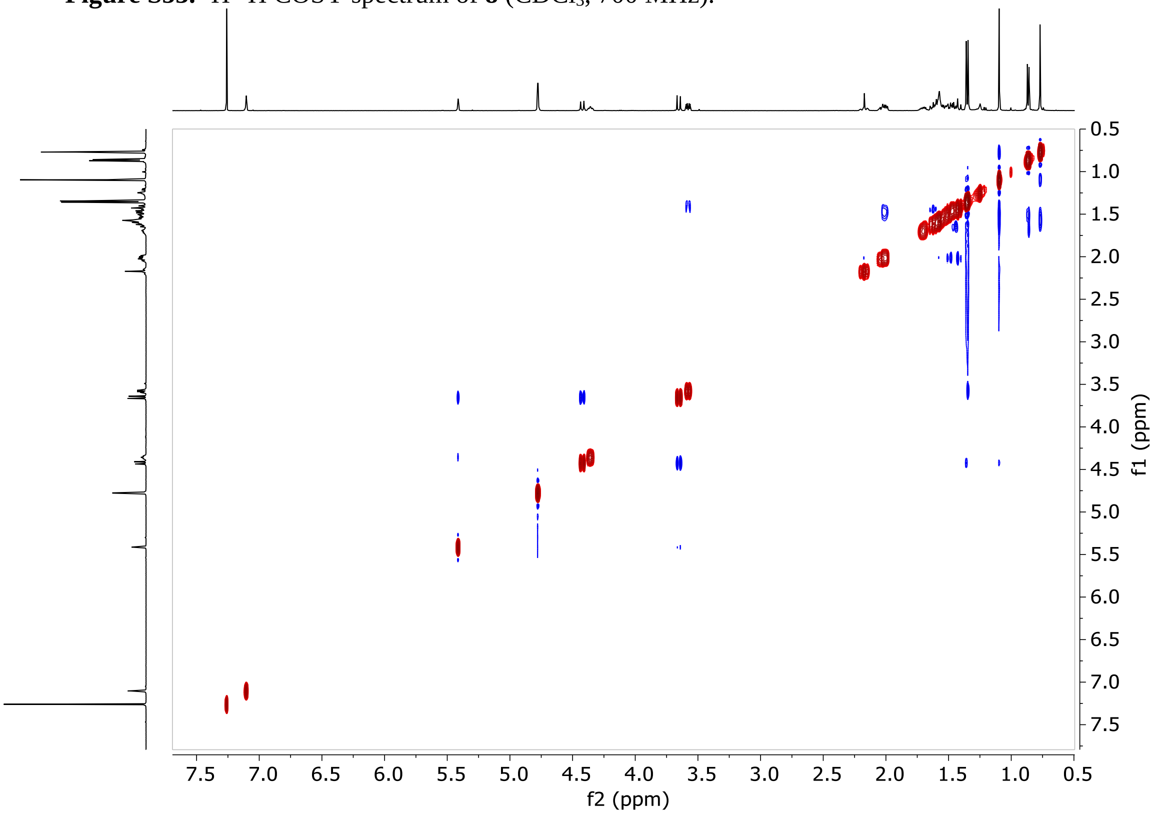

Figure S54. NOESY spectrum of **8** ($CDCl_3$, 700 MHz).

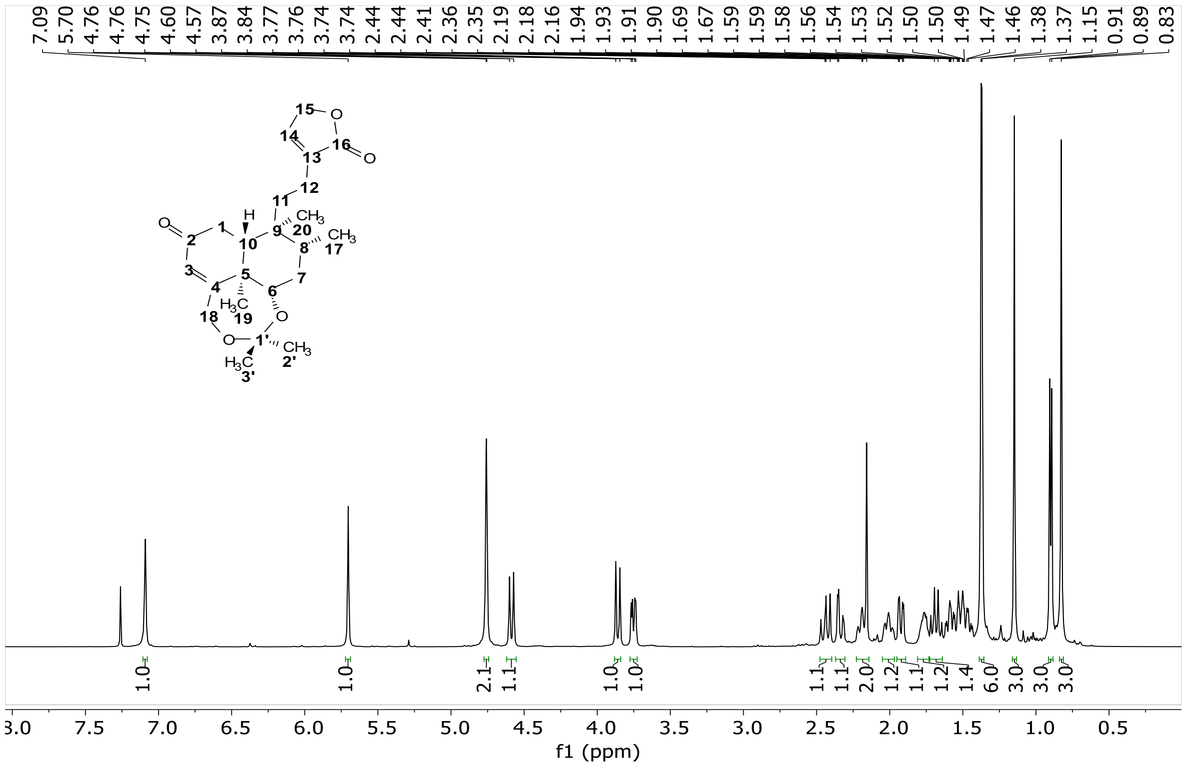

Figure S55. ^{1}H-NMR spectrum of **9** ($CDCl_3$, 700 MHz)

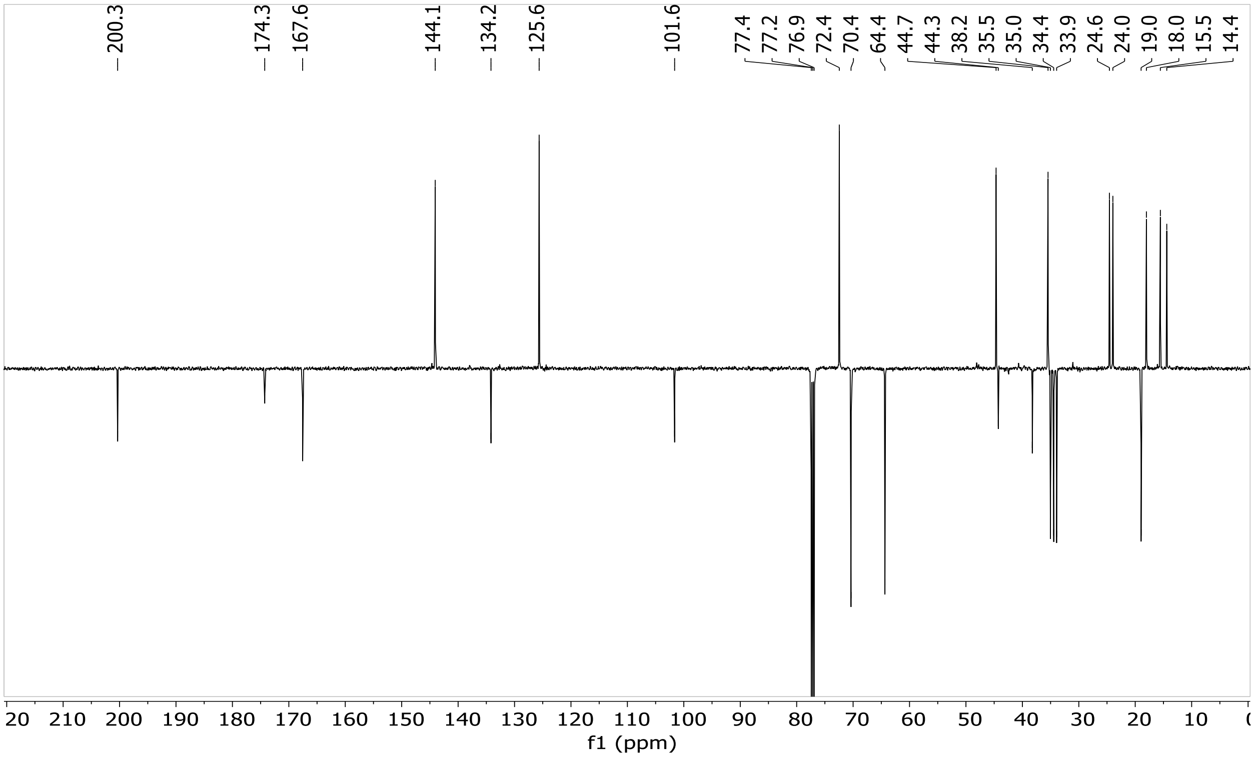

Figure S56. APT spectrum of **9** ($CDCl_3$,175 MHz).

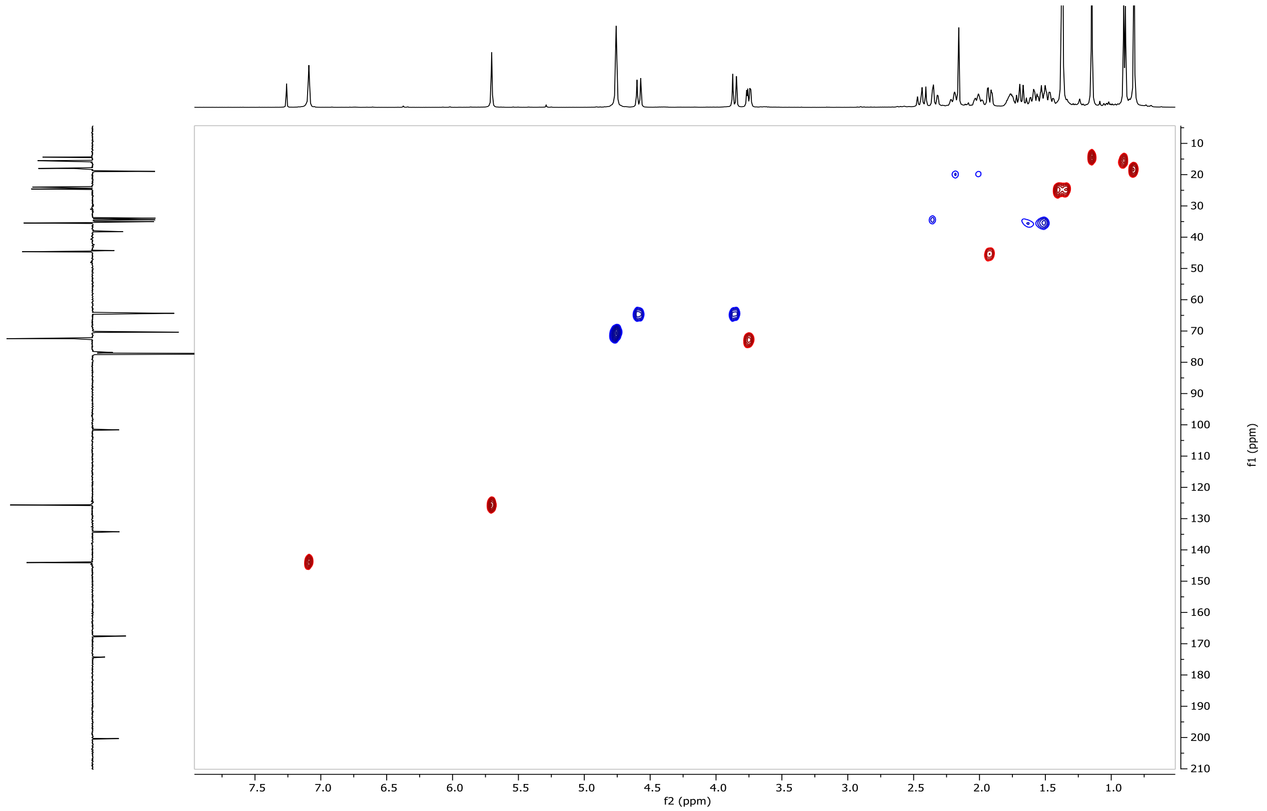

Figure S57. HSQC spectrum of **9** ($CDCl_3$, 700 MHz).

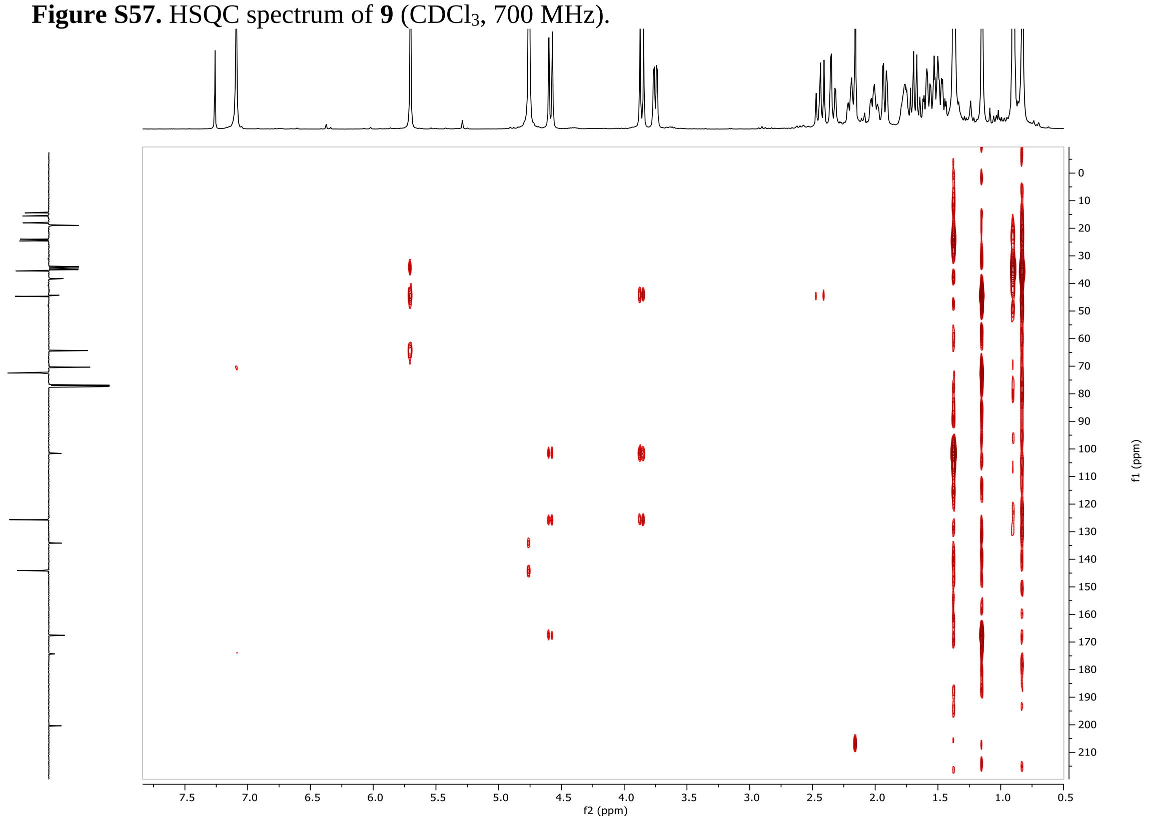

Figure S58. HMBC spectrum of **9** ($CDCl_3$, 700 MHz).

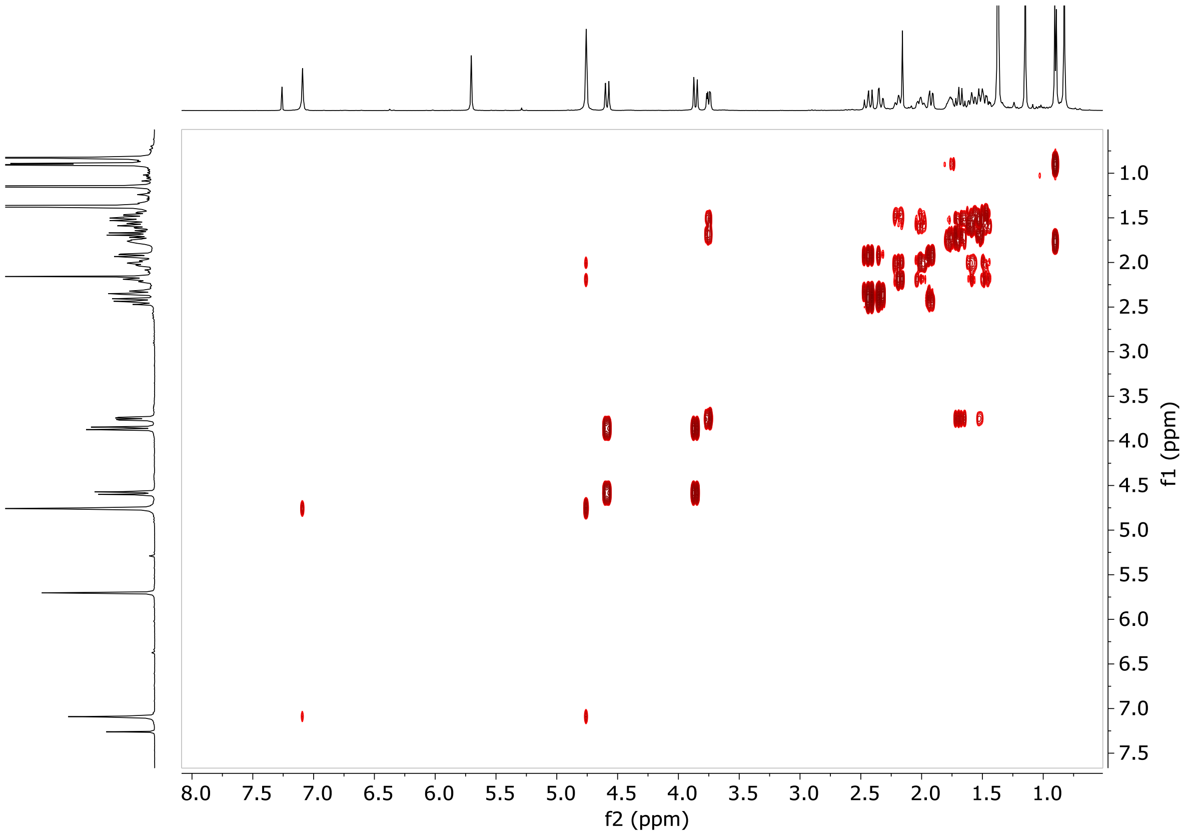

Figure S59. ^1H-^1H COSY spectrum of **9** ($CDCl_3$,700 MHz).

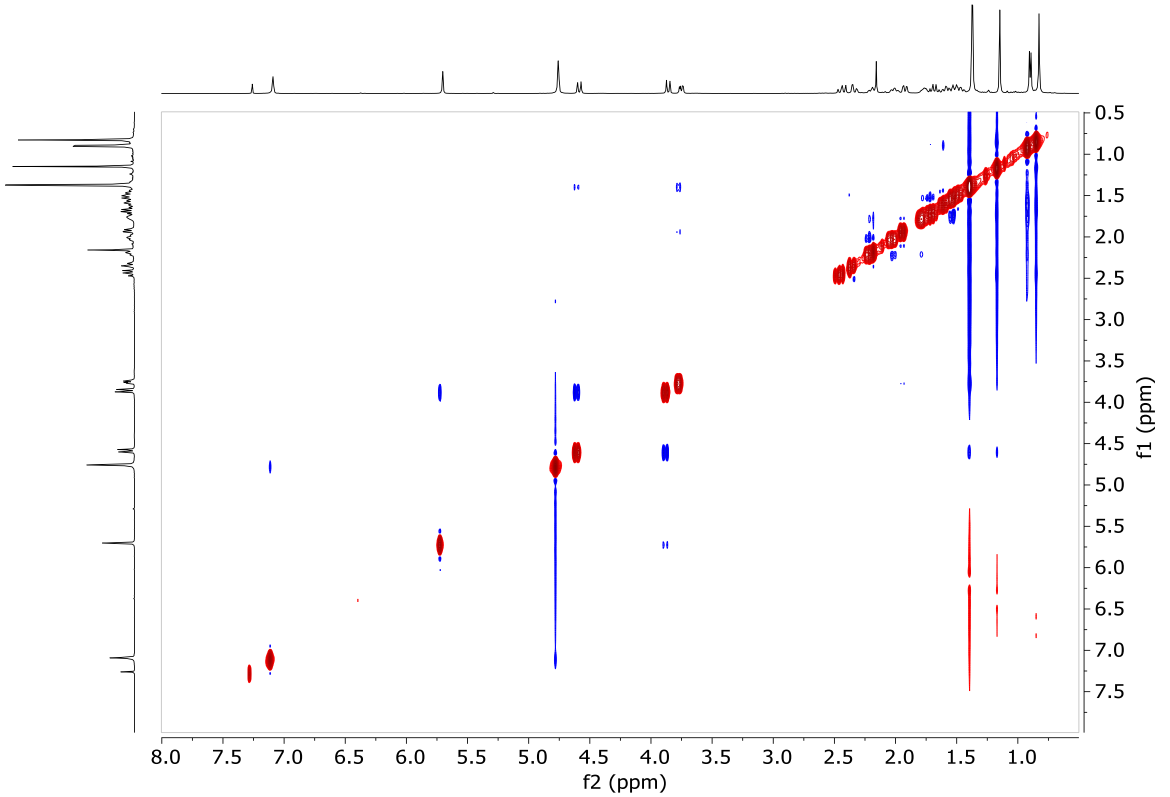

Figure S60. NOESY spectrum of **9** ($CDCl_3$,700 MHz).

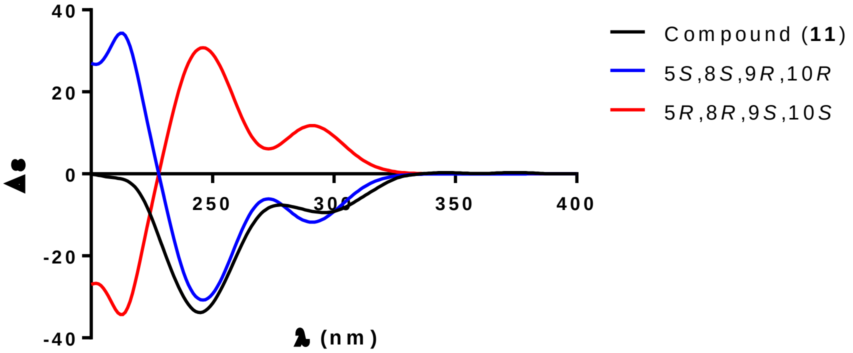

Figure S61. Comparison between the experimental (black line) and the calculated ECD spectra for the enantiomers (5S, 8S, 9R, 10R)-**11** (blue line) and (5R, 8R, 9S, 10S)-**11** (red line).

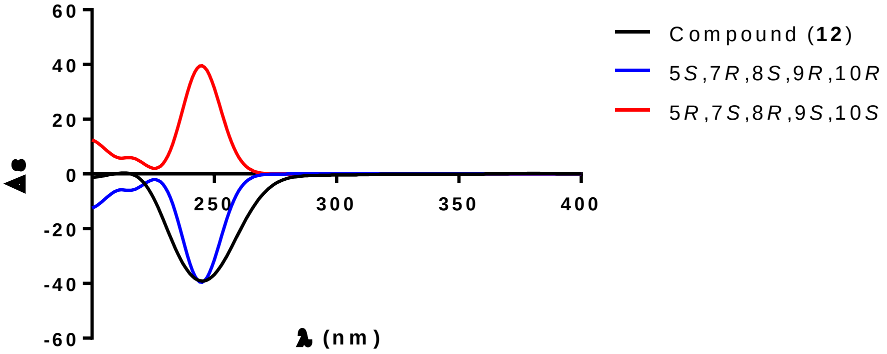

Figure S62. Comparison between the experimental (black line) and the calculated ECD spectra for the enantiomers (5*S*, 7*R*, 8S, 9*R*, 10*R*)-**12** (blue line) and (5*R*, 7*S*, 8*R*, 9S, 10*S*)-**12** (red line).

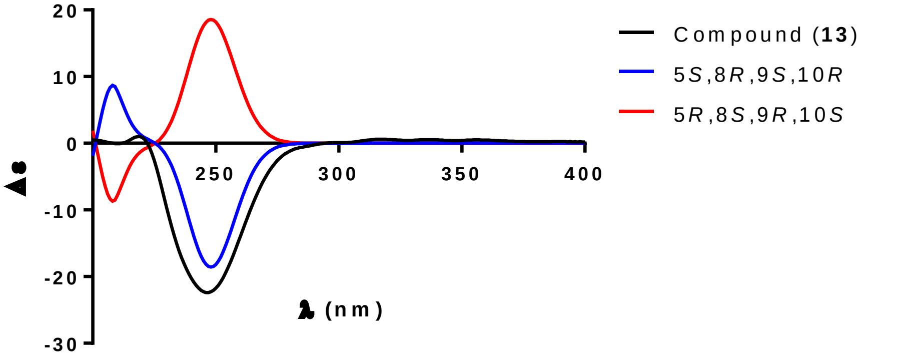

Figure S63. Comparison between the experimental (black line) and the calculated ECD spectra for the enantiomers (5S, 8R, 9S, 10R)-**13** (blue line) and (5*R*, 8S, 9R, 10S)-**13** (red line).

Table S1. Crystallographic data for compounds **1** and **10**.

Compound	1	10
Empirical formula	$C_{20} H_{32} O_{5}$	$C_{22} H_{28} O_{6}$
Formula weight	352.45	388.44
Temperature	100(2) K	100(2) K
Wavelength	1.54178 Å	1.54178 Å
Crystal system	Monoclinic	Orthorhombic
Space group	$P2_1$	$P2_12_12_1$
a	8.78990(10) Å	8.73010(10) Å
b	10.45200(10) Å	10.7299(2) Å
c	10.27570(10) Å	20.8669(3) Å
a	90°.	90°.
b.	90.2546(4)°.	90°.
g	90°.	90°.
Volume	944.040(17) Å3	1954.67(5) Å3
Z	2	4
Density (calculated)	1.240 Mg/m3	1.320 Mg/m3
Absorption coefficient	0.707 mm-1	0.782 mm-1
F(000)	384	832
Crystal size	0.433 x 0.405 x 0.290 mm3	0.401 x 0.319 x 0.278 mm3
Theta range for data collection	4.302 to 71.003°.	4.237 to 69.999°.
Index ranges	-10<=h<=10, -12<=k<=12, -12<=l<=11	-10<=h<=10, -13<=k<=13, -25<=l<=25
Reflections collected	33896	32163
Independent reflections	3601 [R(int) = 0.0304]	3709 [R(int) = 0.0384]
Completeness to theta = 67.679°	99.80%	100.00%
Absorption correction	Multi-scan	Multi-scan
Refinement method	Full-matrix least-squares on F2	Full-matrix least-squares on F2
Data / restraints / parameters	3601 / 6 / 242	3709 / 0 / 256
Goodness-of-fit on F2	1.048	1.055
Final R indices [I>2sigma(I)]	R1 = 0.0266, wR2 = 0.0725	R1 = 0.0266, wR2 = 0.0699
R indices (all data)	R1 = 0.0266, wR2 = 0.0725	R1 = 0.0267, wR2 = 0.0700
Absolute structure parameter Flack	-0.02(3)	0.01(5)
Absolute structure parameter Hooft	-0.02(2)	0.00(5)
Absolute structure parameter Parsons	-0.019(16)	0.00(3)
Largest diff. peak and hole	0.218 and -0.164 e.Å-3	0.165 and -0.157 e.Å-3

Table S2. Hydrogen bonds for **1** [Å and °].

Label	D-H...A	d(D-H)	d(H...A)	d(D...A)	<(DHA)
a	O(1)-H(1)...O(5)	0.836(13)	1.828(13)	2.6625(18)	175(3)
b	O(2)-H(2A)...O(1)#1	0.838(13)	1.884(14)	2.7150(18)	171(3)
c	O(5)-H(5A)...O(2)#2	0.849(13)	1.891(14)	2.7232(19)	166(3)
d	O(5)-H(5B)...O(3)#3	0.852(13)	1.935(14)	2.7730(18)	168(3)

Symmetry transformations used to generate equivalent atoms:
#1 -x+1,y+1/2,-z+1 #2 x+1,y,z #3 -x+2,y-1/2,-z+1

Table S3. Screening for antiproliferative activity of the isolated compounds from *Salvia guevarae* (25 μM) and dichloromethane extract of *S. guevarae* (25 ppm).

Compound/line cell	Antiproliferative activity (%)						
	U-251	PC-3	K562	HCT-15	MCF-7	SKLU-1	COS7
**1**	NC	NC	NC	6.8	3.5	6.4	13.8
**2**	0.1	12.5	43.3	13.1	23.7	7.5	NC
**1a**	35.2	23.6	34.0	10.1	10.3	15.7	9.7
**3**	NC	NC	26.5	NC	5.0	10.9	13.9
**5**	19.3	13.5	22.9	7.6	25.3	15.8	10.3
**6**	23.8	38.1	34.7	24.1	NC	10.9	NC
**7**	1.6	14.9	46.9	2.6	15.5	1.7	NC
**8**	3.9	5.6	23.5	4.9	4.0	4.1	2.0
**9**	NC	6.0	29.5	18.3	NC	8.5	8.7
**10**	NC	8.4	31.7	15.8	10.3	8.7	30.6
**11**	NC	2.2	27.2	16.6	9.8	7.9	3.9
**Dichloromethane extract of S. guevarae**	7.4	33.8	96.6	31.4	29.7	5.3	21.7

NC. No cytotoxicity was observed. The results are the average of three runs.

Protocol: Sulforhodamine B; Assay: Cytotoxicity in human cancer cell lines; Concentration: 25 μM; Vehicle: DMSO; Cell lines: U-251 (human glioblastoma), PC-3 (human prostatic adenocarcinoma), K562 (human chronic myelogenous leukemia), HCT-15 (human colorectal adenocarcinoma), MCF-7 (human mammary adenocarcinoma), cell lines were supplied by the National Cancer Institute (USA); and SKLU-1 (human lung adenocarcinoma) and COS-7 cell line (Healthy cells of monkey African green kidney) were donated by the Cancer Institute of Mexico. *The results are the average of three runs.

Table S4. Screening for inhibition of nitrite (NO) production for compounds **(1-3, 5-10)** in macrophages RAW 264.7 (25 μM).

Sample	Nitrites (μM)	Inhibition Nitrites (%)	Viability (%)
LPS+ MD	14.6 ± 0.6	100.0	100.0
LPS+ (DMSO)	14.6 ± 0.6	100.0	96.0 ± 2.7
1	15.7 ± 0.3	No Inhibition	95.4 ± 2.5
2	11.5 ± 0.4	21.0 ± 2.8	97.1 ± 2.9
3	15.5 ± 0.3	No Inhibition	90.7 ± 4.0
5	8.0 ± 0.1	45.1 ± 1.2	100.0 ± 0.3
6	6.2 ± 0.2	57.4 ± 1.6	85.0 ± 1.9
7	5.0 ± 0.2	66.1 ± 1.6	95.1 ± 2.8
9	17.8 ± 0.2	No Inhibition	84.0 ± 2.8
10	5.8 ± 0.1	60.0 ± 1.2	99.7 ± 0.3
Celecoxib (100 μM)	11.4 ± 0.3	21.9 ± 1.4	100
AMG (100 μM)	2.4 ± 0.1	83.6 ± 0.9	100

AMG: Aminoguanidine

Results are expressed as the mean ± S.E.M. of three determinations by triplicate.

Spectroscopic data of compounds 7-13.

6α-Hydroxy-patagonol acetonide (**7**): white powder; mp 130–132 °C; $[\alpha]^{25}_{D}$ -35.6 (c 0.0013, MeOH); UV (MeOH) λ_{max} (log ε) 212.0 (3.77) nm; ECD (*c* 3.47 mM, MeOH) $[\theta]_{209}$ +13852.4, $[\theta]_{221}$ -14248.2; IR (ATR) ν_{max} 2943, 2922, 2870, 1743, 1450, 1377, 1213, 1079, 1046, 999, 836 cm^{-1}, ^{1}H NMR ($CDCl_3$, 400 MHz) δ 7.08 (1H, p, *J* = 1.7 Hz, H-14), 5.42 (1H, t, *J* = 3.3 Hz, H-3), 4.76 (2H, q, *J* = 1.7 Hz, CH_2-15), 4.42 (1H, d, *J* = 13.0 Hz, H-18 *pro R*), 3.62 (1H, d, *J* = 13.0 Hz, H-18 *pro S*), 3.61 (1H, dd, *J* = 12.2, 3.7 Hz, H-6), 2.22 – 199 (3H, m, H-2, H-12a, H-12b), 1.73 – 1.56 (4H, m, H-1a, H-7a, H-8, H-11a), 1.57- 1.41 (3H, m, H-1b, H-7b, H-11b), 1.38 (1H, dd, *J* = 12.2, 1.8 Hz, H-10), 1.36 (3H, s, CH_3-2′), 1.34 (3H, s, CH_3-3′), 1.04 (3H, s, CH_3-19), 0.86 (3H, d, *J* = 6.6 Hz, CH_3-17), 0.75 (3H, s, CH_3-20); ^{13}C NMR (100 MHz, $CDCl_3$) δ 174.4 (C, C-16), 144.7 (C, C-4), 143.7 (CH, C-14), 135.0 (C, C-13), 125.0 (CH, C-3), 101.1 (C, C-1′), 74.6 (CH, C-6), 70.3 (CH_2, C-15), 65.4 (CH_2, C-18), 45.3 (CH, C-10), 42.5 (C, C-5), 38. 1 (C, C-9), 35.9 (CH_2, C-7), 35.6 (CH, C-8), 34.5 (CH_2, C-11), 26.6 (CH_2, C-2), 24.9 (CH_3, C-3′), 24.7 (CH_3, C-2′), 19.2 (CH_2, C-12), 18.5 (CH_3, C-20), 17.8 (CH_2, C-1), 16.4 (CH_3, C-19), 15.7 (CH_3, C-17); HR-ESI-MS *m/z* 375.2591 $[M+H]^+$; (calcd for $C_{23}H_{35}O_4$, 375.2530).

2α,6α-Dihydroxy-patagonol acetonide (**8**): white powder; mp 166–168 °C; $[\alpha]^{25}_{D}$ -36 (c 0.001, MeOH); UV (MeOH) λ_{max} (log ε) 217.8 (3.75) nm; ECD (*c* 2.56 mM, MeOH) $[\theta]_{212}$ +2968.4, $[\theta]_{221}$ 0, $[\theta]_{235}$ -4947.3; IR (ATR) ν_{max} 3226, 2984, 2950, 2931, 1738, 1451, 1218, 1082, 1051, 1000, 854 cm^{-1}; ^{1}H NMR ($CDCl_3$, 500 MHz) δ 7.10 (1H, p, *J* = 1.5 Hz, H-14), 5.41 (1H, t, *J* = 1.7 Hz, H-3), 4.78 (2H, q, *J* = 1.9 Hz, CH_2-15), 4.42 (1H, brd, *J* = 13.1, Hz, H-18 *pro R*), 4.40 (1H, t, *J* = 7.6 Hz, H-2), 3.65 (1H, d, *J* = 13.1 Hz, H-18 *pro S*), 3.58 (1H, dd, *J* = 11.9, 4.3 Hz, H-6), 2.17 (1H, m, H-12a), 2.03 (1H, m, H-12b), 2.00 (1H, dd, *J* = 11.7, 7.3 Hz, H-1), 1.70 (1H, ddd, *J* = 12.6, 6.6, 3.7 Hz, H-8), 1.65 – 1.58 (2H, m, H-7a, H-11a), 1.55 – 1.44 (3H, m, H-1b, H-7b, H-11b), 1.42 (1H, brd, J = 12.6 Hz, H-10), 1.36 (3H, s, CH_3-2′), 1.34 (3H, s, CH_3-3′), 1.10 (3H, s, CH_3-19), 0.86 (3H, d, *J* = 6.6 Hz, CH_3-17), 0.77 (3H, s, CH_3-20); ^{13}C NMR (125 MHz, $CDCl_3$) δ 174.4 (C, C-16), 147.1 (C, C-4), 143.9 (CH, C-14), 134.7 (C, C-13), 128.0 (CH, C-3), 101.3 (C, C-1′), 74.1 (CH, C-6), 70.3 (CH_2, C-15), 69.0 (CH, C-2), 65.0 (CH_2, C-18), 44.2 (CH, C-10), 42.9 (C, C-9), 37.8 (C, C-5), 35.8 (CH_2, C-7), 35.5 (CH, C-8), 34.2 (CH_2, C-11), 28.5 (CH_2, C-1), 24.8 (CH_3, C-2′), 24.6 (CH_3, C-3′), 19.2 (CH_2, C-12), 18.6 (CH_3, C-19), 16.3 (CH_2, C-20), 15.8 (CH_3, C-17); HR-ESI-MS *m/z* 391.2480 $[M+H]^+$; (calcd for $C_{23}H_{35}O_5$, 391.2479).

6α-Hydroxy-2-oxo-patagonol acetonide (**9**): white powder (EtOAc); mp 192–194 °C; $[\alpha]^{25}_{D}$ -32.2 (c 0.0009, MeOH); UV (MeOH) λ_{max} (log ε) 213 (3.8) nm; ECD (*c* 2.83 mM, MeOH) $[\theta]_{250}$ -51451.9, $[\theta]_{270}$ 0, $[\theta]_{301}$ +3298.2, $[\theta]_{341}$ -16491.0; IR (ATR) ν_{max} 3934, 2873, 1752, 1669, 1449, 1384, 1217, 1060, 825 cm^{-1}; ^{1}H NMR ($CDCl_3$, 500 MHz) δ 7.09 (1H, brs, H-14), 5.70 (1H, s, H-3), 4.76 (2H, brs, CH_2-15), 4.59 (1H, brd, *J* = 14.1, Hz, H-18 *pro R*), 3.86 (1H, d, *J* = 14.1 Hz, H-18 *pro S*), 3.75 (1H, dd, *J* = 11.9, 4.3 Hz, H-6), 2.44 (1H, dd, 17.6, 14.1 Hz, H-1a), 2.33 (1H, dd, 17.6, 3.7 Hz, H-1a), 2.19 (1H, m, H-12a), 2.01 (1H, m, H-12b), 1.92 (1H, dd, *J* = 14.1, 3.7 Hz, H-10), 1.77 (1H, m, H-8), 1.68 (1H, dt, 12.5, 12.4 Hz, H-11a), 1.59 (1H, ddd, 13.8, 12.9, 4.2 Hz, H-7a), 1.54 – 1.43 (2H, m, H-7b, H-11b), 1.38 (3H, s, CH_3-2′), 1.37 (3H, s, CH_3-3′), 1.15 (3H, s, CH_3-19), 0.90 (3H, d, *J* = 6.6 Hz, CH_3-17), 0.83 (3H, s, CH_3-20); ^{13}C NMR (125 MHz, $CDCl_3$) δ 200.3 (C, C-2), 174.3 (C, C-16), 167.6 (C, C-4), 144.1 (CH, C-14), 134.2 (C, C-13), 125.6 (CH, C-3), 101.6 (C, C-1′), 72.4 (CH, C-6), 70.4 (CH_2, C-15), 64.4 (CH_2, C-18), 44.7 (CH, C-10), 44.3 (C, C-5), 38.2 (C, C-9), 35.5 (CH, C-8), 35.0 (CH_2, C-7), 34.4 (CH_2, C-11), 39.9 (CH_2, C-1), 24.6 (CH_3, C-2′), 24.0 (CH_3, C-3′), 19.0 (CH_2, C-12), 18.0 (CH_3, C-20), 15.5 (CH_2, C-17), 14.4 (CH_3, C-20); HR-DART-MS *m/z* 389.2316 $[M+H]^+$; (calcd for $C_{23}H_{33}O_5$, 389.2328).

7*α*-Acetoxy-ent-clerodan-3,13-dien-18,19: 16,15-diolide (**10**): colorless crystals; mp 182-184 °C (reported [1] 178-179 °C); $[\alpha]^{25}_D$ – 107.5 (c 0.0016, MeOH) (reported – 110.6 ($CHCl_3$ [1])); ^{1}H NMR ($CDCl_3$, 700 MHz) *δ* 7.15 – 7.13 (1H, m, H-14), 6.76 (1H, dd, *J* = 7.5, 2.0 Hz, H-3), 5.34 – 5.32 (1H, m, H-7), 4.84 (1H, d, *J* = 8.0 Hz, H-19 *pro R*), 4.79 (2H, q, *J* = 1.7 Hz, CH_2-15), 3.95 (1H, dd, *J* = 8.0, 2.0 Hz, H-19 *pro S*), 2.46 – 2.39 (1H, m, H-2a), 2.30 (1H, dd, *J* = 14.8, 2.3 Hz, H-6*α*), 2.25 – 2.19 (2H, m, H-2b, H-12a), 2.10 (3H, s, CH_3-AcO), 2.07 – 2.00 (1H, m, H-12b), 1.89 (1H, qd, *J* = 7.1, 4.3 Hz, H-8), 1.85 (1H, dt, *J* = 13.0, 2.6, 1.8 Hz, H-1a), 1.81 (1H, d, *J* = 12.6 Hz, H-10), 1.65 – 1.59 (2H, m, CH_2-11), 1.48 (1H, ddd, *J* = 14.7, 3.9, 2.2 Hz, H-6*β*), 1.17 (1H, dtd, *J* = 12.6, 3.9 Hz, H-1b), 0.94 (3H, d, *J* = 7.1 Hz, CH_3-17), 0.85 (3H, s, CH_3-20); ^{13}C NMR (176 MHz, $CDCl_3$) *δ* 174.24 (C, C-16), 170.03 (C, COAc), 169.21 (C, C-18), 144.21 (CH, C-14), 138.32 (C, C-4), 136.07 (CH, C-3), 134.15 (C, C-13), 73.46 (CH, C-7), 72.21 (CH_2, C-19), 70.38 (CH_2, C-15), 48.13 (CH, C-10), 44.80 (C, C-5), 39.73 (CH, C-8), 38.61 (C, C-9), 38.17 (CH_2, C-6), 36.03 (CH_2, C-11), 27.75 (CH_2, C-2), 21.43 (CH_3, AcO), 19.30 (CH_2, C-12), 19.26 (CH_2, C-1), 18.99 (CH_3, C-20), 11.45 (CH_3, C-17); HR-DART-MS *m/z* 389.19499 [M+H]$^+$; (calcd for $C_{22}H_{29}O_6$, 389.19641).

7-Keto-*ent*-clerodan-3,13-dien-18,19: 16,15-diolide (**11**): white powder; mp 94-96 °C (reported [1] 89-93 °C); $[\alpha]^{25}_D$ – 53.3 (c 0.0012, $CHCl_3$) (reported – 168.42 ($CHCl_3$ [1])); ECD (*c* 2.90 mM, MeOH) $[\theta]_{244}$ +110819.5, $[\theta]_{299}$ -29683.8; ^{1}H NMR ($CDCl_3$, 500 MHz) *δ* 7.20 – 7.16 (1H, m, H-14), 6.87 (1H, dd, *J* = 7.4, 2.1 Hz, H-3), 4.81 (2H, d, *J* = 1.7 Hz, CH_2-15), 4.01 (1H, d, *J* = 8.2 Hz, H-19 *pro R*), 3.92 (1H , dd, *J* = 8.2, 2.2 Hz, H-19 *pro R*), 2.72 (1H, d, *J* = 12.5 Hz, H-6*α*), 2.64 (1H, q, *J* = 6.6 Hz, H-8), 2.55 – 2.48 (1H, m, H-2a), 2.44 (1H, d, *J* = 11.9 Hz, H-10), 2.38 (1H, dd, *J* = 11.9, 2.2 Hz, H-6b), 2.35 – 2.28 (2H, m, H-2b, H-12a), 2.26 – 2.18 (1H, m, H-12b), 1.95 (1H, dt, *J* = 13.1, 2.4 Hz, H-1a), 1.82 (1H, ddd, *J* = 14.9, 12.8, 4.5 Hz, H-11a), 1.60 (1H, td, *J* = 14.9, 13.0, 4.5 Hz, H-11b), 1.20 (1H, td, *J* = 12.4, 4.0 Hz, H-1b), 1.02 (3H, d, *J* = 6.7 Hz, CH_3-17), 0.64 (3H, s, CH_3-20); ^{13}C NMR (125 MHz, $CDCl_3$) *δ* 209.4 (C, C-7), 174.2 (C, C-16), 168.1 (C, C-18), 144.6 (CH, C-14), 137.5 (C, C-3), 133.6 (CH, C-13), 71.2 (CH_2, C-19), 70.5 (CH_2, C-15), 51.6 (CH-C-8), 50.8 (CH_2, C-6), 48.2 (C-C5), 48.0 (CH, C-10), 43.8 (C, C-9), 35.7 (CH_2, C-11), 27.5 (CH_2, C-2), 20.4 (CH_2, C-1), 19.6 (CH_2, C-12), 19.1 (CH_3, C-20), 7.9 (CH_3, C-17); HR-DART-MS *m/z* 345.16962 [M+H]$^+$; (calcd for $C_{20}H_{25}O_5$, 345.17020).

7*α*-Hydroxy-*ent*-clerodan-3,13-dien-18,19: 16,15-diolide (**12**): white powder; mp 102-104 °C (reported [1] 178-179 °C); $[\alpha]^{25}_D$ – 88 (c 0.001, $CHCl_3$) (reported – 154.54 ($CHCl_3$ [1])); ECD (*c* 2.89 mM, MeOH) $[\theta]_{244}$ -130608.7; ^{1}H NMR ($CDCl_3$, 700 MHz) *δ* 7.15 – 7.12 (1H, m, H-14), 6.73 (1H, dd, *J* = 7.5, 2.1 Hz, H-3), 5.28 (1H, d, *J* = 7.6 Hz, H-19 *pro R*), 4.79 (2H, q, *J* = 1.8 Hz, CH_2-15), 3.91 (1H, dd, *J* = 7.6, 2.2 Hz, H-19 *pro S*), 2.43-2.38 (1H, m, H-2a), 2.36 (1H, dd, *J* = 14.2, 2.2 Hz, H-6*α*), 2.25 – 2.17 (2H, m, H-2b, H-12a), 2.03 (1H, dddd, *J* = 14.9, 12.8, 4.4, 2.3 Hz, H-12b), 1.78 (2H, br d, *J* = 11.6 Hz, H-10, H 1a), 1.74 (1H, qd, *J* = 7.1, 3.8 Hz, H-8), 1.65 – 1.56 (2H, m, CH_2-11), 1.41 (1H, ddd, *J* = 14.1, 3.5, 2.3 Hz, H-6*β*), 1.15 (1H, td, *J* = 12.8, 3.9 Hz, H-1b), 1.07 (3H, d, *J* = 7.2 Hz, CH_3-17), 0.88 (3H, s, CH_3-20); ^{13}C NMR (176 MHz, $CDCl_3$) *δ* 174.3 (C, C-16), 170.0 (C, C-18), 144.0 (CH, C-14), 139.2 (C, C-4), 135.4(CH, C-3), 134.4 (C, C-13), 72.8 (CH_2, C-19), 72.8 (CH, C-7), 70.4 (CH_2, C-15), 48.4 (CH, C-10), 45.1 (C, C-5), 40.7 (CH, C-8), 40.6 (CH_2, C-6), 38.6 (C, C-9), 36.3 (CH_2, C-11), 27.8 (CH_2, C-2), 19.4 (CH_2, C-1), 19.3 (CH_3, C-20), 19.3 (CH_2, C-12), 12.1 (CH_3, C-17); HR-DARTMS m/z 347.18506 [M+H]$^+$; (calcd for $C_{22}H_{27}O_5$, 347.18585).

***neo*-Clerodan-3,13-dien-16,15:18,19-diolide** (**mkapwanin**) (**13**): white powder; (reported [2] Colorless oil); mp 180-182 °C; $[\alpha]^{25}_D$ – 74 (c 0.0005, CH_2Cl_2) (reported $[\alpha]^{27}_D$ – 11.7 (CH_2Cl_2) [2])); ECD (*c* 1.51 mM, MeOH) $[\theta]_{246}$ -74209.5; ^{1}H NMR ($CDCl_3$, 500 MHz) *δ* 7.13 (1H, p, *J* = 1.6 Hz, H-14), 6.77 (1H, dd, *J* = 7.4, 2.2 Hz, H-3), 4.79 (2H, q, *J* = 1.9 Hz, CH_2-15), 4.30 (1H, d, *J* = 8.1 Hz, H-19 *pro R*), 3.92 (1H, dd, *J* = 8.1, 2.1 Hz, H-19 *pro S*), 2.43 – 2.36 (1H, m, H-2a), 2.33 – 2.20 (2H, m, H-2b, H-12a), 2.087 – 1.96 (1H, m, H-12b), 1.94 (1H, ddd, *J* = 13, 3.2 Hz, H-6*α*), 1.77 (1H, m, H-1a), 1.76 (1H, m, H-10), 1.70 (1H, m, H-8), 1.64 (1H, m, H-7a), 1.64 – 1.59 (2H, m, CH_2-11), 1.50 (1H, m, H-7b), 1.25 (1H, m, H-6*β*),

1.08 (1H, m H-1b), 0.86 (3H, d, *J* = 6.7 Hz, CH_3-17), 0.62 (3H, s, CH_3-20); ^{13}C NMR (125 MHz, $CDCl_3$) δ 174.4 (C, C-16), 169.5 (C, C-18), 144.0 (CH, C-14), 138.4 (C, C-4), 136.0 (CH, C-3), 134.6 (C, C-13), 71.9 (CH_2, C-19), 70.4 (CH_2, C-15), 48.2 (CH, C-10), 45.7 (C, C-5), 38.9 (C, C-9), 36.7 (CH, C-8), 35.3 (CH_2, C-11), 34.6 (CH_2, C-6), 27.9 (CH_2, C-2), 27.8 (CH_2, C-2), 19.7 (CH_2, C-1), 19.2 (CH_2, C-12), 17.8 (CH_3, C-20), 15.8 (CH_3, C-17); HR-DART-MS m/z 331.18984 $[M+H]^+$; (calcd for $C_{20}H_{27}O_4$, 331.19093).

References

1. Esquivel, B.; Hernandez, L.M.; Cardenas, J.; Ramamoorthy, T.P.; Rodriguez-Hahn, L. Further *ent*-Clerodane Diterpenoids from *Salvia melissodora*. Phytochemistry 1989, 28, 561–166.
2. Omosa, L.K.; Midiwo, J.O.; Derese, S.; Yenesew, A.; Peter, M.G.; Heydenreich, M. *Neo*-Clerodane Diterpenoids from the Leaf Exudate of *Dodonaea angustifolia*. Phytochem Lett 2010, 3, 217–220, doi:10.1016/j.phytol.2010.08.001.
